# Seroprotection against tetanus in southern Vietnam

**DOI:** 10.1016/j.vaccine.2023.02.036

**Published:** 2023-03-24

**Authors:** C. Louise Thwaites, Tran Tan Thanh, Nguyen Thi Han Ny, Lam Anh Nguyet, Nguyen Thi Duy Nhat, Cao Thu Thuy, Nguyen Thi Le Thanh, Nguyen Thanh Dung, OUCRU Sero-surveillance Network, James Campbell, Pham Quang Thai, Le Van Tan, Marc Choisy, Maciej F. Boni

**Affiliations:** a Oxford University Clinical Research Unit, Ho Chi Minh City, Viet Nam; b Centre for Global Health and Tropical Medicine, University of Oxford, UK; c Hospital for Tropical Diseases, Ho Chi Minh City, Viet Nam; d National Institute of Hygiene and Epidemiology, Hanoi, Viet Nam; e School of Preventive Medicine and Public Health, Hanoi Medical University, Hanoi, Viet Nam; f Center for Infectious Disease Dynamics, Department of Biology, Pennsylvania State University, University Park, PA 16802, United States

**Keywords:** Tetanus, Seroprotection, Vietnam, Low middle income country, Vaccination, EPI

## Abstract

**Background:**

Ongoing tetanus cases and sporadic outbreaks of vaccine-preventable diseases associated with routine vaccination programmes remain problems in many low and middle-income countries, including Vietnam. With no human-to-human transmission or natural immunity, tetanus antibody levels indicate both individual risk of tetanus and gaps in vaccination programmes.

**Methods:**

To investigate gaps in immunity to tetanus in Vietnam, a country with a historically high level of tetanus vaccination coverage, tetanus antibodies were measure by ELISA from samples selected from a long-term serum bank, established for the purposes of general-population seroepidemiological investigations in southern Vietnam. Samples were selected from 10 provinces, focussing on age-groups targeted by national vaccination programmes for infants and pregnant women (Expanded Programme on Immunization, EPI, and Maternal and Neonatal Tetanus, MNT).

**Results:**

Antibodies were measured from a total of 3864 samples. Highest tetanus antibody concentrations occurred in children under 4 years old, over 90 % of whom had protective levels. Approximately 70 % of children aged 7–12 years had protective antibody concentrations although there was variation among provinces. For infants and children, there were no significant differences in tetanus protection between males and females, but for adults aged 20–35 years, in five of the ten provinces surveyed, protection against tetanus was higher in females (p < 0.05) who are eligible for booster doses under the MNT programme. In seven of ten provinces, antibody concentrations were inversely related to age (p < 0.01) and protection of older individuals was generally low.

**Conclusion:**

Widespread immunity to tetanus toxoid is seen in infants and young children consistent with the high coverage rates reported for diptheria tetanus toxoid and pertussis (DTP) in Vietnam. However, the lower antibody concentrations seen in older children and men suggest reduced immunity to tetanus in populations not targeted by EPI and MNT programmes.

## 1 Introduction

Routine vaccination programmes already prevent up to 3.5 million deaths a year and deliver huge economic benefits to societies across the world [1]. Nevertheless, increasing vaccination coverage in low and middle income-countries (LMICs) might prevent an additional 1.5 million deaths a year, with additional positive health and socio-economic impacts for individuals and communities [1]. To achieve this, it is necessary to understand where gaps in vaccination coverage lie.

Vaccination against tetanus is inexpensive and effective. Immunoglobulin G (IgG) antibodies produced in response to vaccination with a tetanus toxoid containing vaccine (‘tetanus antibodies’) are able to neutralize tetanus toxin produced following infection with *Clostridium tetani,* thereby preventing disease. Tetanus vaccination has been a principal component of Vietnam’s Expanded Programme on Immunization (EPI) schedule since the programme was introduced in 1981 [2]. Usually given as polyvalent vaccines within routine vaccination programmes, a 3-dose primary course of a tetanus toxoid-containing vaccine is given to infants at 2, 3, and 4 months of age. A 4th dose at 18 months was added in 2011 [3]. Following this primary course, two or three boosters are recommended to confer long-term immunity.

Unlike other vaccine-preventable diseases, tetanus does not spread from person to person (directly or indirectly) and natural infection confers no immunity. Individuals will only have protective antibody titres if they themselves (or, in the case of neonates, their mothers) have been vaccinated. Evaluating tetanus antibodies therefore indicates both immunity to tetanus and coverage of vaccination programmes. An in-vivo neutralisation assay is considered the ‘gold standard’ method of detecting tetanus antibodies. Enzyme-linked immunosorbent assay (ELISA) or immunofluorescence are more simple and cheaper to perform, but lack specificity for neutralizing antibody, particularly at low antibody concentrations. Indirect ELISA may over-estimate neutralizing anti-tetanus antibody levels below 0.16 IU/ml. Based on historical data and extrapolation of data from animal experiments, tetanus antibody concentrations of 0.01 IU or more measured by toxin neutralization are accepted as providing protection against tetanus. If ELISA is used, a higher ‘protective’ threshold of 0.1 IU/ml is accepted. Nevertheless, it is likely that protection is also influenced by amount and rate of toxin production as well as absolute antibody titres. One of the primary metrics of tetanus protection in the EPI program is the percentage of one-year-olds who have received three doses of a combined diphtheria, tetanus toxoid, and pertussis vaccine (DTP3) in a given year. In Vietnam, like most low and middle income countries (LMICs), resources to deliver and record the subsequent boosters required for longer term protection are limited and booster doses are recommended rather than included in national vaccination programmes. An exception to this is the highly successful maternal and neonatal tetanus (MNT) programme, introduced in 1993, where vaccination of pregnant women (or all women of childbearing age in high risk areas) with 2 doses of tetanus toxoid containing vaccine resulted in elimination of neonatal tetanus in Vietnam in 2005.

Vietnam’s high rates of DTP3 coverage and its success in eliminating MNT are important public health achievements. Since 1992 the country has reported DTP3 vaccination coverage rates of over 90 % with the exception of two years: in 2002 vaccine supplies limited coverage and in 2013 public concern over side effects affected uptake [4]. Nevertheless, several hundred children and adults are still hospitalized each year with tetanus and there have been recent outbreaks of diphtheria in some regions, indicating that routine vaccination programmes are still missing some sections of the population [5], [6], [7], [8].

Precise knowledge of which individuals are insufficiently protected enables efficient targeting of resources and public health strategies, however vaccination-recall, population relocation and lack of accurate electronic health record data make this difficult in LMICs such as Vietnam. In our study, we aimed to make use of a large serum bank of cross-sectionally collected general-population samples, to measure anti-tetanus toxoid antibodies in specific age groups and locations in order to identify populations at risk of tetanus.

## 2 Methods

The study was carried out at the Oxford University Clinical Research Unit, Ho Chi Minh City using samples from a long-term serum bank, which has collected samples from 10 provinces since its establishment in 2009, for the purposes of conducting general-population seroepidemiological investigations in southern and central Vietnam [6], [9], [10], [11], [12], [13]. The study was approved by the Ethical Committee of the Hospital for Tropical Diseases and Oxford Tropical Research Ethics Committee.

**Fig. 1:**
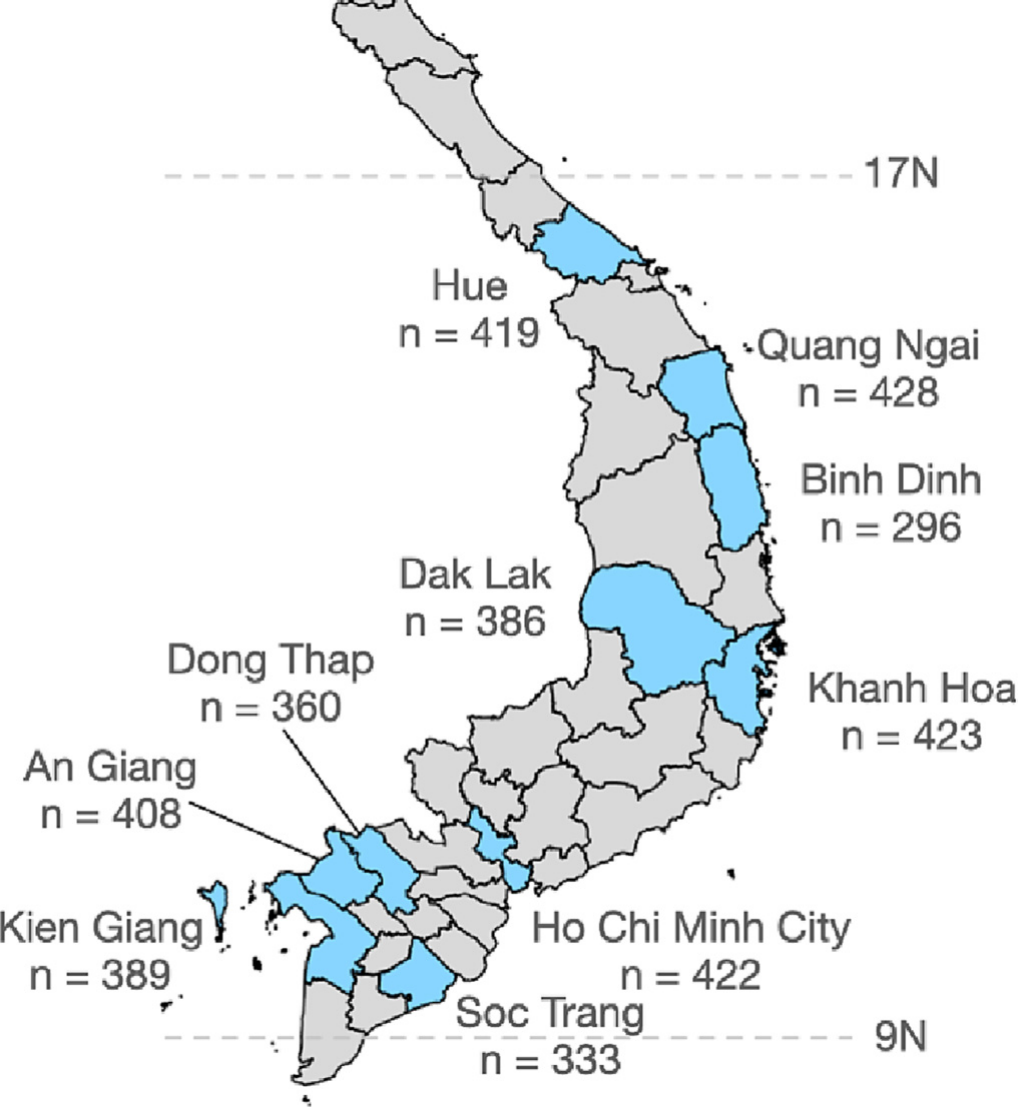
Map showing number of samples collected from each site in southern Vietnam.

Samples were selected from samples collected between 1st January 2012 and 30th June 2016 from all 10 provinces contributing to the serum bank: Ho Chi Minh City, Khanh Hoa, Thua Thien Hue, Dak Lak, Kien Giang, Soc Trang, An Giang, Dong Thap, Quang Ngai, and Binh Dinh. Except for Ho Chi Minh City, these were predominantly mixed urban/rural provinces [See Fig. 1]. Serum bank samples were stored at −20 °C. Our study specifically aimed to understand tetanus protection in those targeted by routine EPI and MNT vaccination programmes (i.e. vaccination of neonates, young children and women of childbearing age). Additionally, we hoped to gain insight into tetanus protection in those for whom booster doses were recommended but not routinely provided (older children and men). Given uncertainties about accuracy of specific cut-off's for tetanus protection, we report absolute antibody titres, as well as include a 0.1 IU cut-off for reference and to aid interpretation. Our sample size was based upon published DTP3 coverage in Vietnam [14]. Consequently, our sample size was chosen to provide equal numbers samples from males and females and from specific age-groups as follows: <2 years (50 samples per site); 7–12 years (110 samples per site): 20–35 years (75 samples total, 5 samples per single-year age band per site); 36–90 years (165 samples total, 3 per single-year age band per site). The “<2” age group was chosen to assess vaccination coverage of the standard DTP3 EPI schedule in infants (2, 3, 4, months with/without a booster at 18 months). As insufficient samples in this age group were available in Dak Lak province, the inclusion criterion was broadened to “<4” to achieve a sample size of 50. We report this age group as “<4” in the Results section, and for 9/10 sites all included samples are under two years of age. The 7–12 age-group was selected to investigate whether recommended but not routine boosters of 5–6 year olds were likely to be occurring. The 20–35 year age group was selected to investigate sex differences which may be due to MNT vaccination. Those over 35 years of age (born before 1981) were born before the EPI introduction although some women may have received MNT programme vaccination. A sample size of 50 allows estimation of a 50 % proportion with 95 % confidence limits of 35 % to 65 %, and a 90 % proportion with 95 % confidence limits of 82 % to 98 % (exact binomial method). No criteria were set for minimizing type I or type II error as inferred IU/ml measurements from ELISA (see below) were viewed as correctly inferred either above or below the 0.1 IU/ml threshold for protection.

Anti-tetanus toxoid antibodies were measured by indirect ELISA as previously described [14], [15], [16].

Positive controls were included in duplicate on each plate, with 12 2-fold dilutions ranging from 1:50 to 1:102400. A logistic curve was fit to the means of the two duplicate positive-control OD values (least squares, across all 12 dilutions) to identify the positive-control dilution corresponding to an optical density of 2 (on a scale of 0 to 4). An identical logistic fit was carried out for the three sample dilutions, and the ratio of dilution values corresponding to OD = 2 was used to calculate the IU/ml antitoxin antibody value of each patient sample. All dilution curves were inspected manually to ensure the logistic curve fit resulted in an appropriate IU/ml estimate for each patient sample. Differences in IU/ml values between males and females were tested with a Mann-Whitney test.

For each site, we explored the relationship between log(IU) and age using locally estimated scatterplot smoothing (LOESS) regression (with polynomial degrees 2 and degree of smoothing of 2) as a smoother in order to highlight the trend [17]. To evaluate possible variation in antibody concentrations related to admitting ward, for each site, we selected admitting wards contributing >10 samples to the analyses. Then, for each age category, we performed a recursive partitioning for each site [1] based on ANOVAs with IU as response and ward as explanatory variable [18]. P Values of 0.05 were taken as significant.

Analyses were performed in R Version 4.1.1 [R Foundation for Statistical Computing, Vienna, Austria] and MATLAB 2019b, (The MathWorks, Inc., Natick, Massachusetts, United States). A cut-off of 0.1 IU/ml was used to signify protective anti-tetanus toxoid concentrations [3].

## 3 Results

Antibodies were measured from a total of 3864 samples. Due to individual site variation, small deviations from the proposed sample size occurred (Supplementary Table 1).

**Fig. 2:**
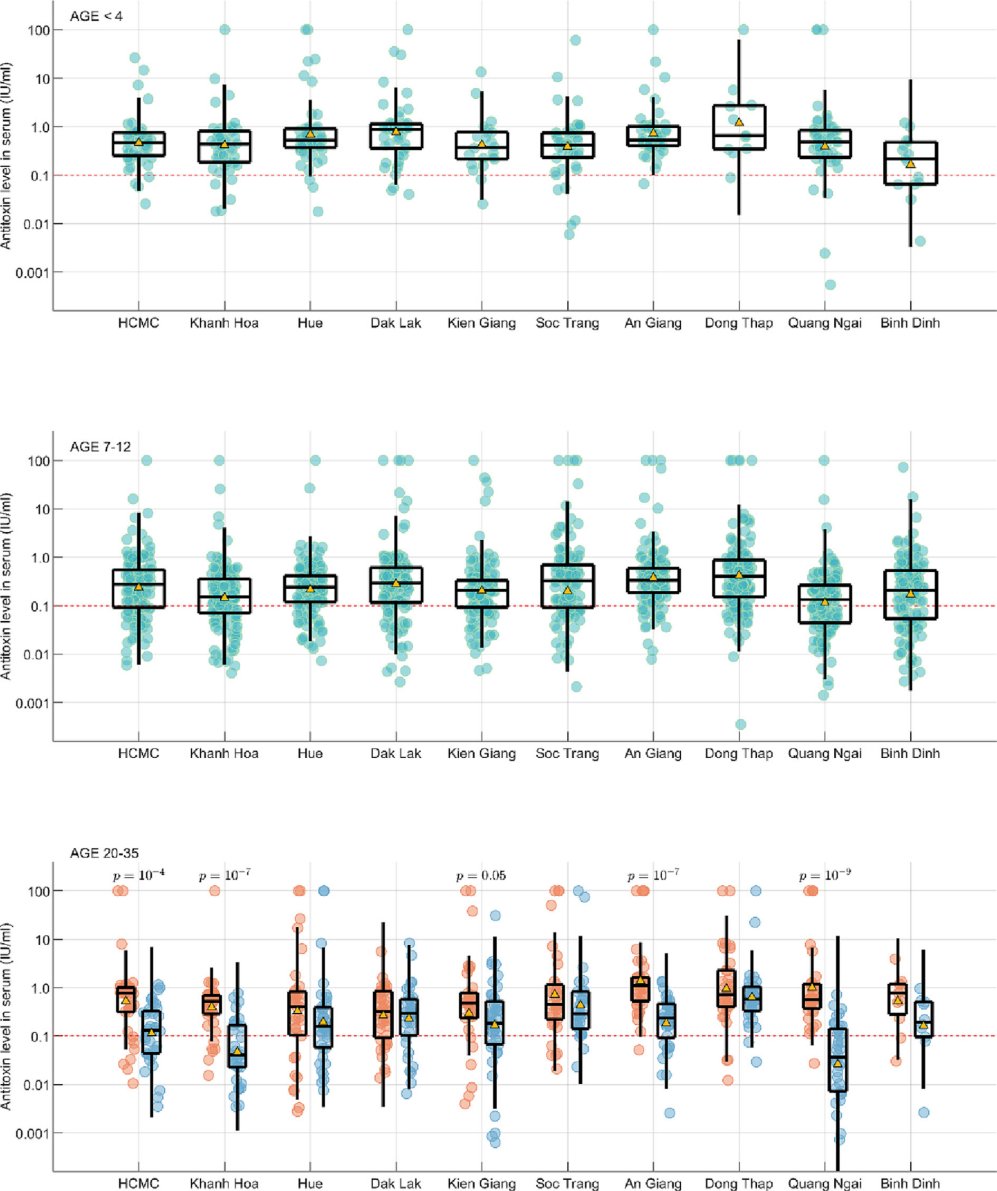
Anti-tetanus toxin antibody concentrations in 3 selected age groups. Top: < 4 years old; middle:7–12 years old and bottom, 20–35 years old, disaggregated according to sex, females (red) and males (blue). All points plotted in green, red, or blue. Boxplots show medians and interquartile (IQR) ranges and whiskers show 1.5 times the IQR. In the bottom panel, p-values are shown where IU/ml values are significantly different between men and women. Yellow triangles show geometric mean IU/ml value for each subsample. (For interpretation of the references to colour in this figure legend, the reader is referred to the web version of this article.)

Concentrations of tetanus antibodies for the three age-groups targeted by national vaccination program (all born since the introduction of the EPI programme) are shown in Fig. 2. Highest antibody concentrations occurred in those < 4 years, of whom over 90 % had protective levels **(**Table 1**)**, indicating that during 2010–2014, the years when the children in the sample would have received immunization, DTP3 coverage was high. The one exception is Binh Dinh province where both a low sample size and low antibody levels result in high uncertainty and an estimate of 60.0 % (95%CI:32.3 % − 83.7 %) DTP3 coverage.

**Table 1:** Percent (95 % CI) of each age/gender group protected against tetanus (>0.1 IU/ml). No significant differences were noted between genders in the two younger age groups. For the 20–35 age group, statistically higher protection among females than males is shown in bold (Mann-Whitney test, p = 0.05).

	Age < 4	Age 7–12	Age 20–35, Female	Age 20–35, Male
Ho Chi Minh City	92.0 % (80.8 %–97.8 %)	70.7 % (62.4 %–78.1 %)	**78.9 % (62.7 %–90.4 %)**	**56.8 % (39.5 %–72.9 %)**
Khanh Hoa	90.0 % (78.2 %–96.7 %)	63.6 % (55.0 %–71.5 %)	**85.4 % (70.8 %–94.4 %)**	**32.4 % (17.4 %–50.5 %)**
Hue	94.0 % (83.5 %–98.7 %)	79.3 % (71.6 %–85.7 %)	77.1 % (59.9 %–89.6 %)	69.2 % (52.4 %–83.0 %)
Dak Lak	96.8 % (83.3 %–99.9 %)	79.9 % (72.8 %–85.8 %)	75.0 % (58.8 %–87.3 %)	74.3 % (56.7 %–87.5 %)
Kien Giang	90.9 % (70.8 %–98.9 %)	72.9 % (64.7 %–80.0 %)	**79.4 % (62.1 %–91.3 %)**	**70.7 % (54.5 %–83.9 %)**
Soc Trang	88.4 % (74.9 %–96.1 %)	73.5 % (64.3 %–81.3 %)	85.3 % (68.9 %–95.0 %)	89.7 % (72.6 %–97.8 %)
An Giang	98.0 % (89.1 %–99.9 %)	87.9 % (81.3 %–92.8 %)	**97.4 % (86.2 %–99.9 %)**	**75.7 % (58.8 %–88.2 %)**
Dong Thap	90.9 % (58.7 %–99.8 %)	79.5 % (71.7 %–86.1 %)	91.2 % (76.3 %–98.1 %)	93.9 % (79.8 %–99.3 %)
Quang Ngai	90.0 % (78.2 %–96.7 %)	54.7 % (46.3 %–62.9 %)	**97.1 % (85.1 %–99.9 %)**	**34.2 % (19.6 %–51.4 %)**
Binh Dinh	60.0 % (32.3 %–83.7 %)	63.8 % (55.2 %–71.8 %)	81.8 % (48.2 %–97.7 %)	70.0 % (34.8 %–93.3 %)

Antibody concentrations in children 7–12 years old showed more variation between sites. Approximately 70 % of children in this age-group had protective antibody concentrations, indicating either a lower level of boosters given to children, or a lower rate of infant vaccination during 2000–2008, the birth years of the children sampled in the 7–12 years cohort. Three sites (Khanh Hoa, Quang Ngai, Binh Dinh) had seroprotection lower than 65 % in this age-group, indicating there may be lower reach of the health systems in these provinces than in other parts of southern and central Vietnam.

For infants and children, there were no significant differences in tetanus protection according to sex, but for adults aged 20–35 years, in five provinces, protection against tetanus was lower in males than females (eligible for booster doses under the MNT programme). In the provinces with the widest gaps between protection in males and females, protection rates in females were higher than those in the 7–12 age-group indicating that additional boosters were likely received by females through the MNT programme.

**Fig. 3:**
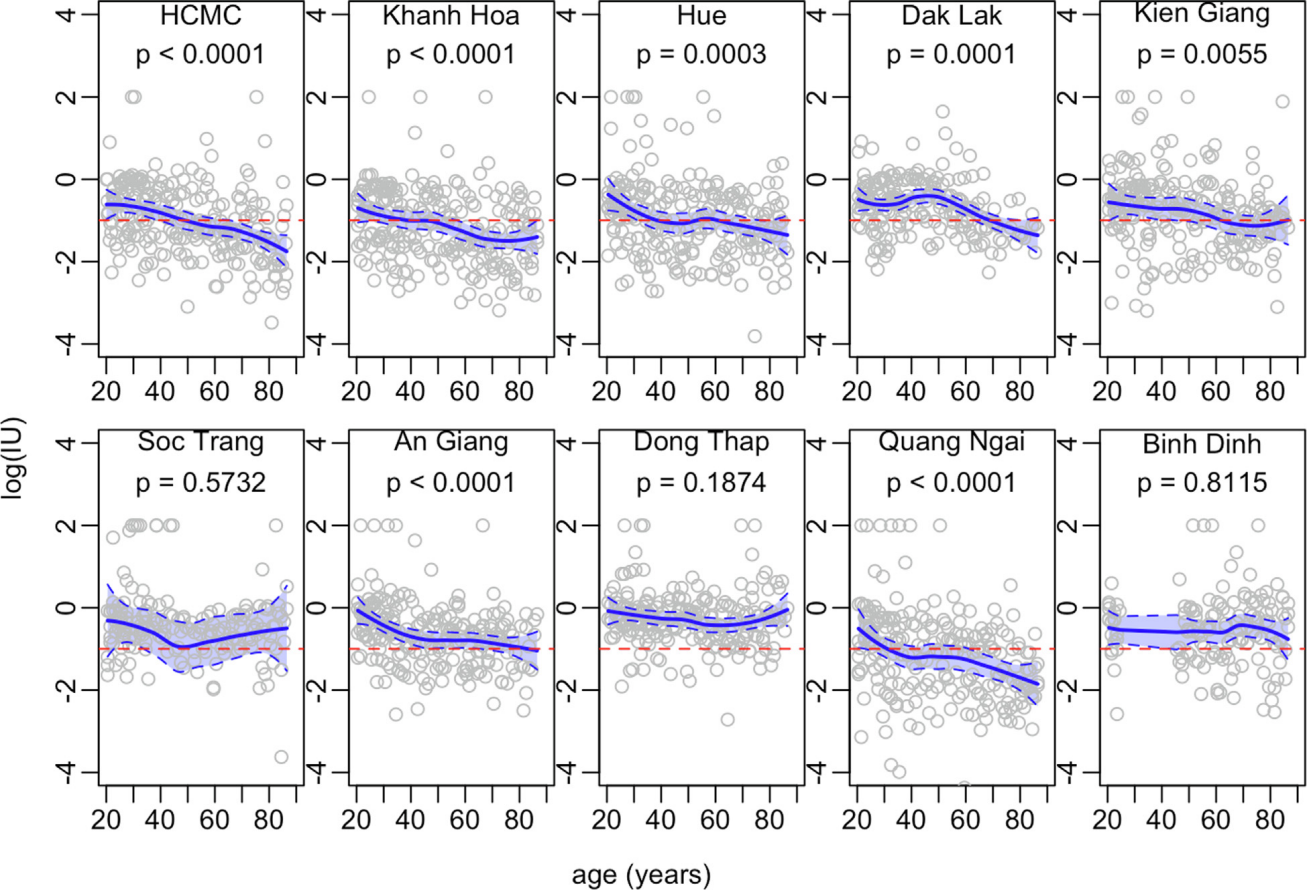
Antibody concentration for the 20–80 year age group by province with 95 % confidence intervals (shaded area) produced using LOESS regression [17].

Fig. 3 shows antibody concentrations of all adults in our sample. In most provinces, antibody concentrations are inversely related to age, and protection of older individuals is generally low. Three provinces (Soc Trang, Dong Thap and Binh Dinh) do not show this inverse relationship, and the majority of the population in Soc Trang and Dong Thap have protective antibody levels which may indicate either effective boosting or primary vaccination of older individuals. The pattern in Binh Dinh is the most unusual among the ten provinces, suggesting adequate coverage by the MNT programme but inadequate coverage of EPI. Sex-disaggregated data are shown in Supplementary Fig. 1. The data show similar downward trends in antibody titres with age for both males and females, but with a more obvious decrease for women. Notably the proportion of the population with protective antibodies appears similar in those over 60 years.

To examine potential bias due related to collection sites, the confounding effect of admitting department from which the sample originated was evaluated [Supplementary Fig. 2]. Whilst there was some variation in antibody concentrations between different departments within 5 sites, these did not conform to a consistent pattern.

## 4 Discussion

Our paper presents tetanus immunity across different age groups in samples from a multi-province nationwide serum bank. Our sample age structure was particularly chosen to examine the routine vaccination programmes of EPI and MNT where vaccination is focussed on pregnant women, infants and young children. Our results demonstrate high rates of protective anti-tetanus toxoid antibody titres in the specific groups targeted by these programmes, indicating high coverage of these programmes. However, rates of protective antibody titres were generally lower in the population not targeted by these programmes, suggesting that outside of EPI and MNT programmes, there is insufficient tetanus vaccination.

Our data derive from residual blood samples contributed to a serum bank. They are therefore subject to potential bias related to the population and site from whom they were collected [9]. Nevertheless, our main findings are seen consistently in subgroups of individual sites, and exhibit characteristic patterns observed in other LMIC settings where tetanus vaccination programmes are focussed around DTP3 and MNT vaccinations. Furthermore, they are in agreement with the age and sex distribution of cases of tetanus admitted to the tertiary referral centre for tetanus treatment in southern Vietnam where most cases occur in men or older women [19]. Whilst we observe highest antibody concentrations in young children (<4 years old) and women of child-bearing age, explaining the lower antibody concentrations observed in other age-groups is difficult due to lack of metrics concerning booster vaccination in Vietnam. Both boosters and post-exposure vaccination are recommended in Vietnam, but not included in routine vaccination programmes. Whilst children aged 7–12 years generally have lower antibody levels than those aged < 3 years, this may be due to either low primary vaccination in years 2000–2008 or lack of booster doses. Of note, except for 2002, when the national vaccination programme was impacted by restricted vaccine supply, overall Vietnam’s DTP3 rates have been > 90 % during these years [20], indicating that primary vaccination rates in this cohort were likely to be high.

We note some significant differences in antibody concentrations between provinces. This may reflect true variation in coverage of programmes or be due to differences in our serum bank samples. The serum bank was conceived to collect residual blood samples from participating provincial-level hospitals. It therefore is subject to possible bias in terms of patients admitted and participating sampling departments. Our evaluation of admitting ward differences shows that, in some sites, there were differences in antibody concentrations in some sites which might indicate that there were differences in populations between provinces, however these appeared to be only significant in terms of protection/no-protection for two sites (Khanh Hoa and Ho Chi Minh City). Whilst the lack of additional data for specimens is a limitation in interpreting our study, the simplicity and ease the sample collection process has allowed the sustainable development of the serum bank, providing a valuable public health resource.

We observe a decline in anti-tetanus antibody concentrations with increasing age, indicating lower protection in older age-groups. Reasons for this may be the relatively recent introduction of routine vaccination to Vietnam as well as natural decline in antibody concentrations in the absence of booster doses. A pattern of waning immunity over time is clearly documented in high-income settings where high levels of primary vaccination coverage have been achieved and is one of the key features underpinning recommendations for either serial boosting or a total of 5 –6 tetanus-toxin containing vaccinations to provide necessary long-term immunity. Nevertheless it is important to note that many adults before the introduction of national vaccination programmes had protective antibody levels, indicating that additional vaccination opportunities are being used, or have been in the past.

A further potential limitation of our study is the use of ELISA to measure antibody concentrations. Whilst, this is a commonly employed method, values can show some discrepancies, particularly at low levels. In the absence of a standard ELISA methodology, we have given a full description of our methodology. It is important to note that the ELISA method measures in vitro antibodies directed against a standard tetanus toxin. Lack of specificity may be due to detection on non-neutralizing antibodies. Inaccuracies may be greater after recent infection or vaccination. Nevertheless, the patterns we report here are consistent with overall expected impact of vaccination programmes and reported national-level data. We have employed the conservative cut-off of for protection of 0.1 IU/ml recommended for ELISA in view of inaccuracies at low levels, however it is possible that many of those we classify as ‘unprotected’ will have some protection using a lower cut-off for other methodologies of 0.01 IU/ml [3]. However, our main aim of the paper is to equate patterns of antibody concentrations with likely vaccination coverage, rather than absolute tetanus protection.

## 5 Conclusions

Our study explores tetanus immunity in a country with a historically high level of DTP3 coverage. Whilst widespread immunity is seen in infants and young children, there is evidence of reduced immunity in populations not targeted by EPI and MNT programmes. As protection against tetanus is only achieved through individual vaccination, efforts should be made to address these immunity gaps by strengthening and expanding existing programmes, as well as education about post-exposure prophylaxis.

## 6 Contributions

CLT, MB conceived the study. TTT, JC, LVT, MFB designed the study. Data acquisition was performed by NTHN, LAN, NTDN, CTT, TLT, NTD, JC. TTT, JC, LVT PQT, NTD supervised the study and provided logistical support. MFB and MC carried out analysis. CLT, MC, MFB, PQT additional interpretation. CLT, MC MFB, TTT and PQT produced the initial draft of the manuscript. All authors reviewed and approved the final manuscript draft.

## Funding

The study was funded by Wellcome Grant 107367/Z/15/Z and 098511/Z/12/Z. The funders had no part in study design, implementation, analysis or decision to publish.

## Declaration of Competing Interest

The authors declare the following financial interests/personal relationships which may be considered as potential competing interests: Louise Thwaites reports financial support was provided by Wellcome Trust. OUCRU affiliated authors reports financial support was provided by Wellcome Trust.

## Data Availability

Data will be available through the Oxford University Research Archive.

## Appendix A Supplementary material

Supplementary data 1 Positive control OD curves.

Supplementary data 2 Supplementary Tables, Figures and description of ELISA methodology.
