# Supplementary figures and images for: Seroprotection against tetanus in southern Vietnam
Source: Vaccine. 2023 Mar 24;41(13):2208–13. doi: 10.1016/j.vaccine.2023.02.036 (PMC10580288; doi:10.1016/j.vaccine.2023.02.036)

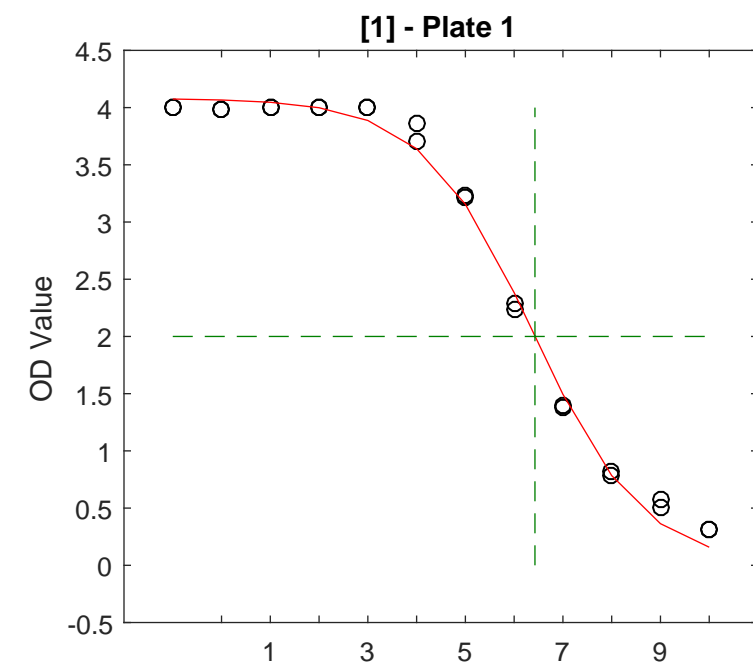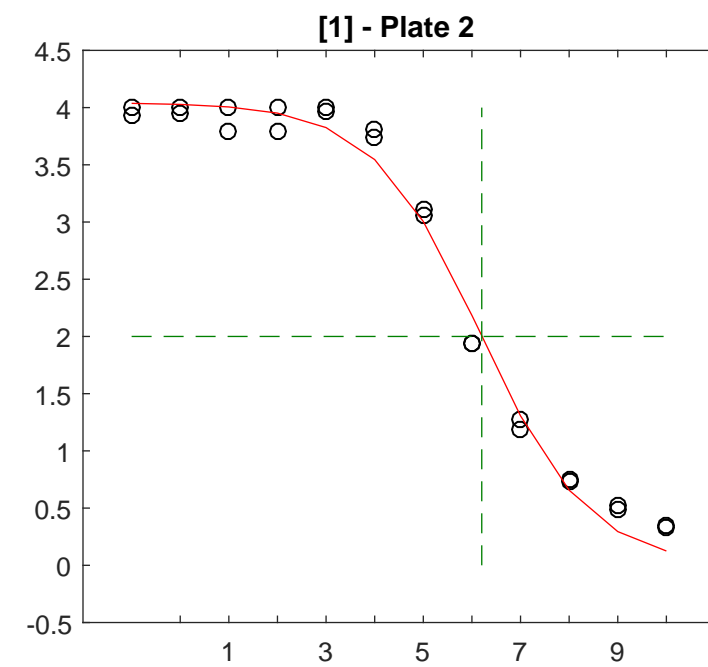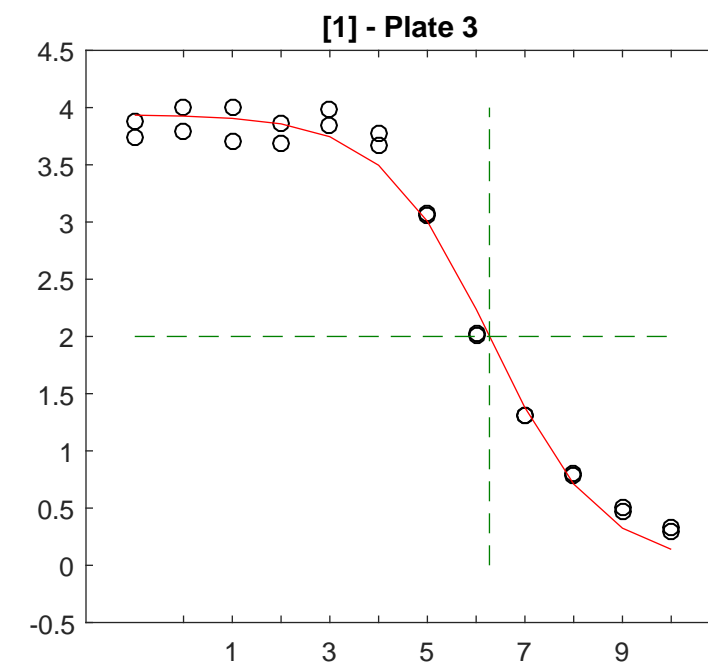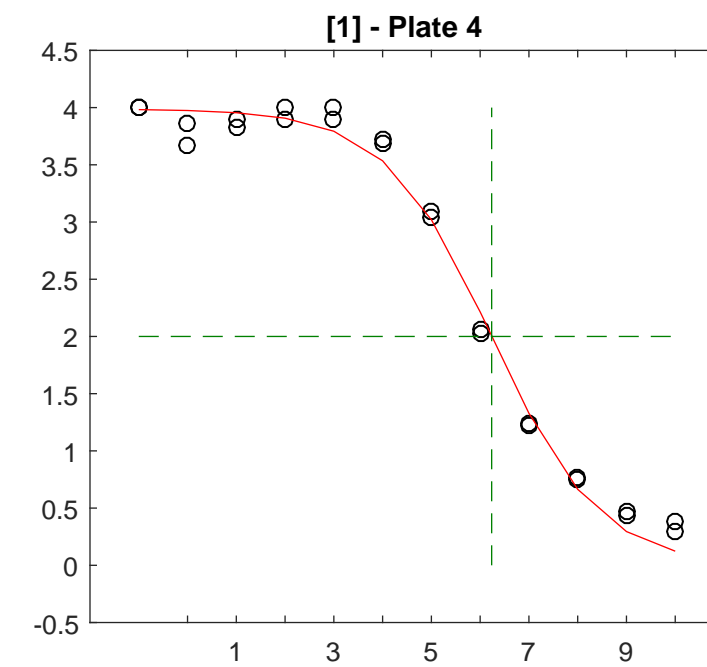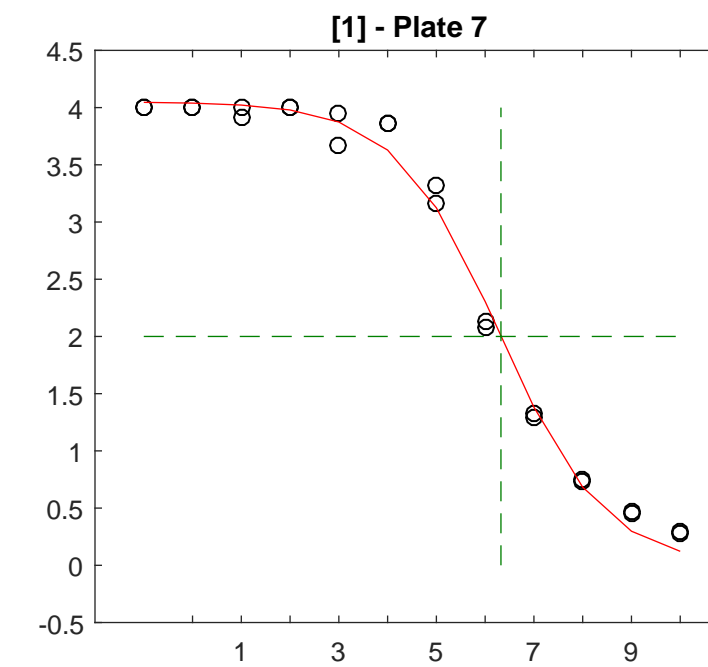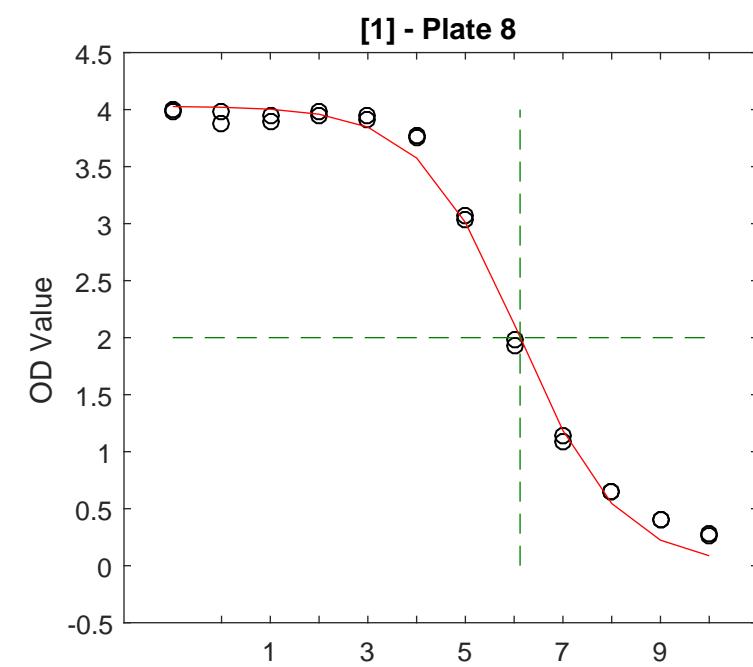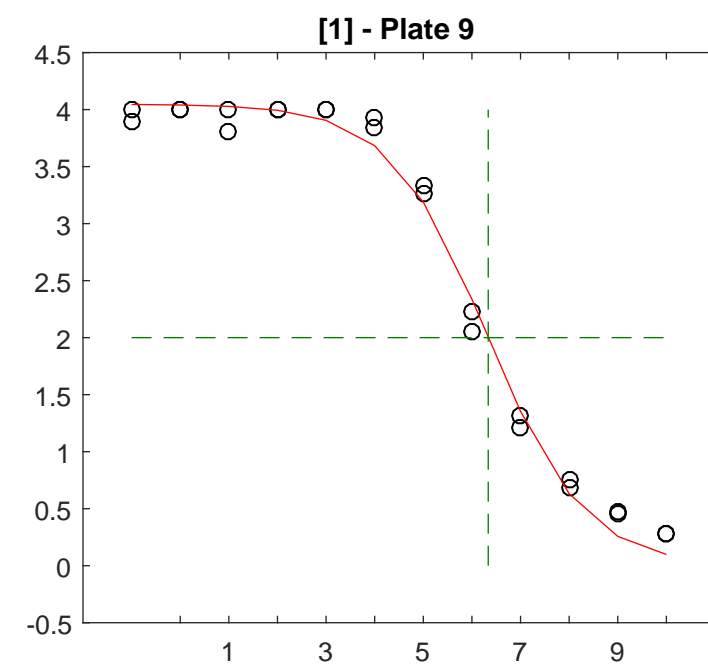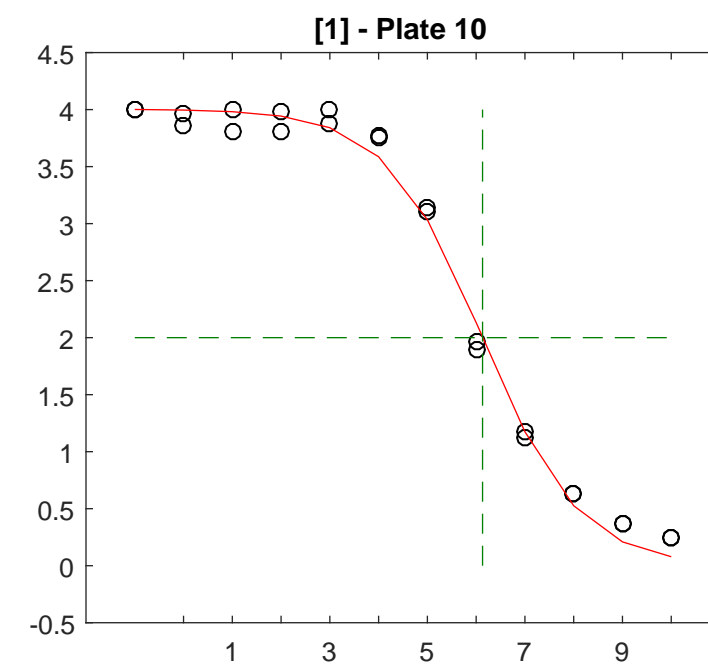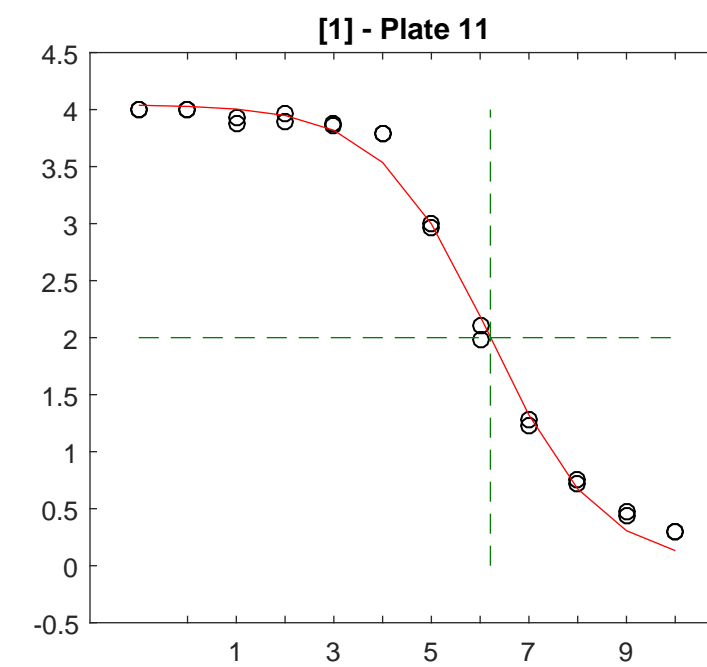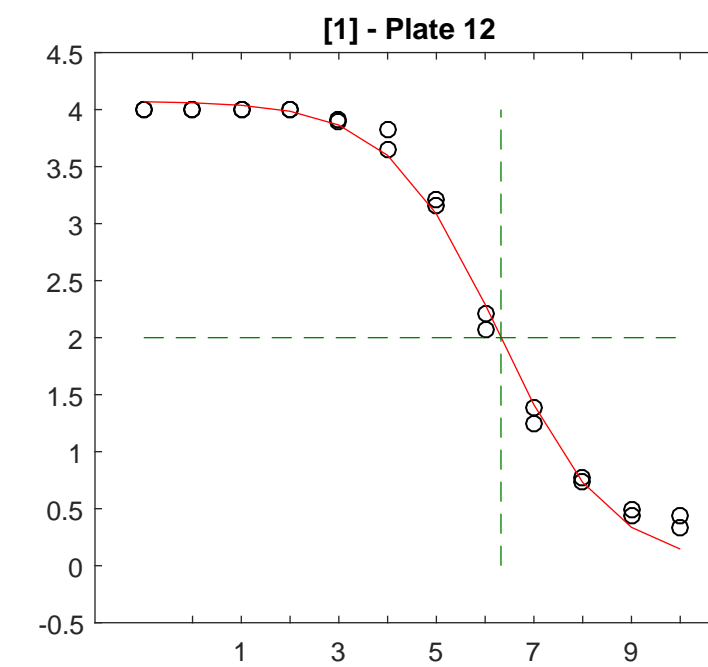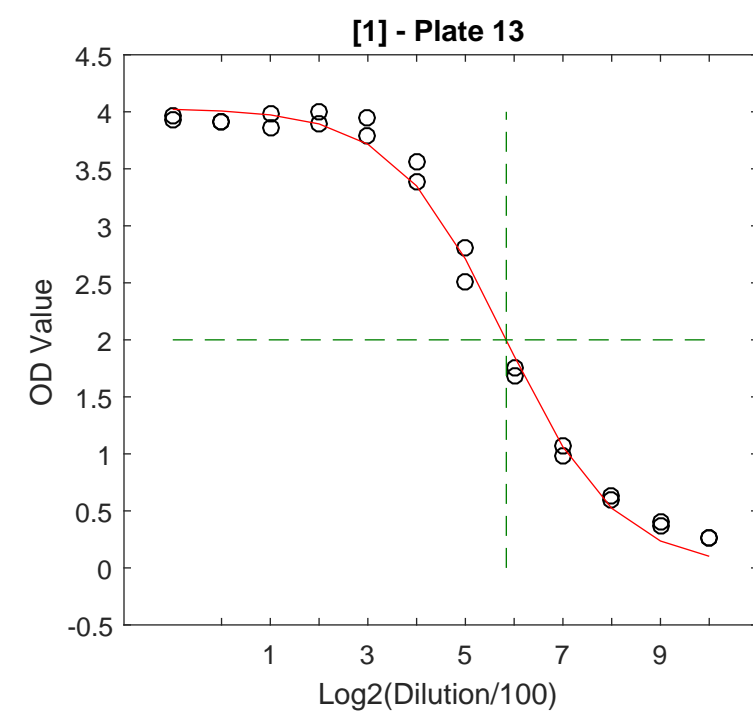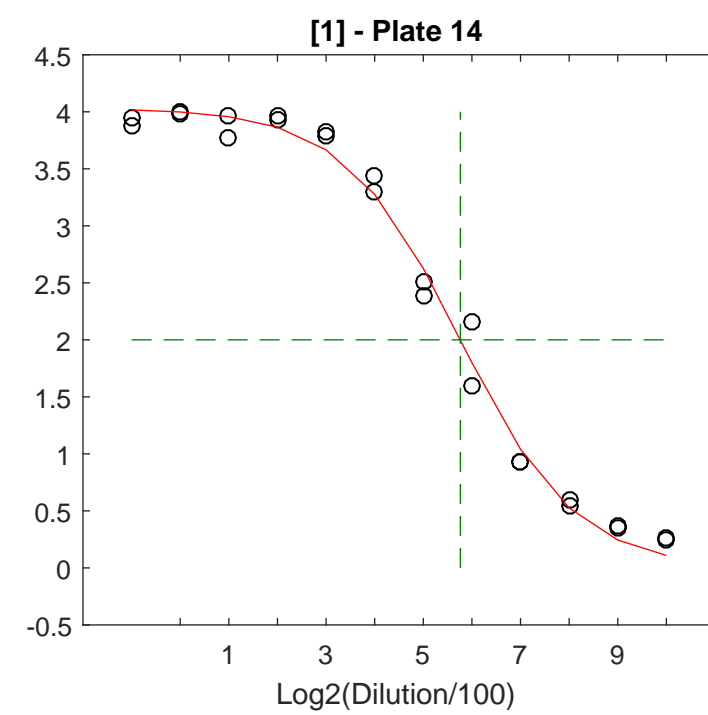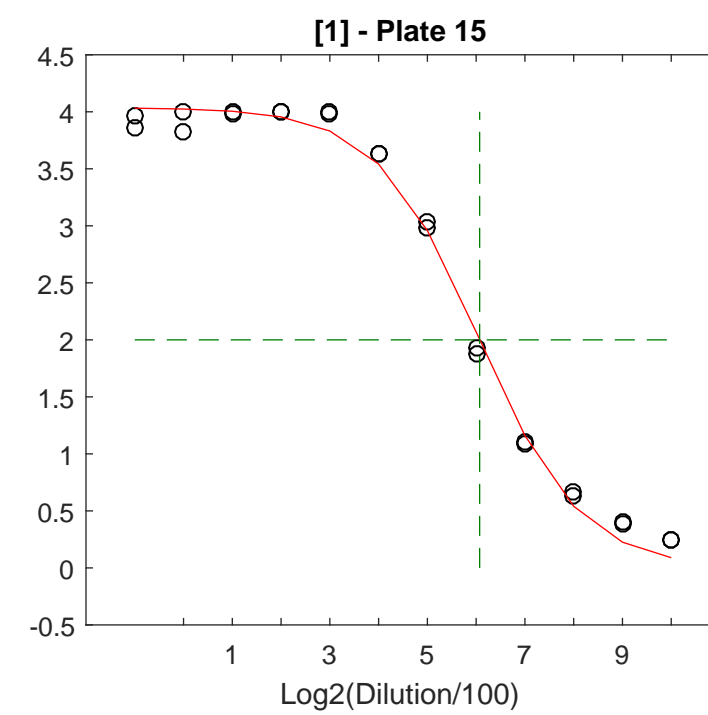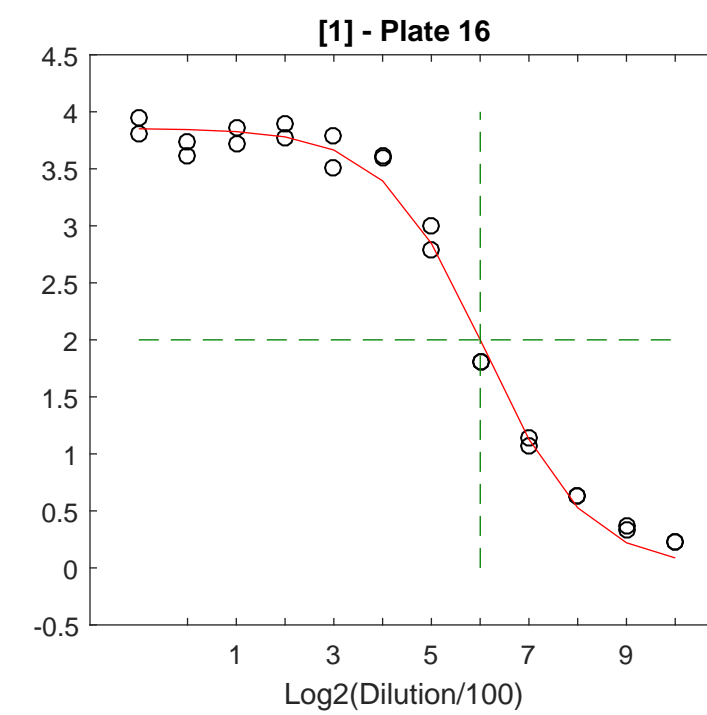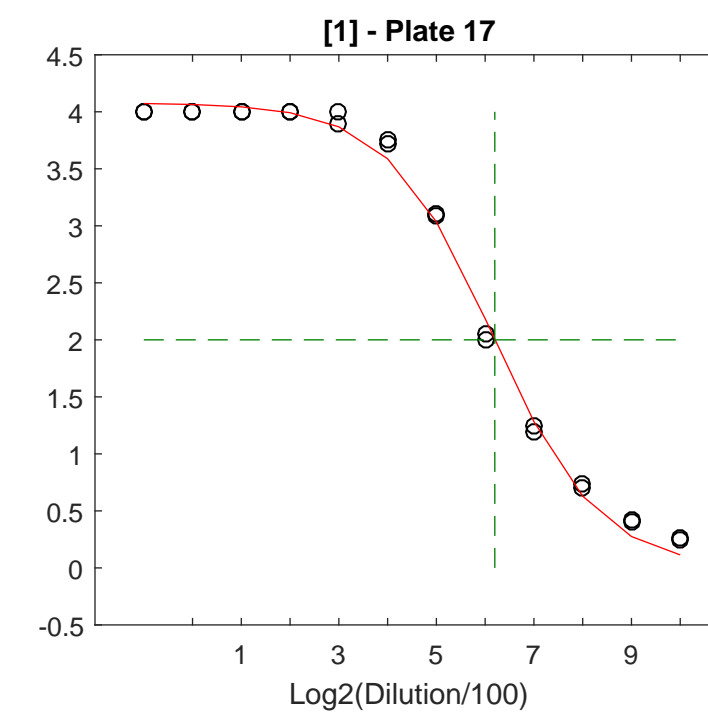

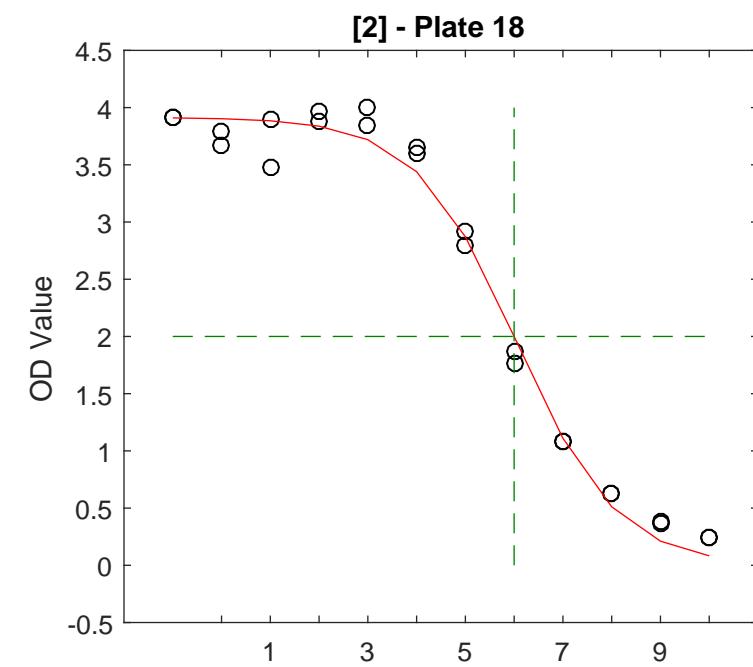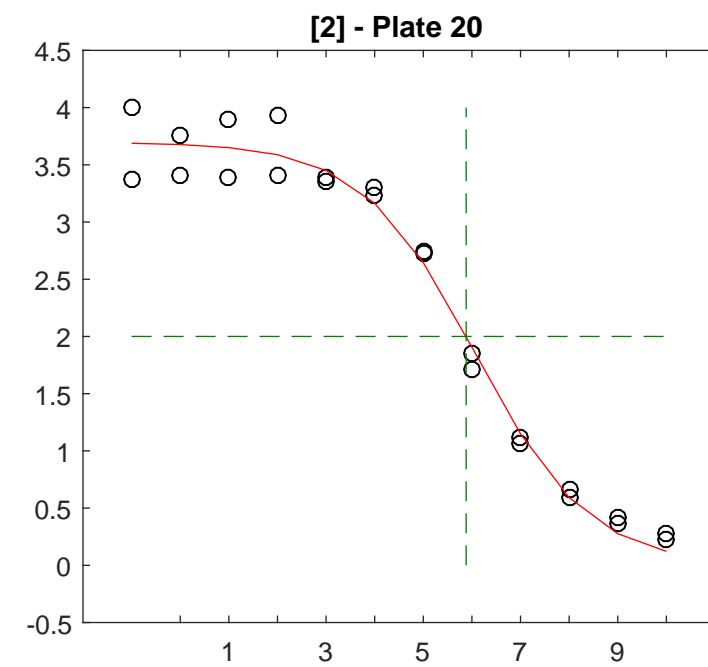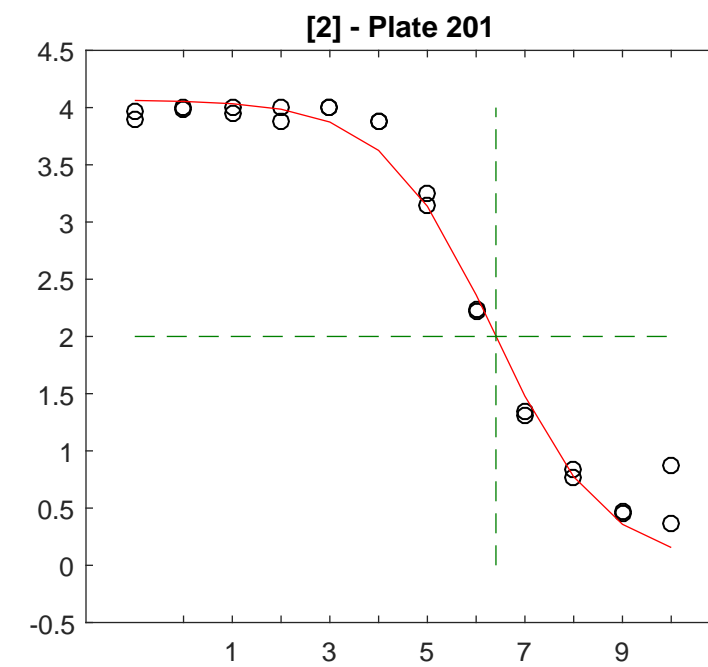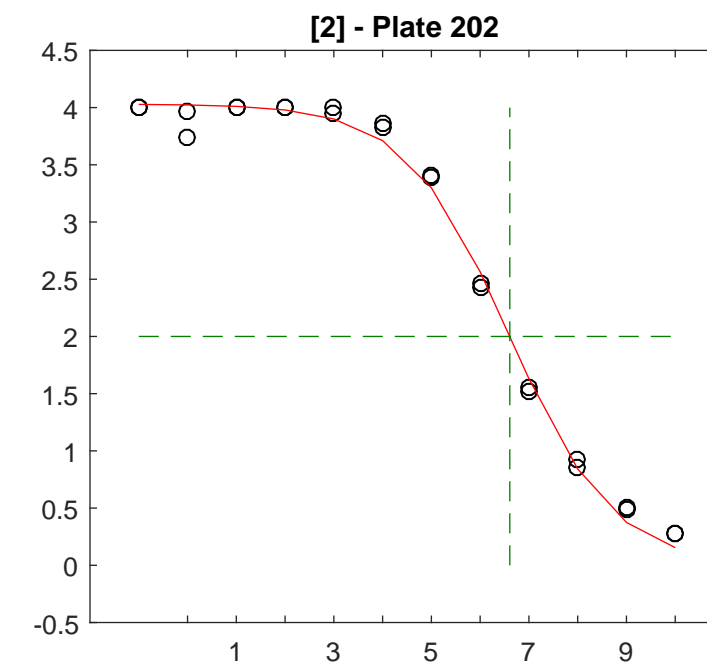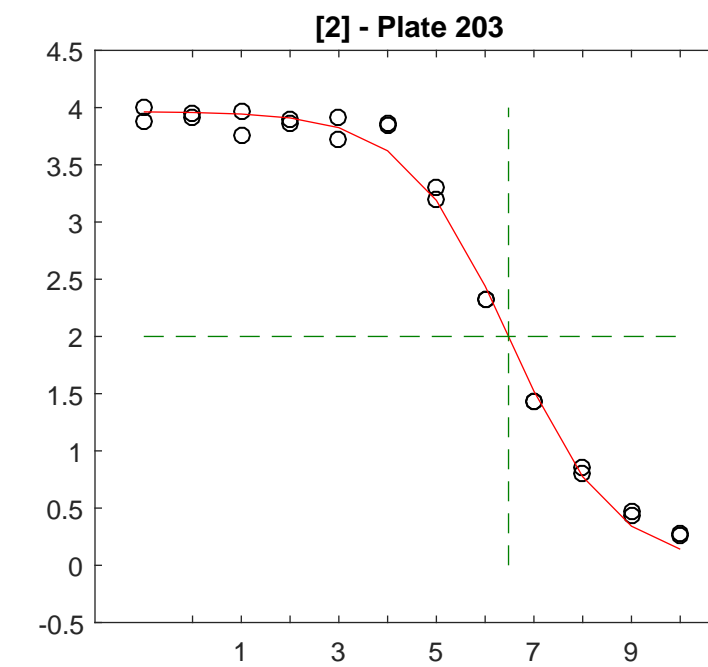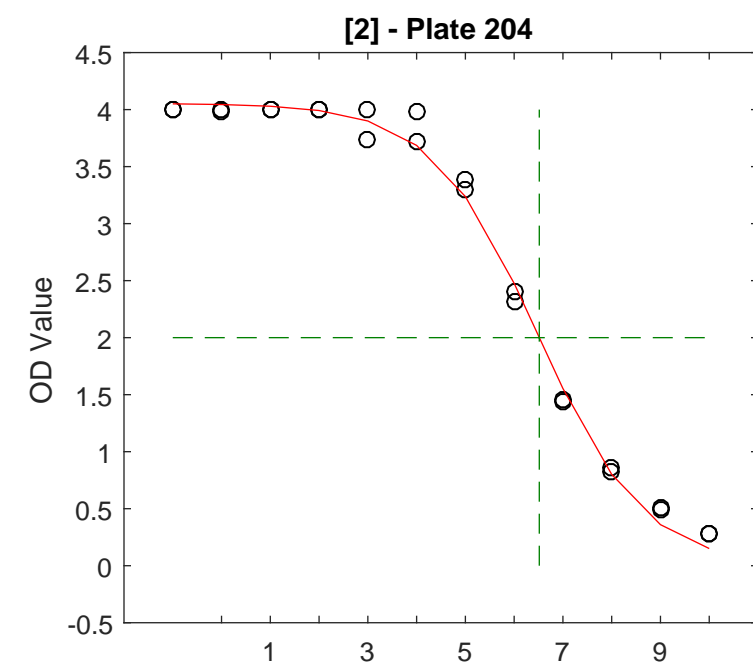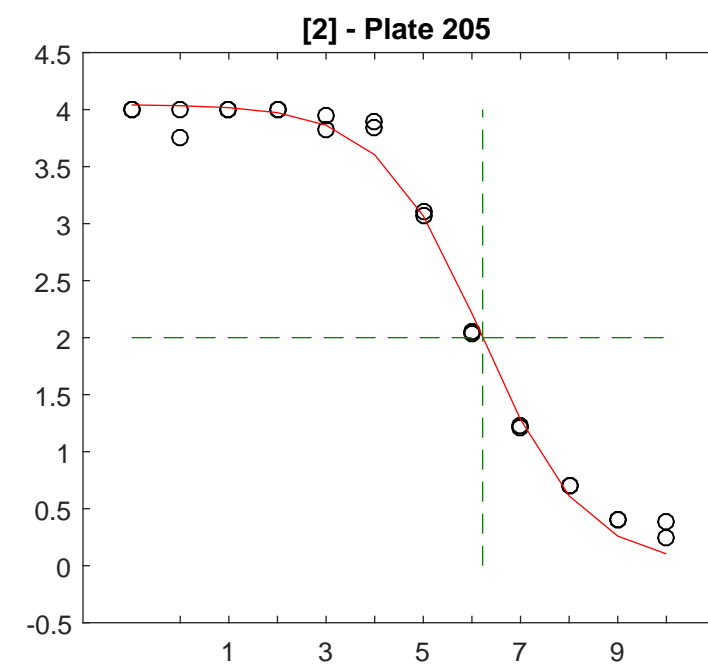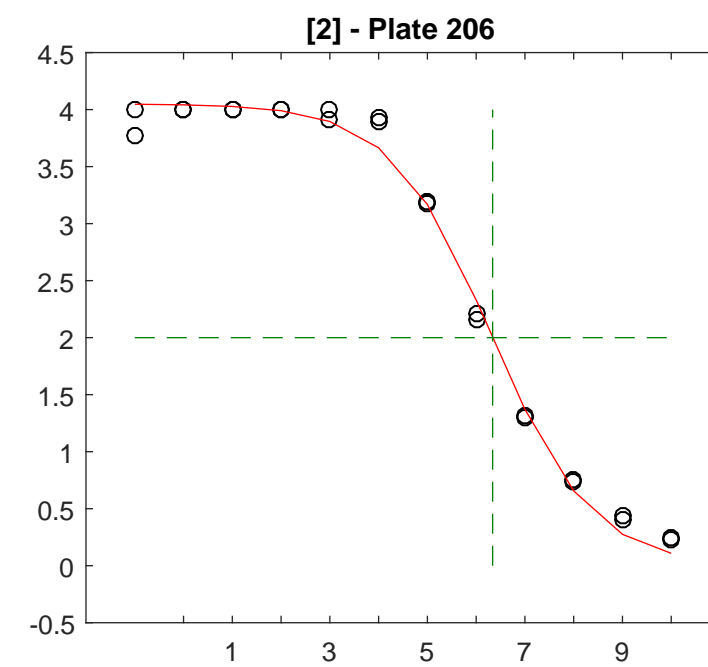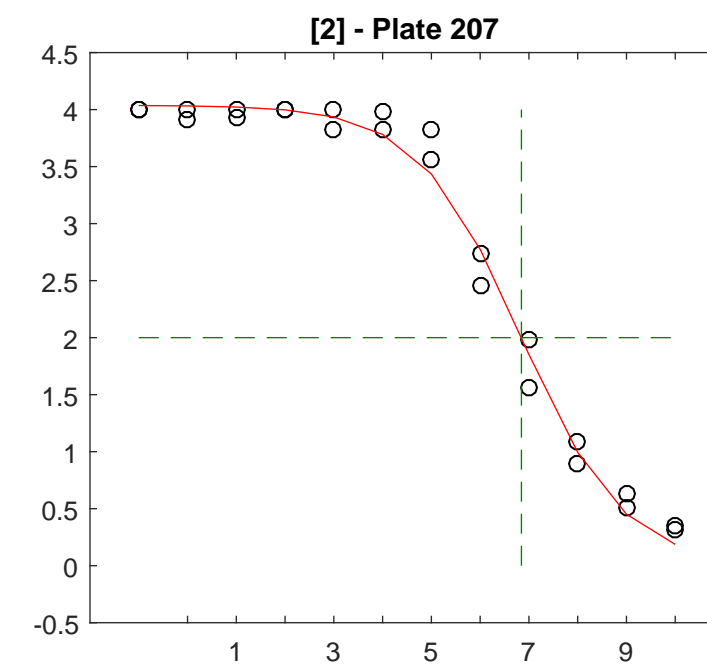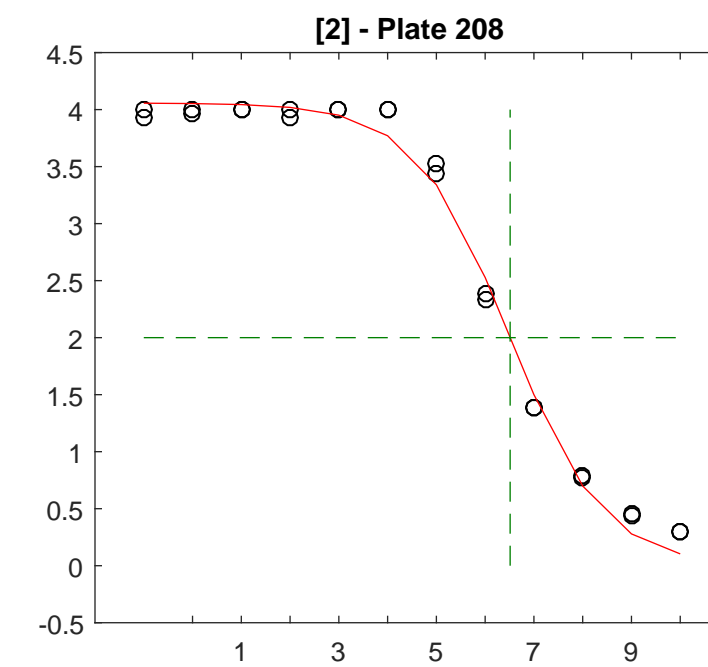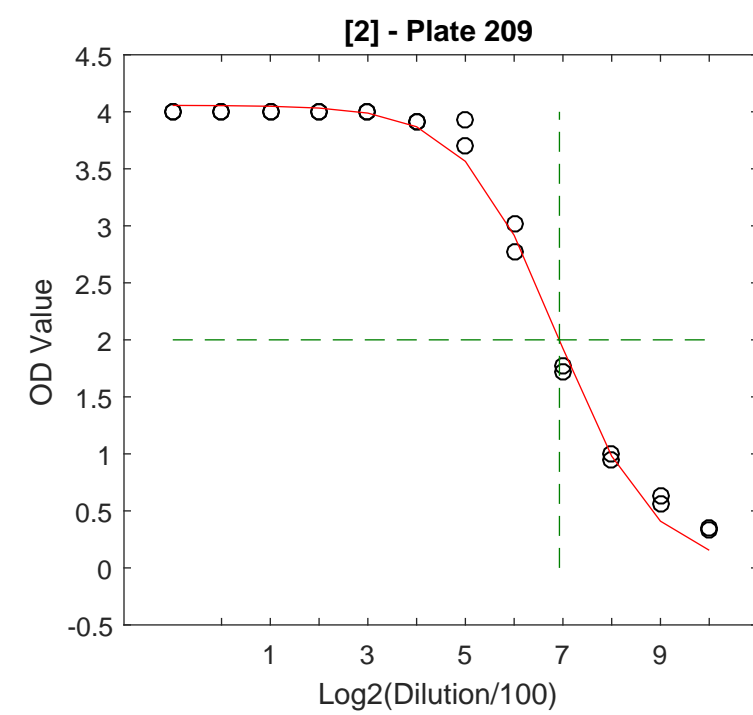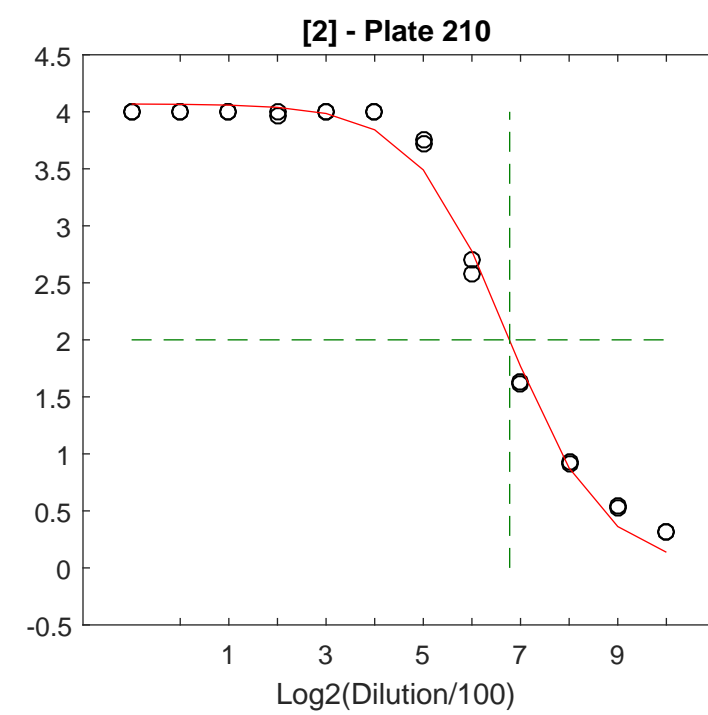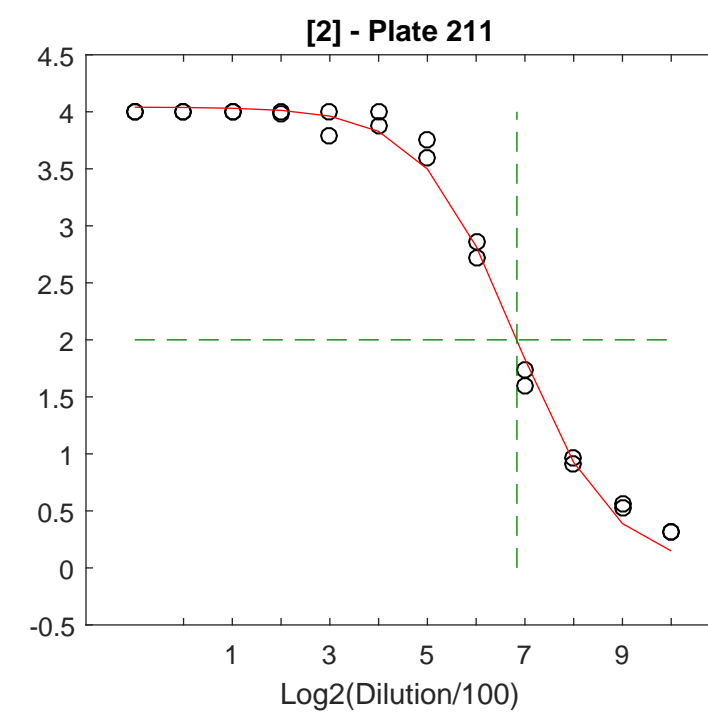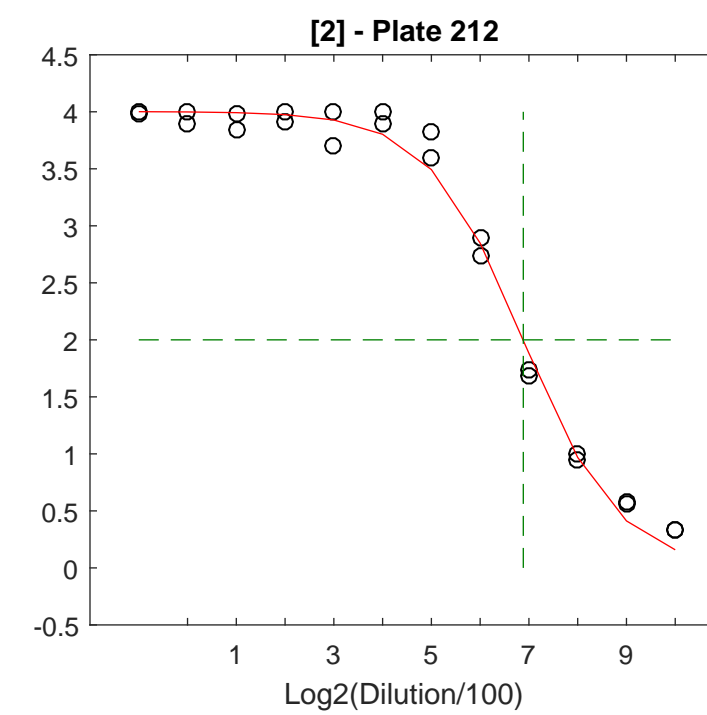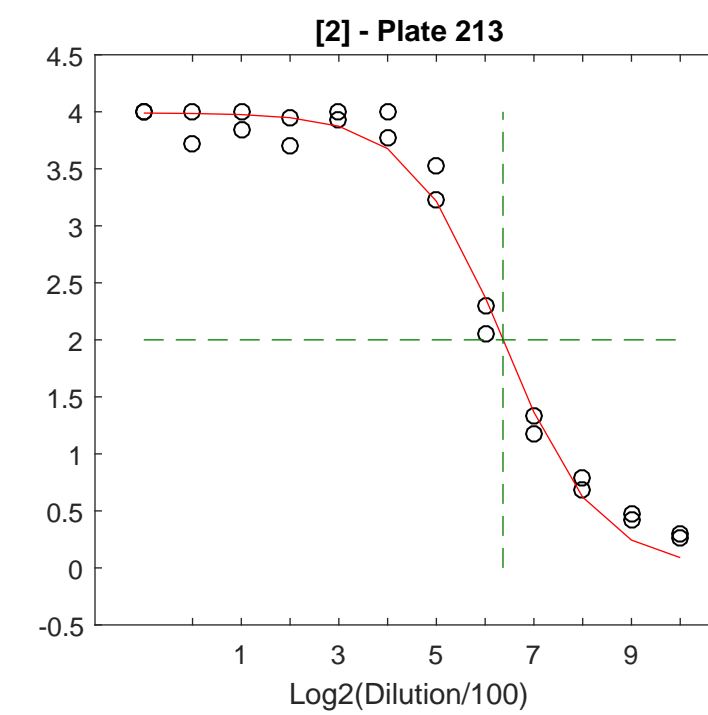

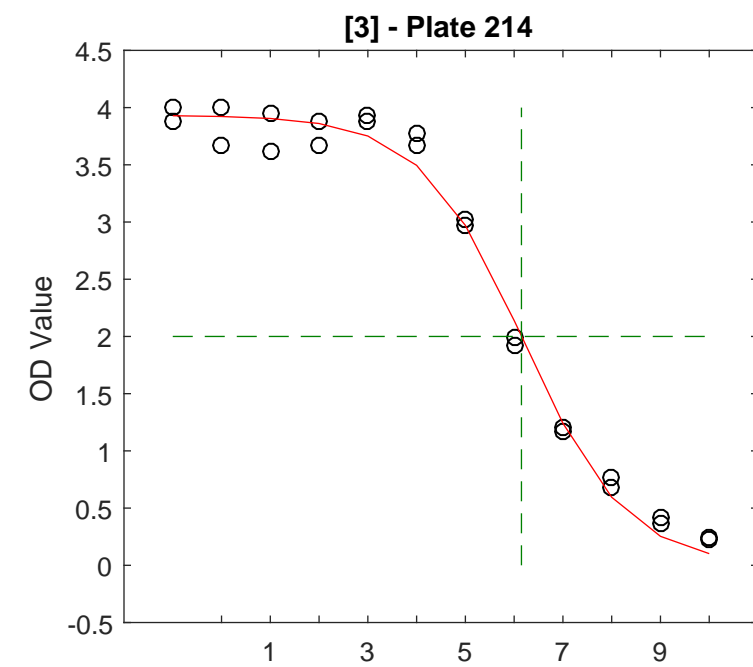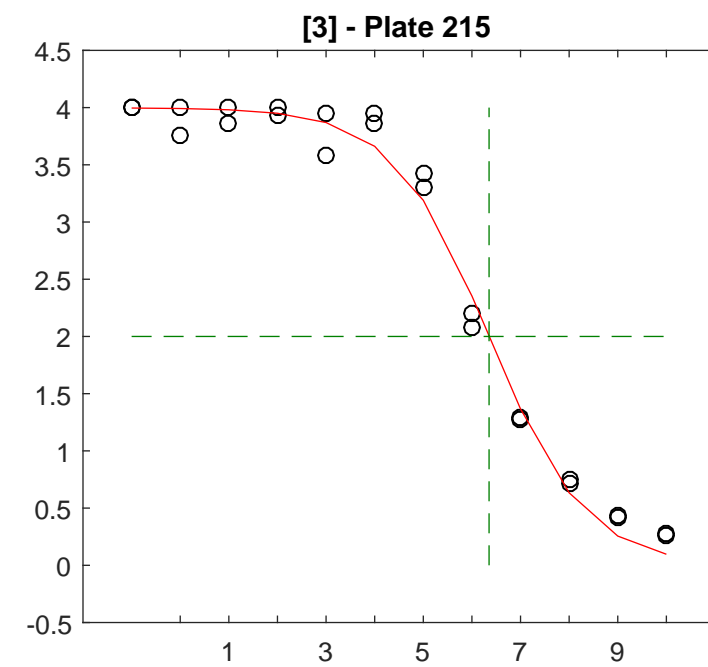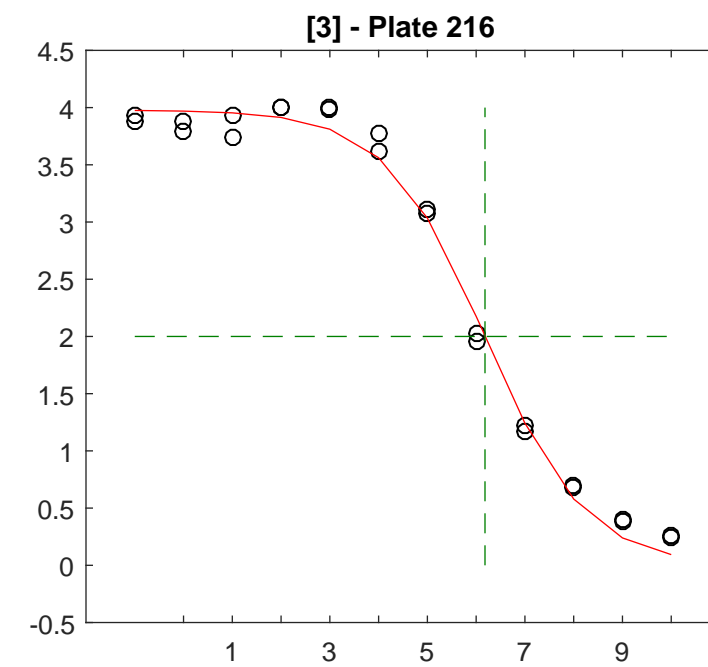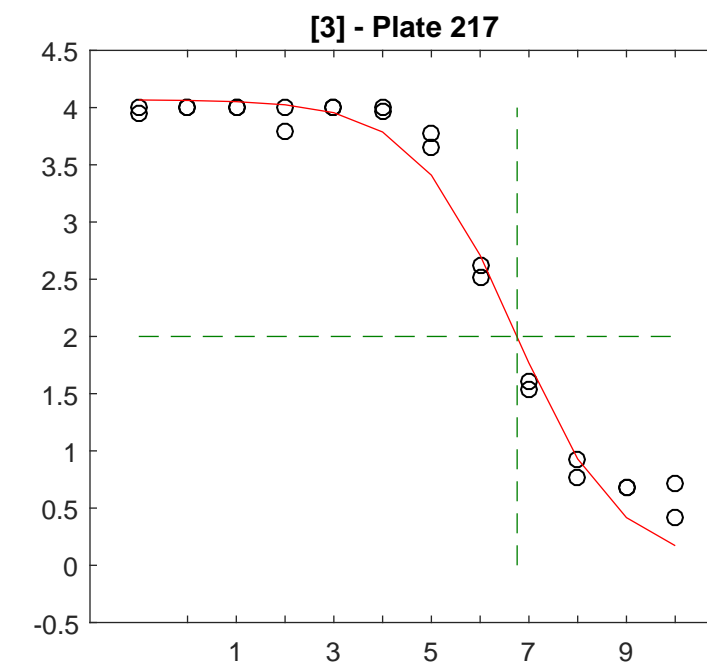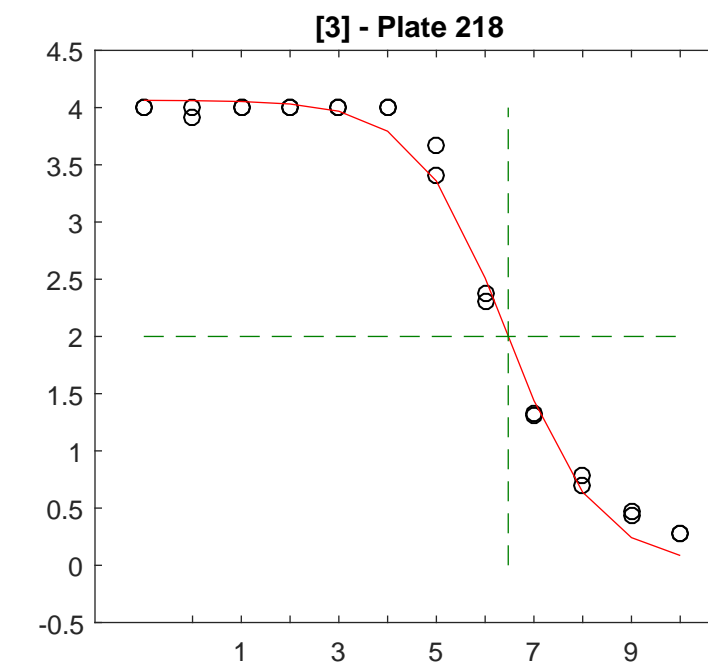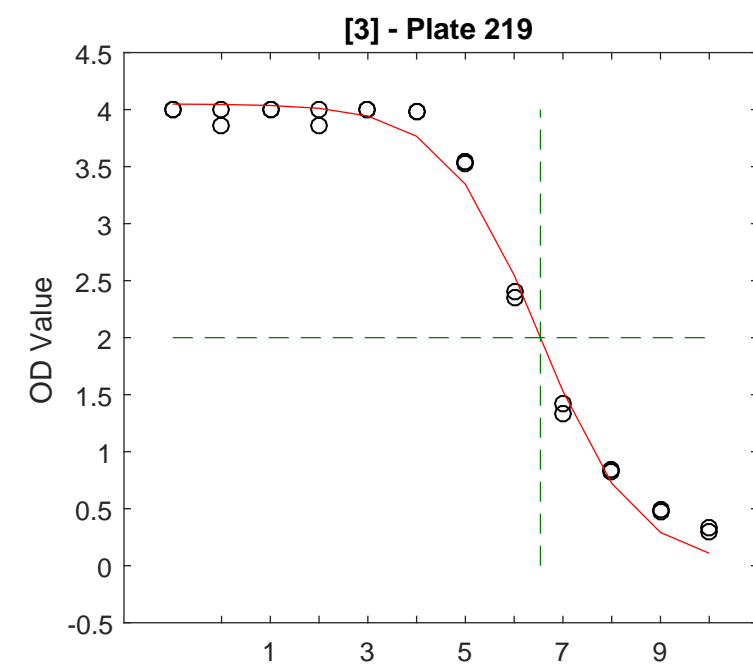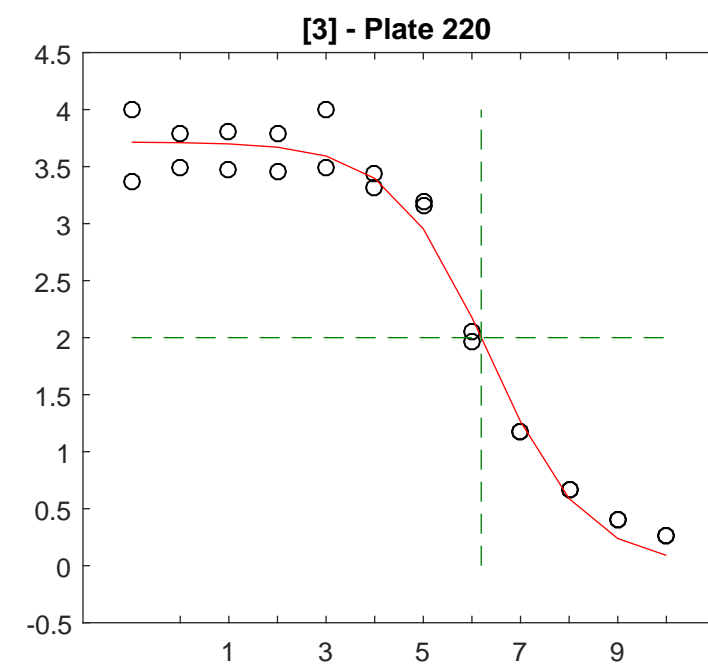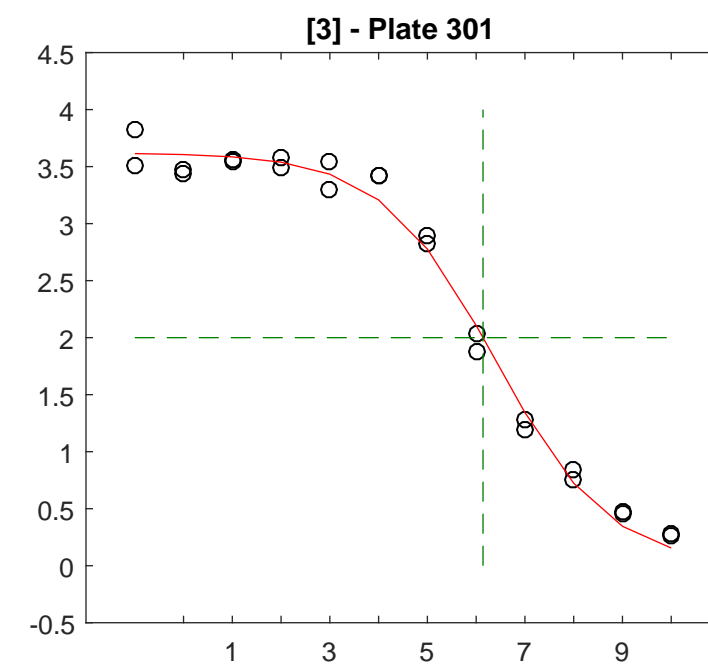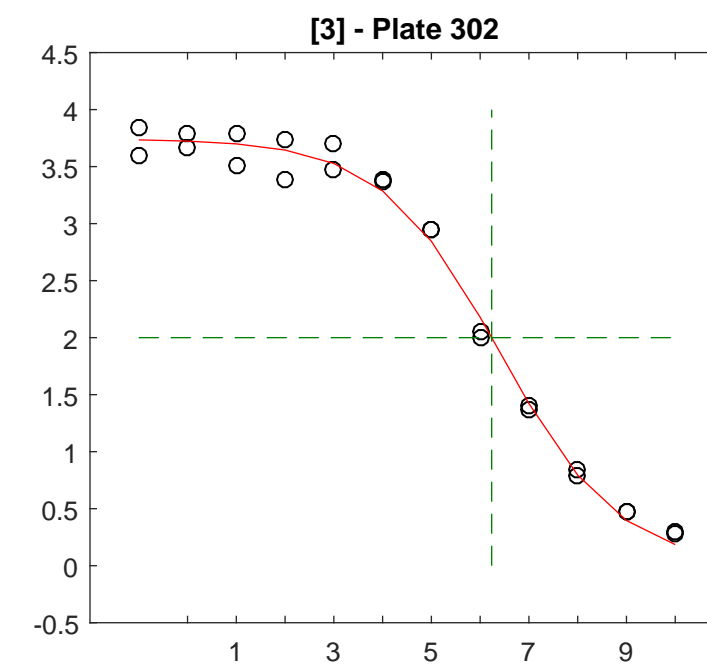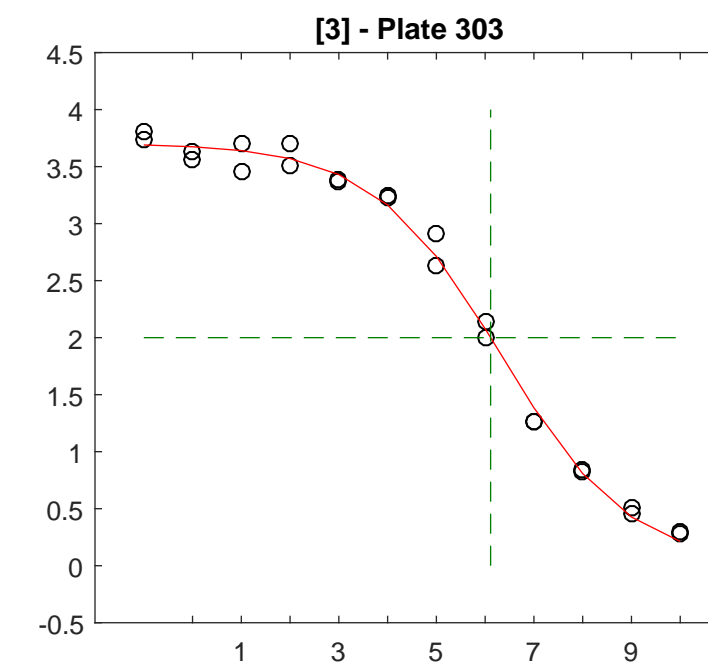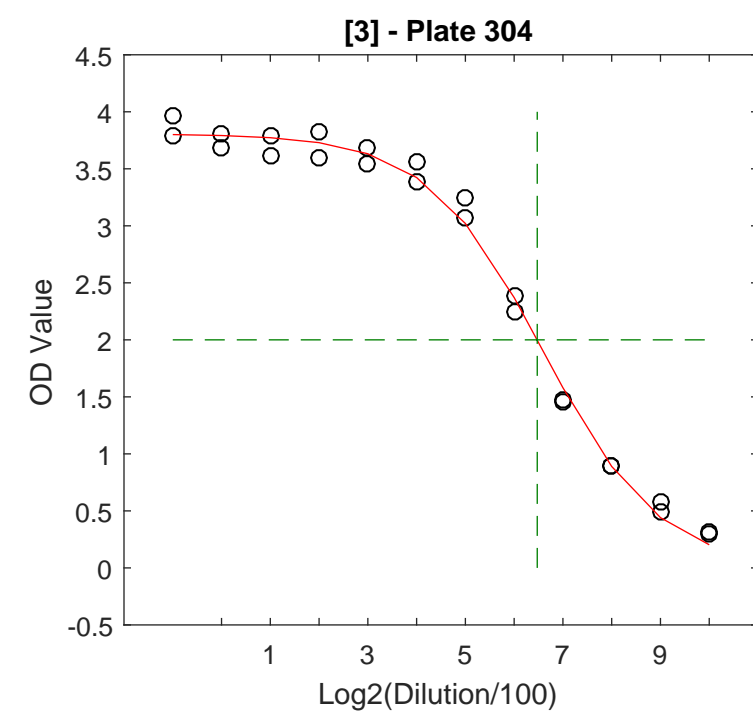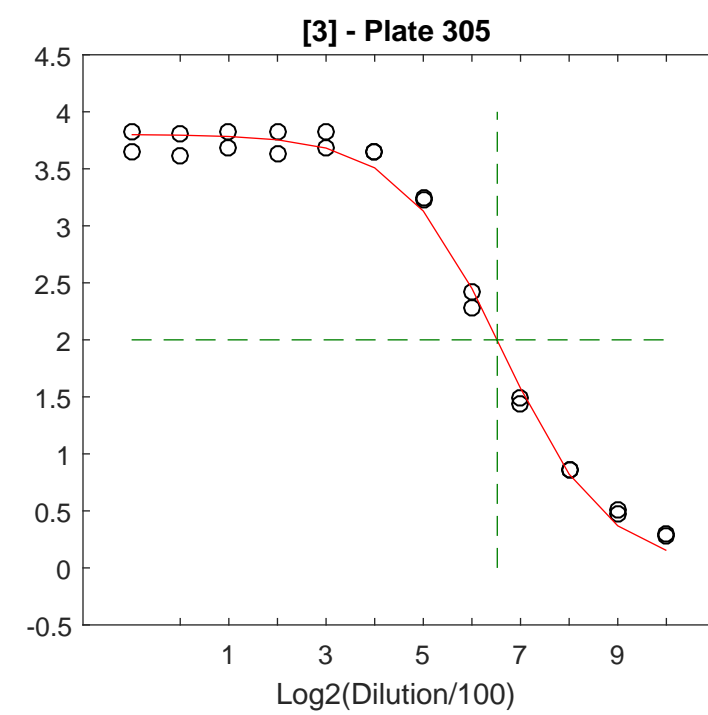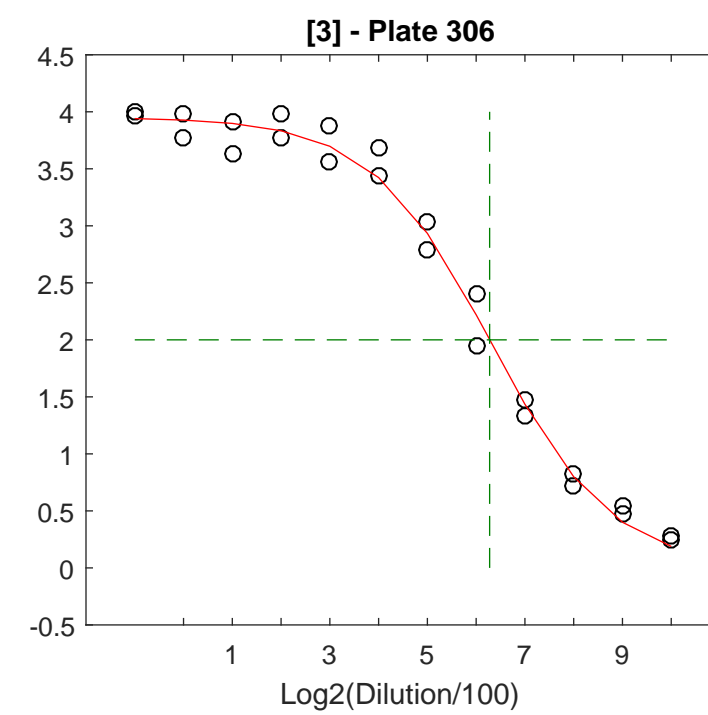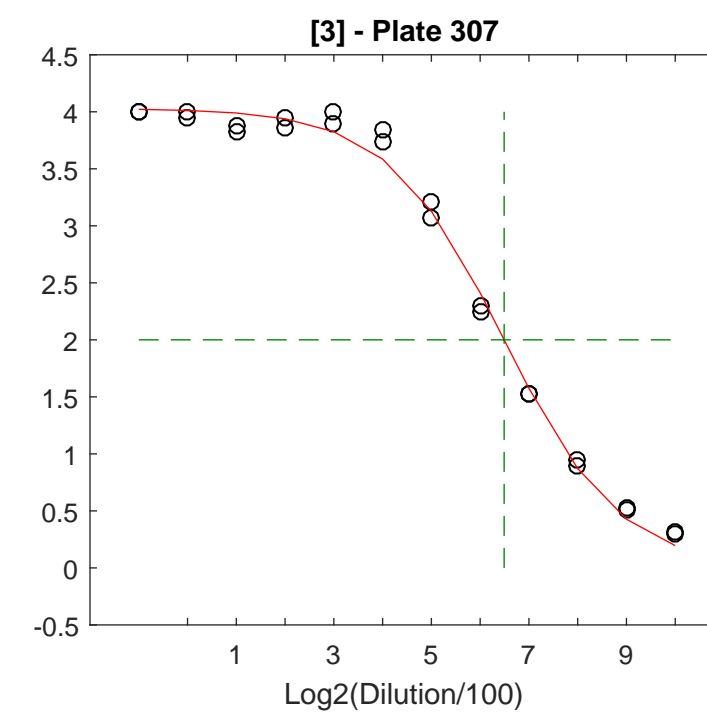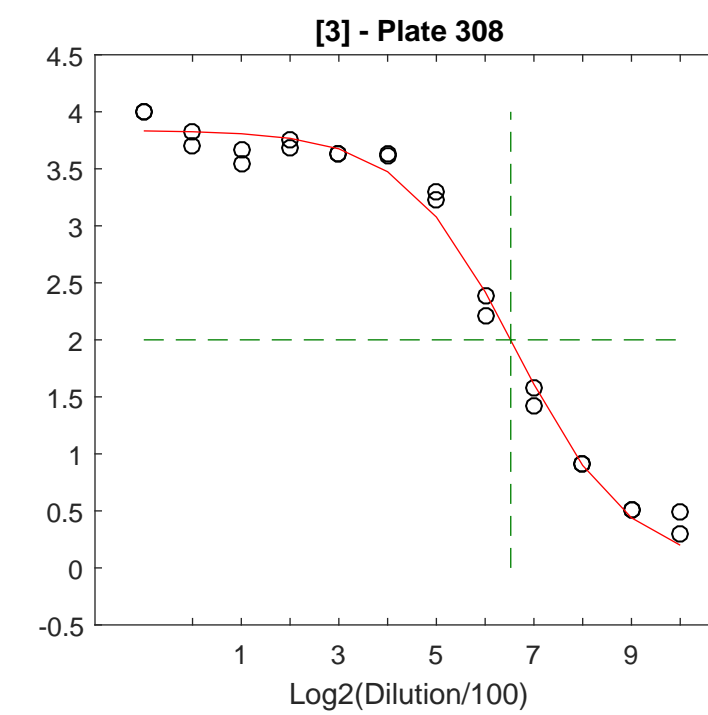

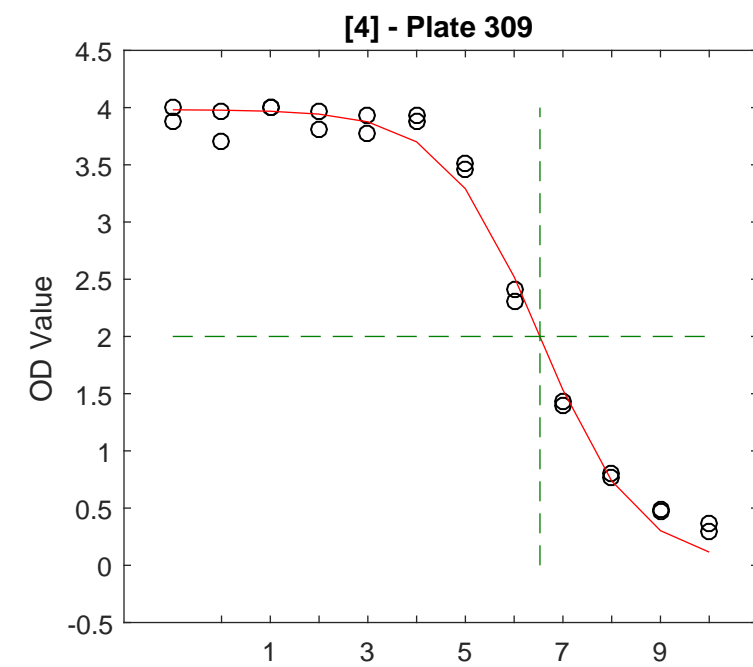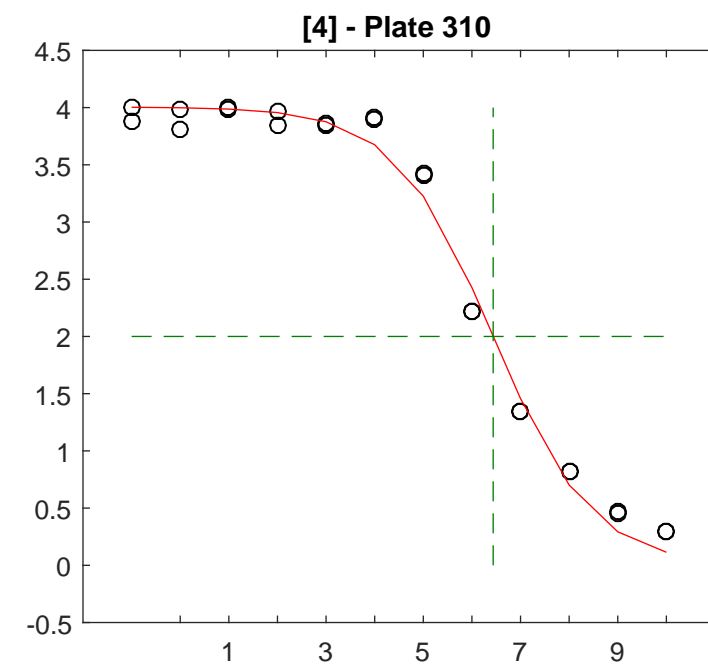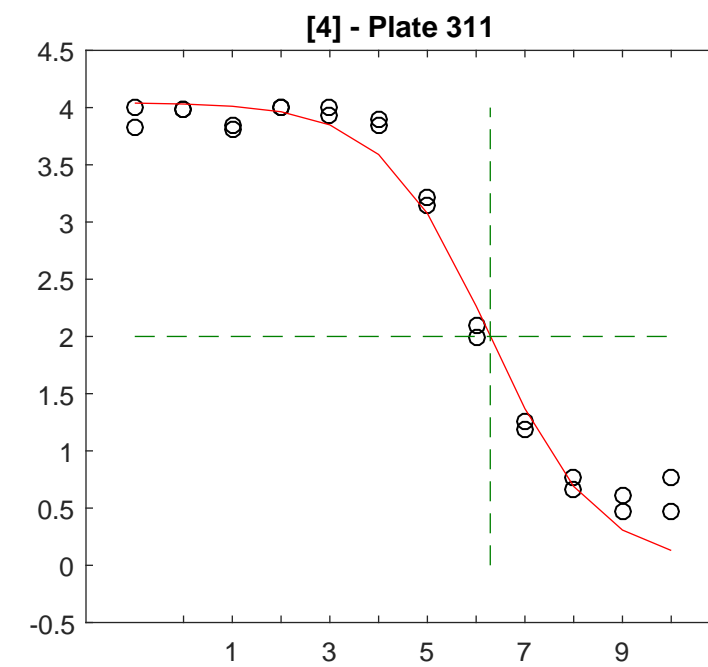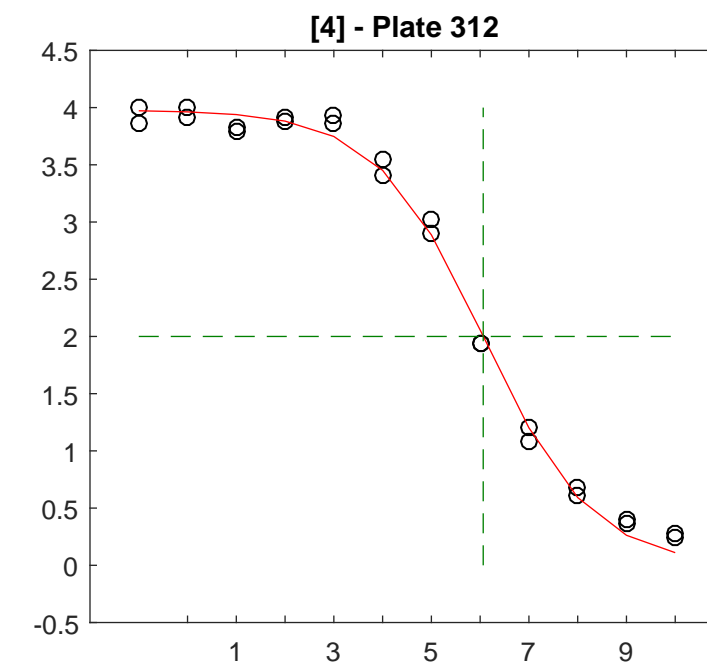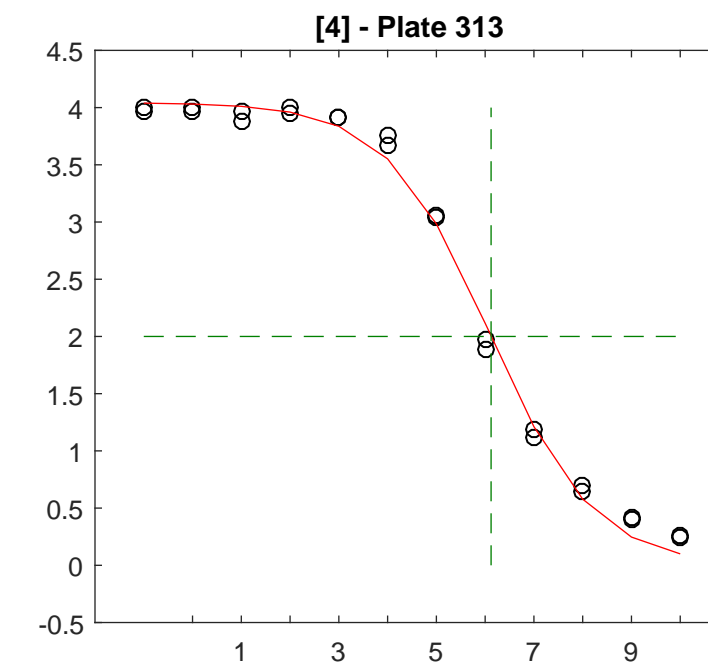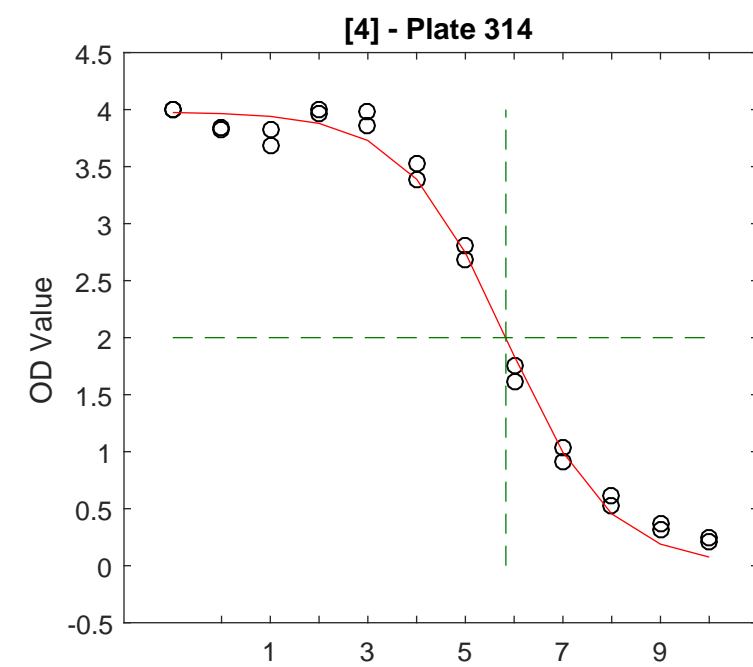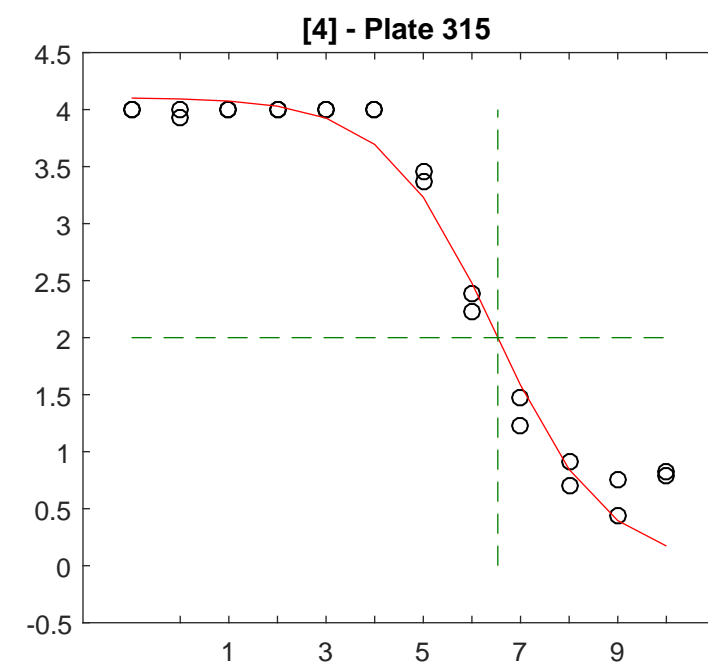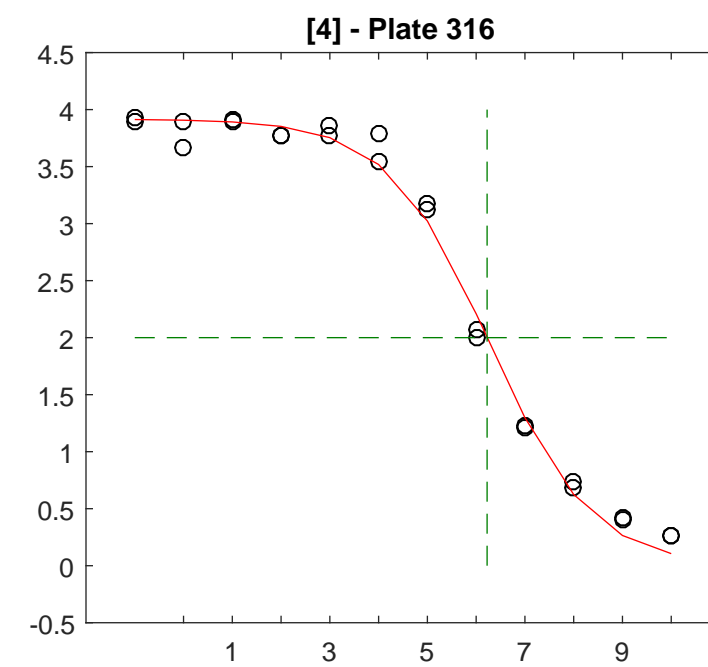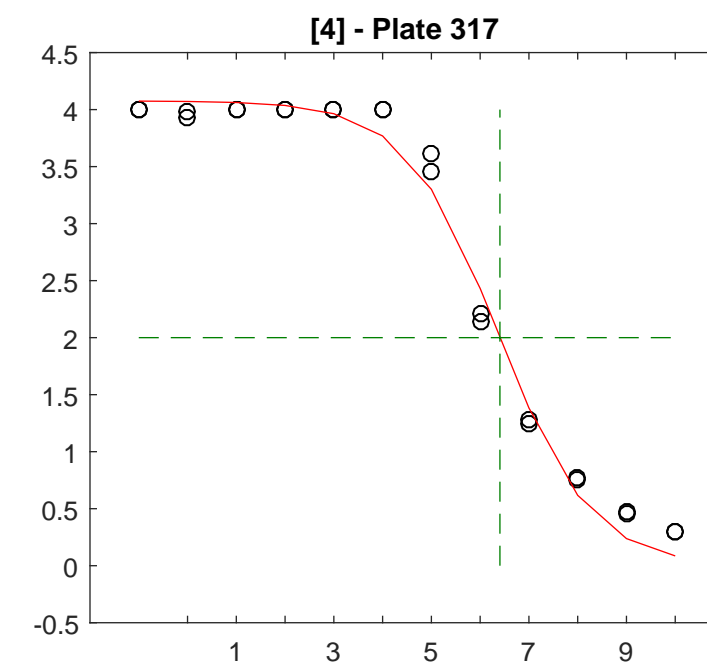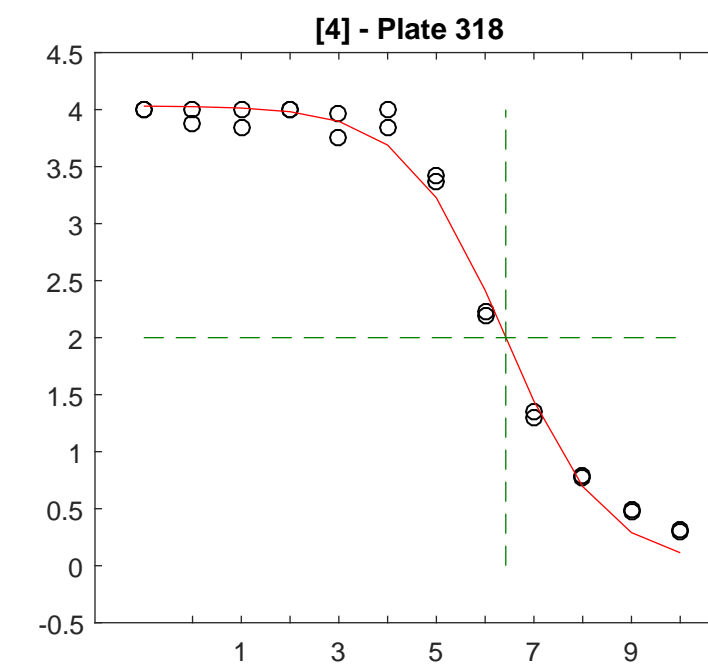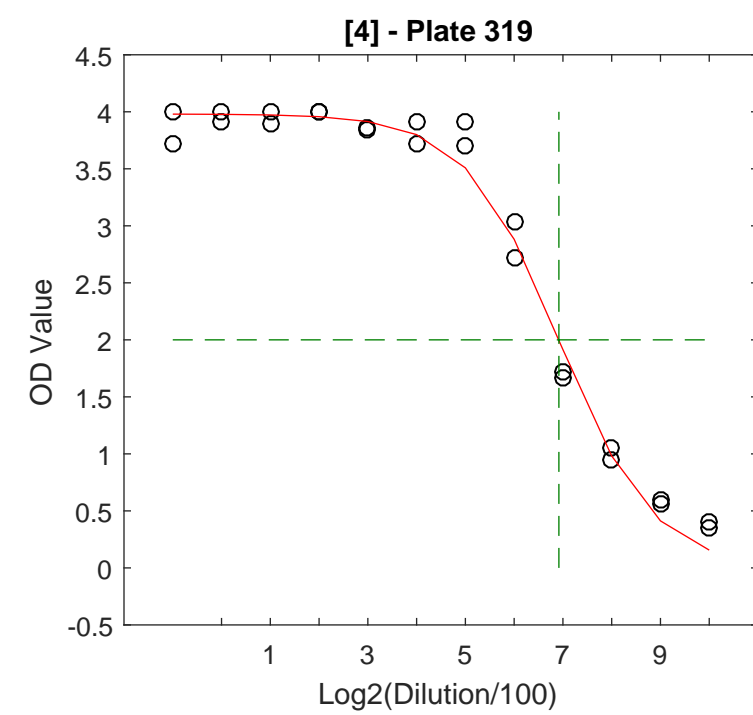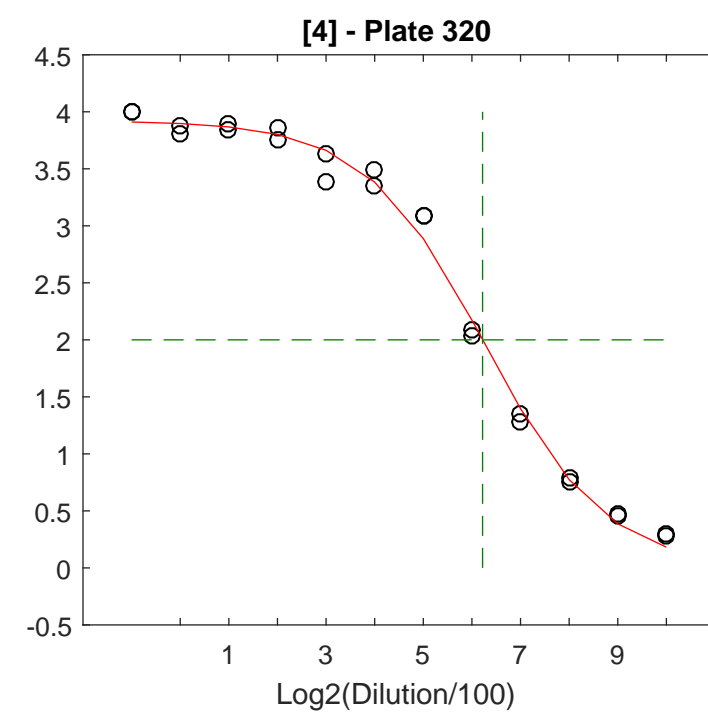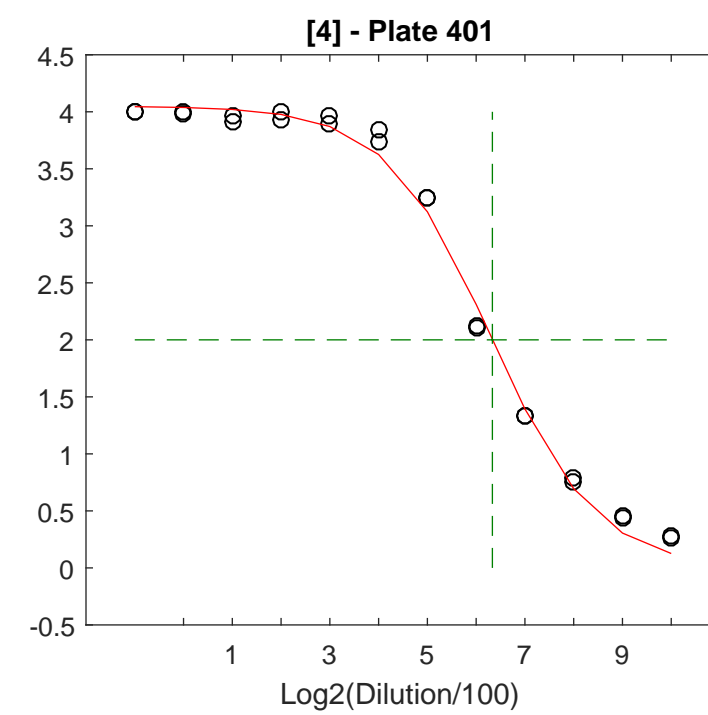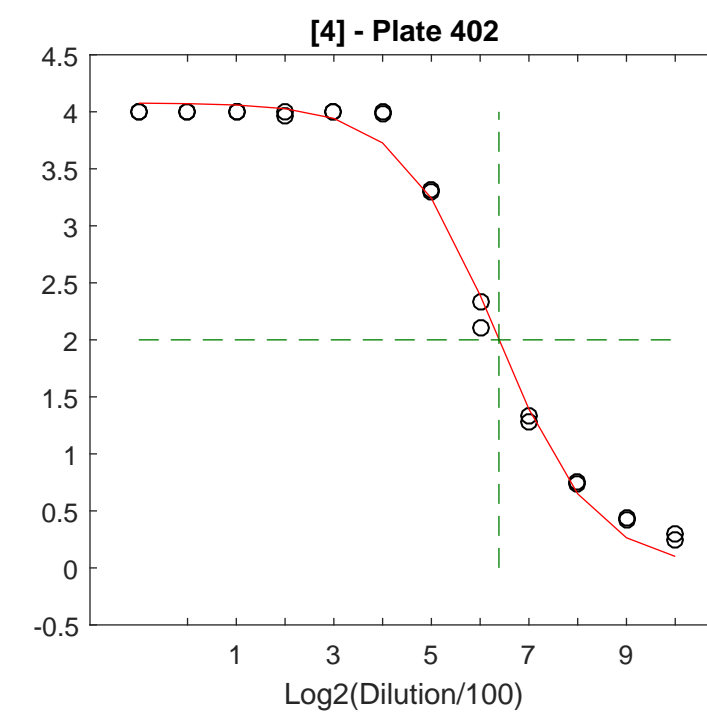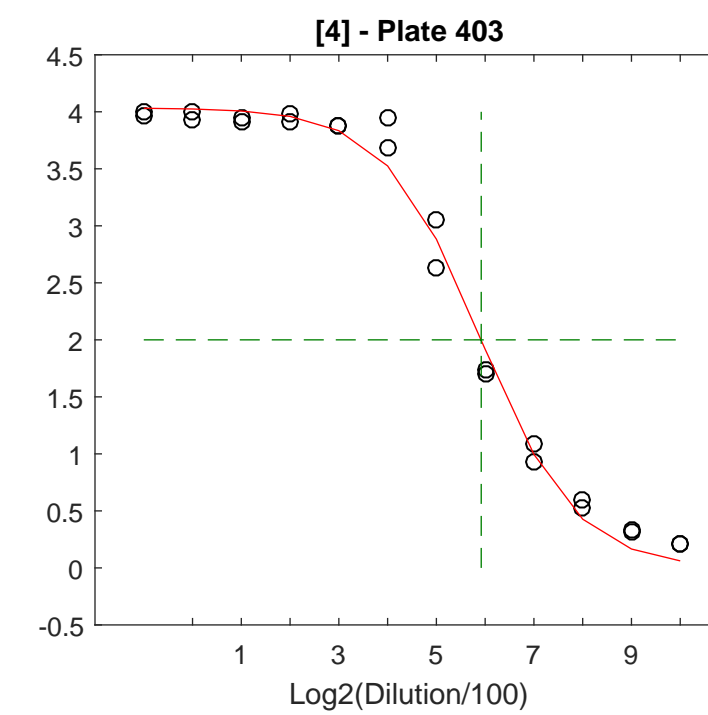

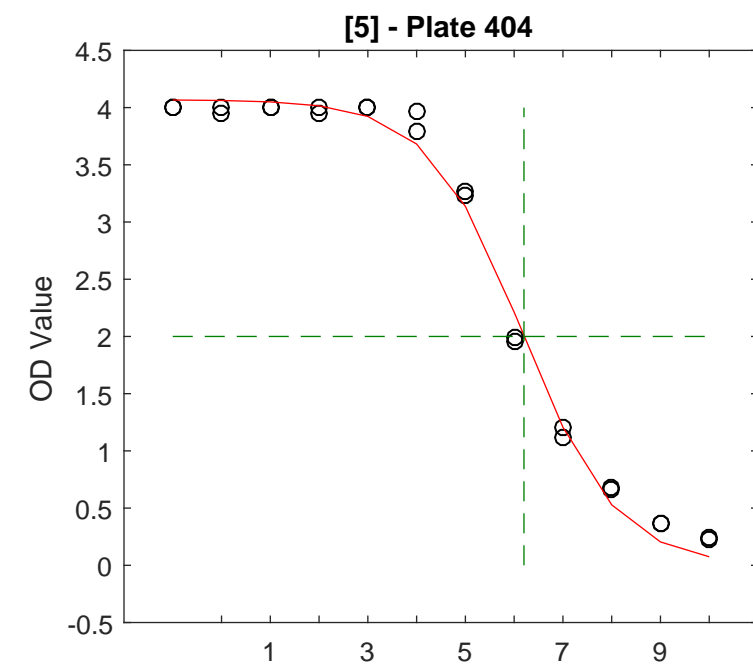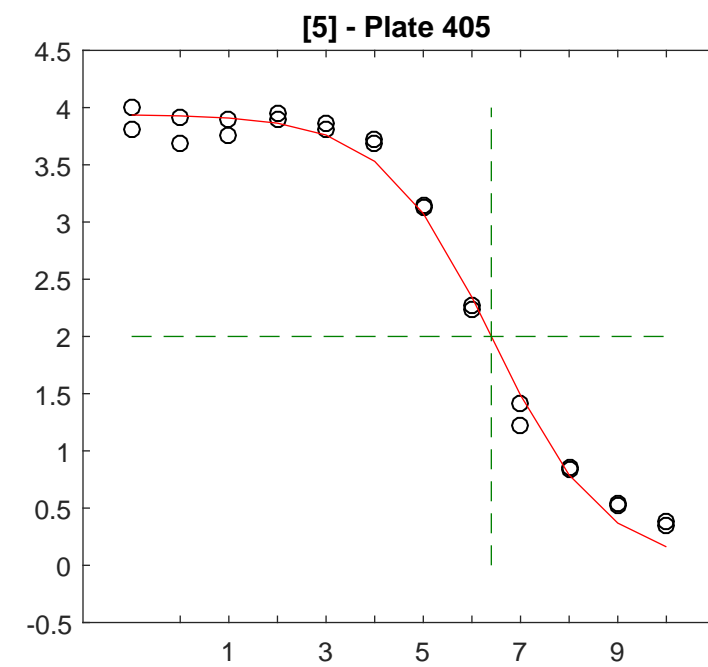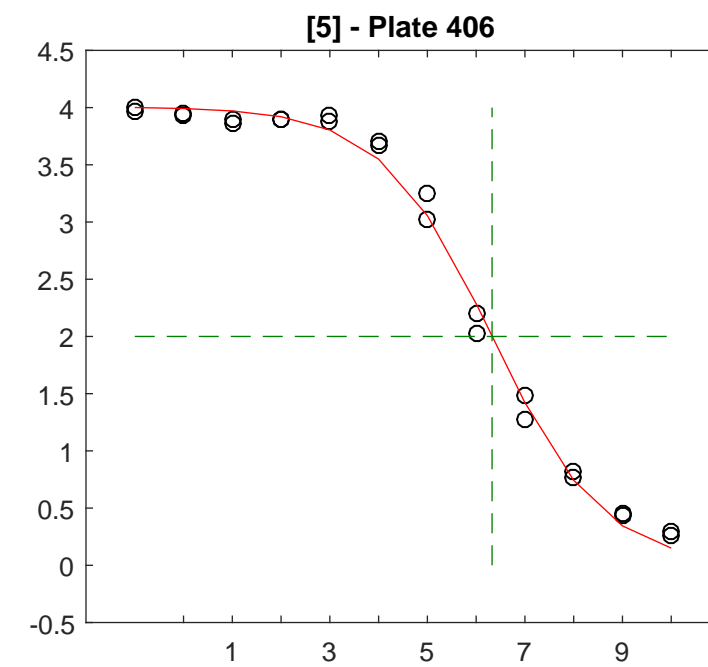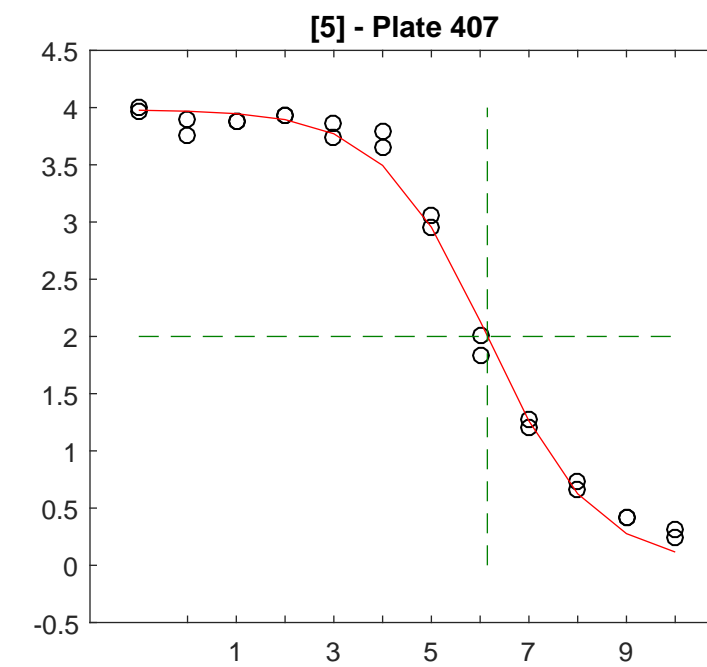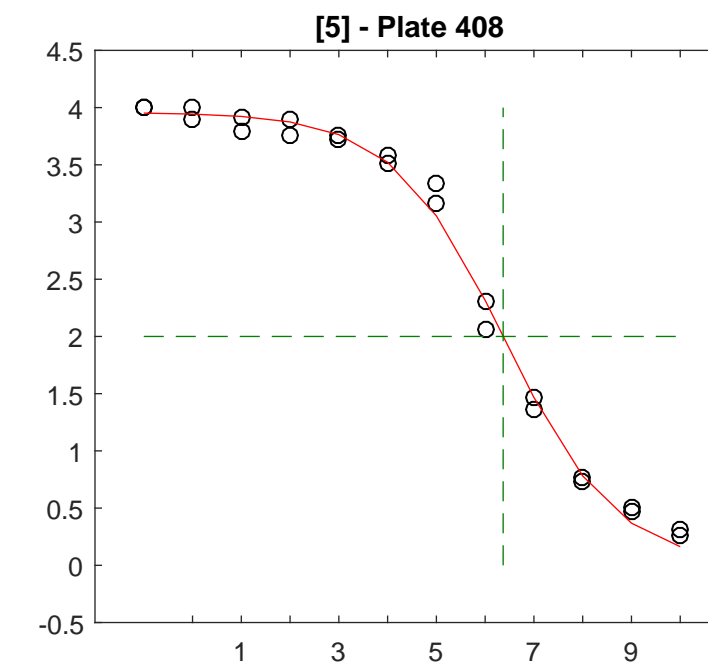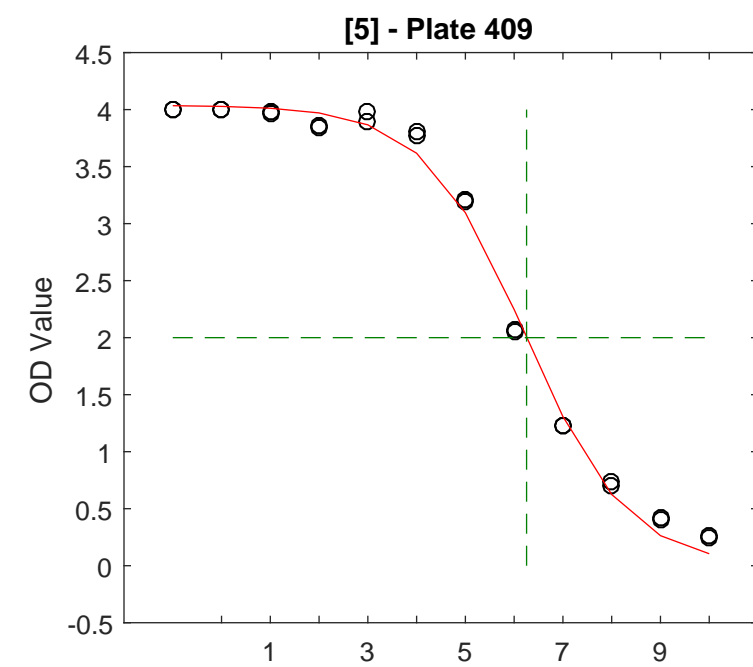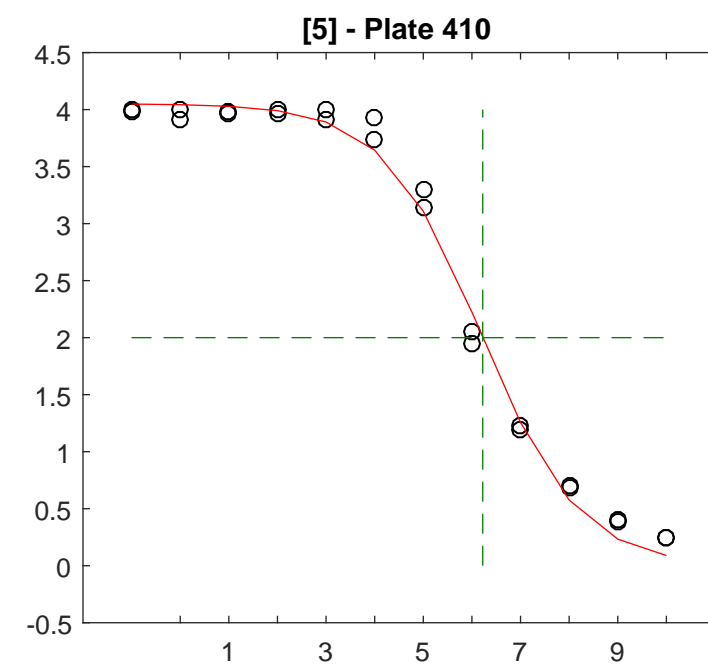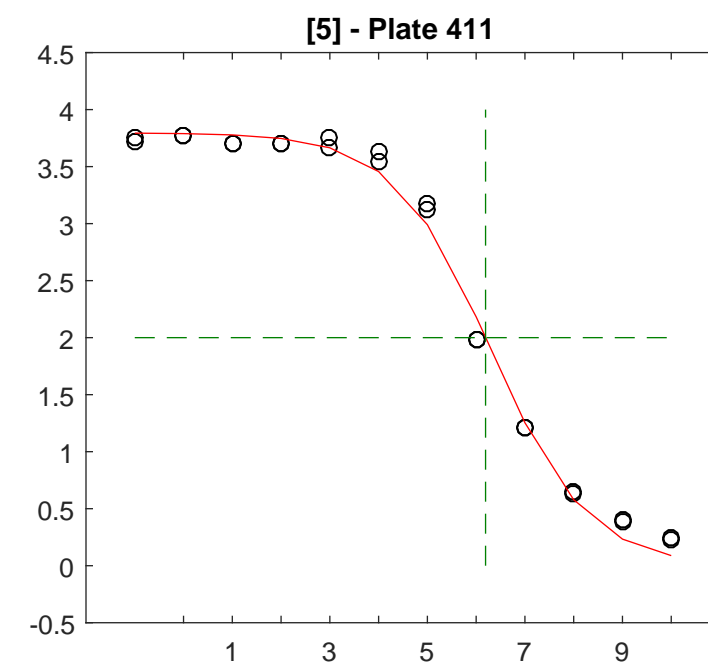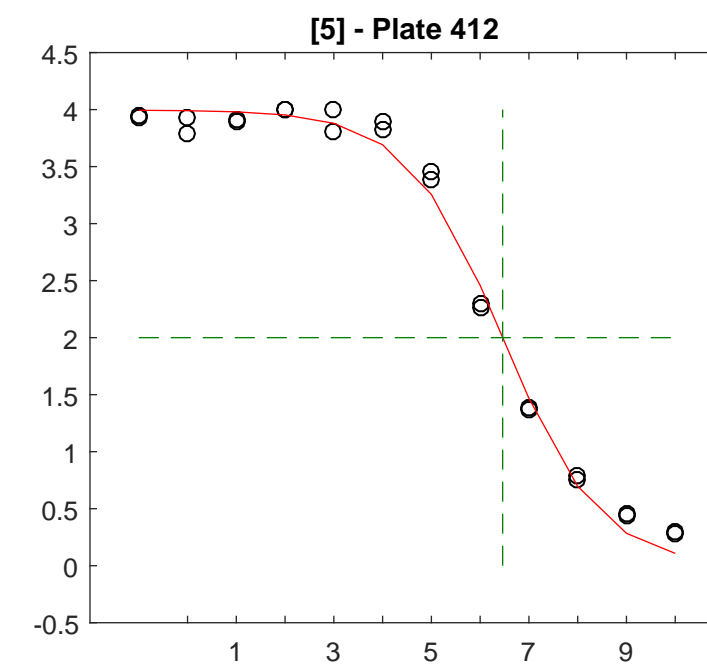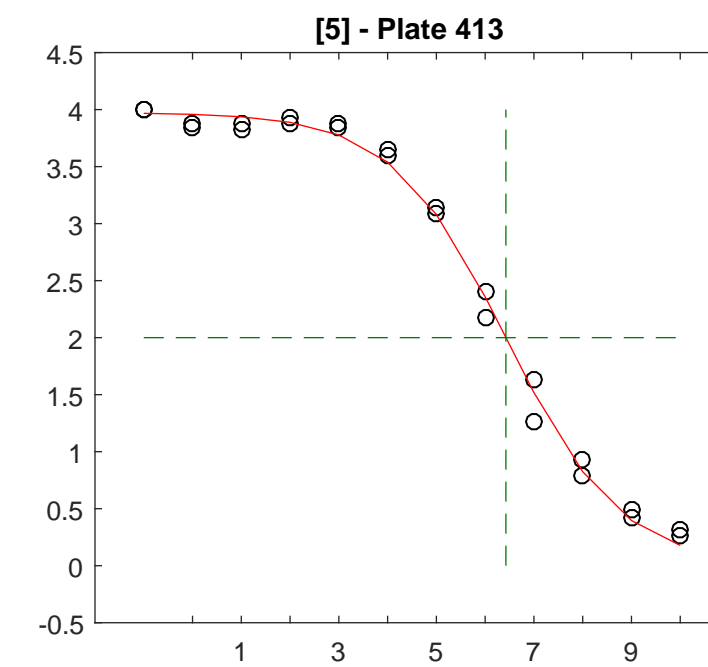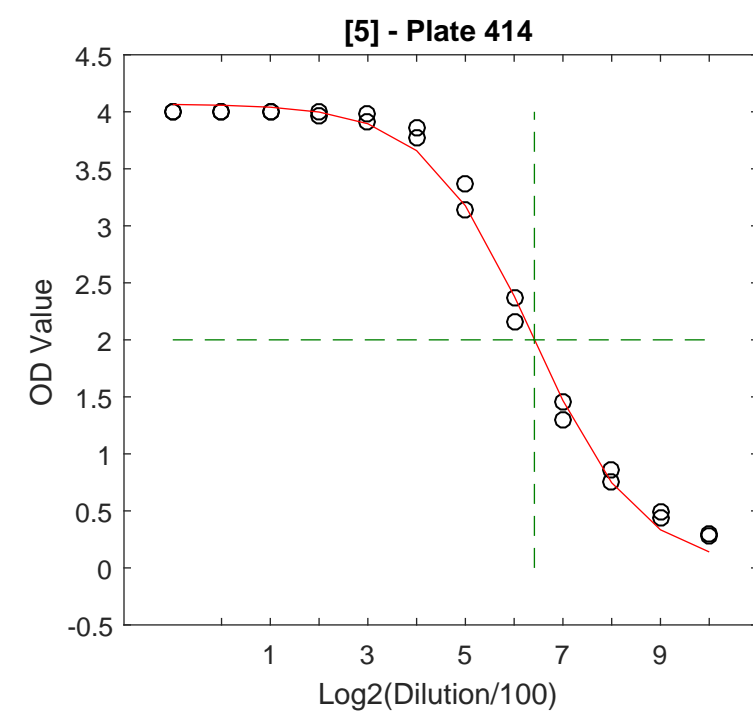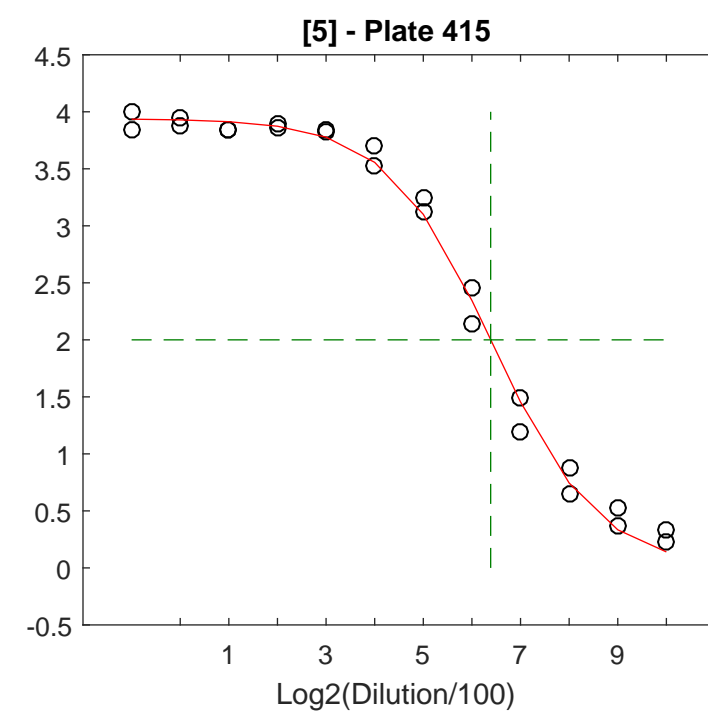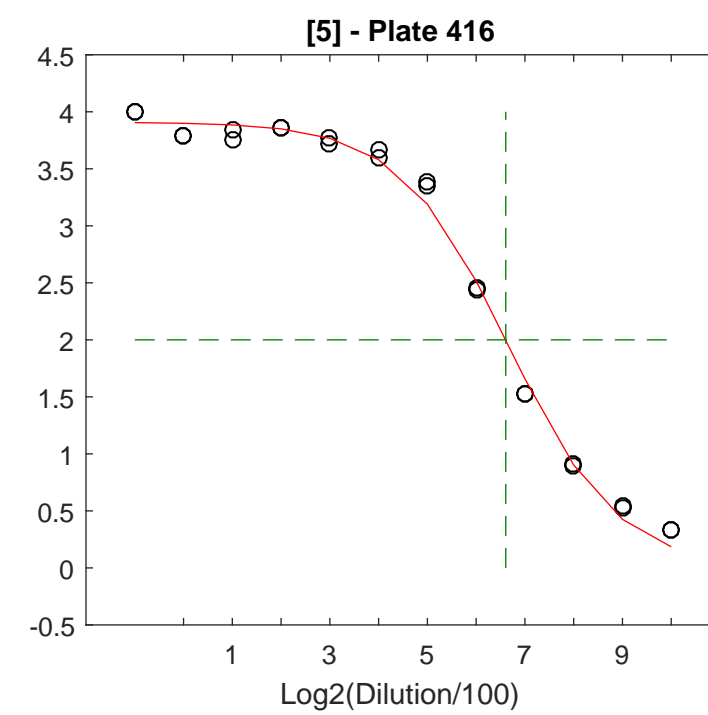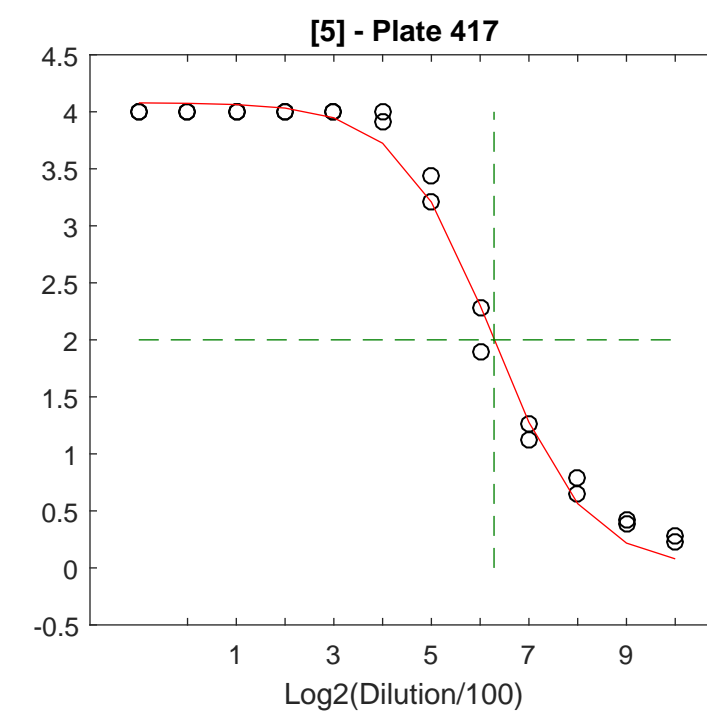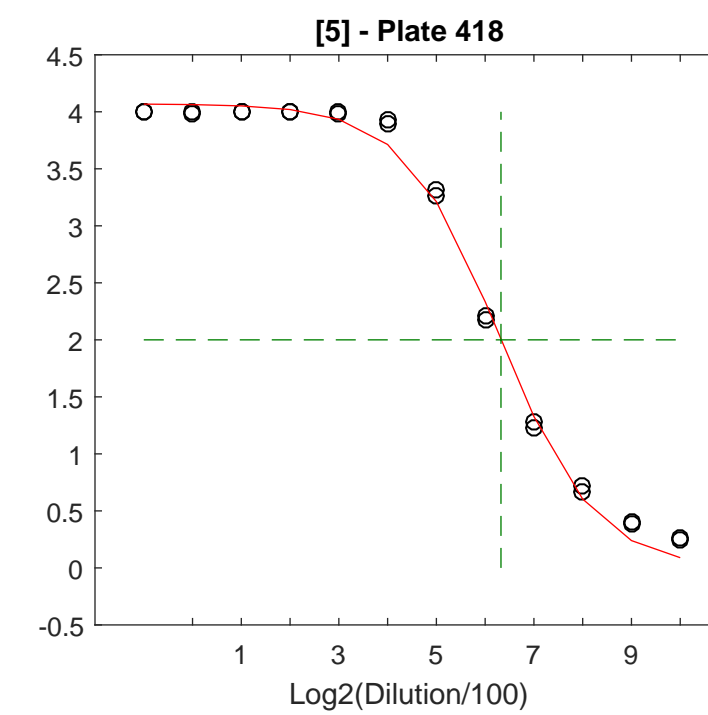

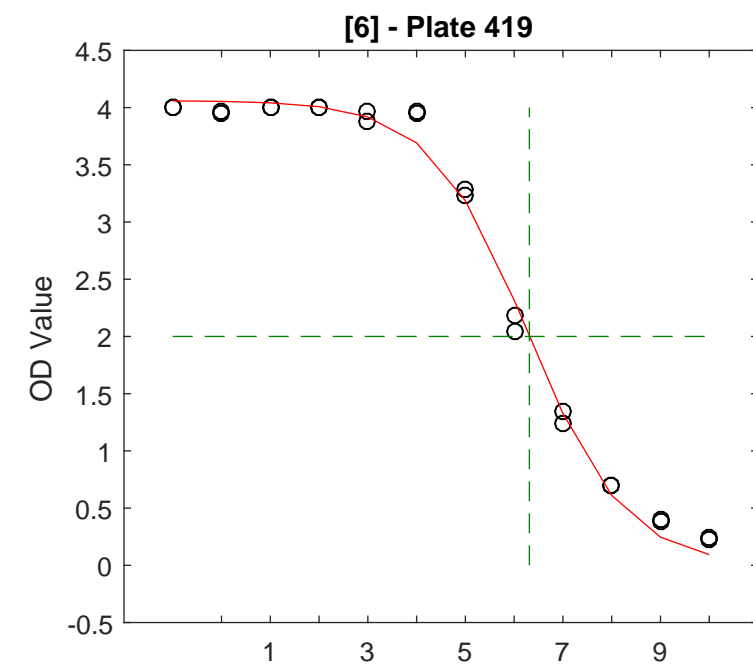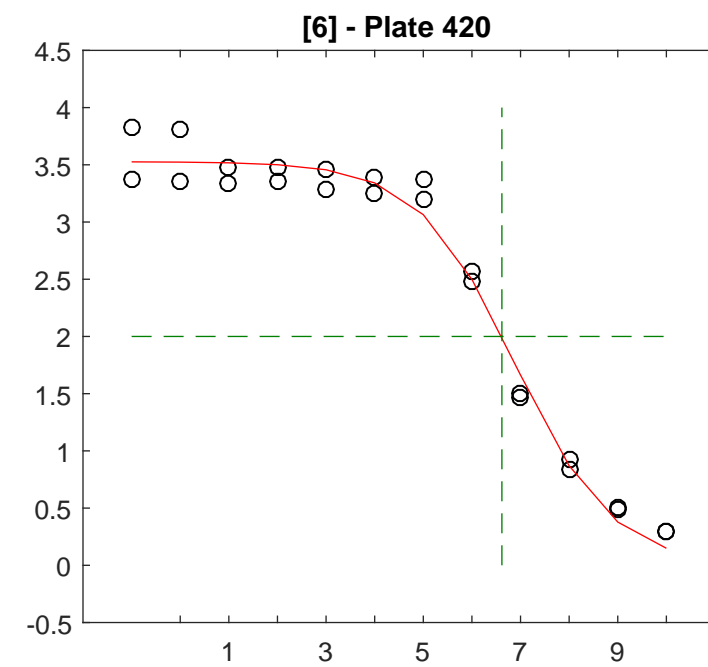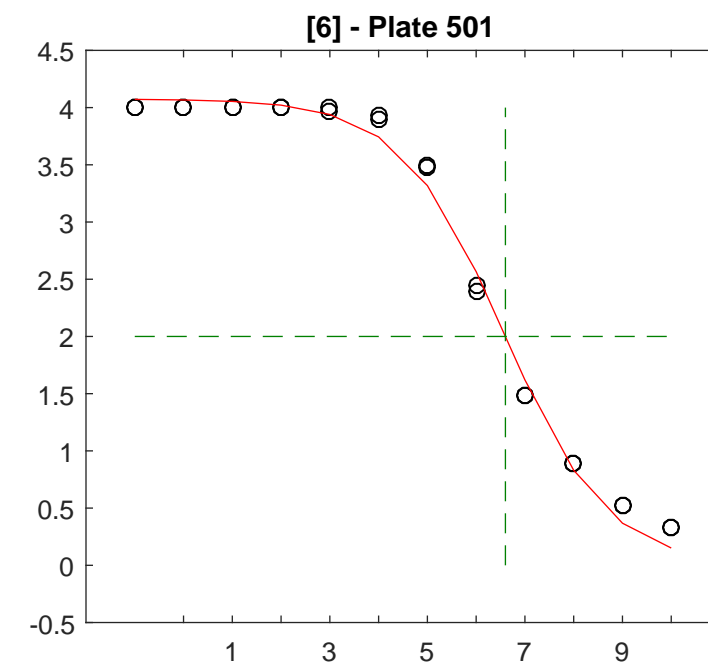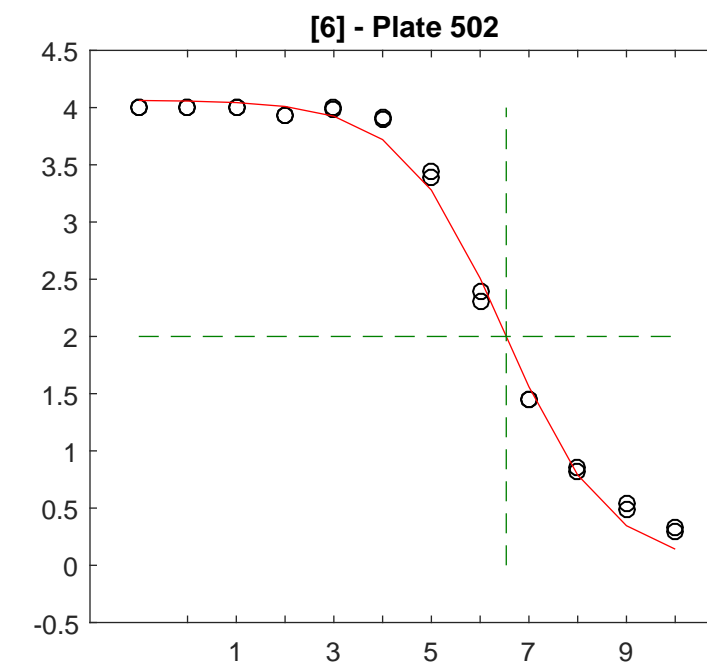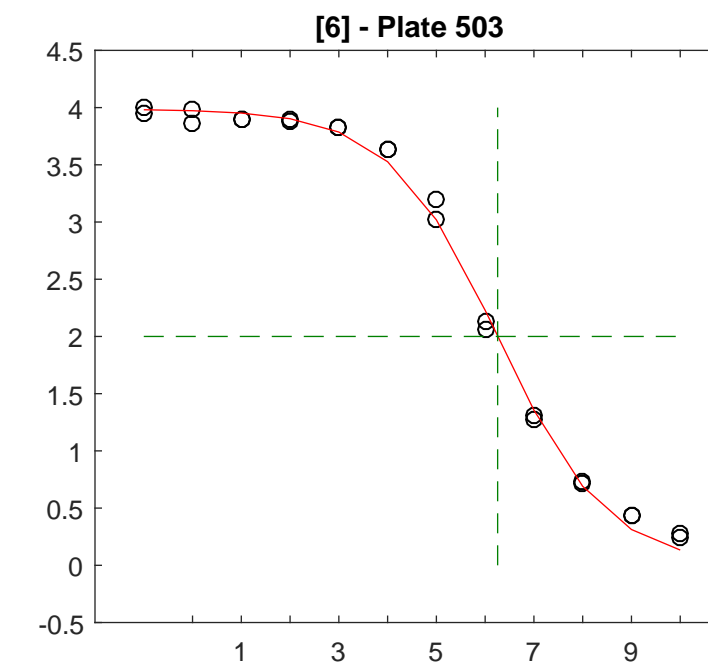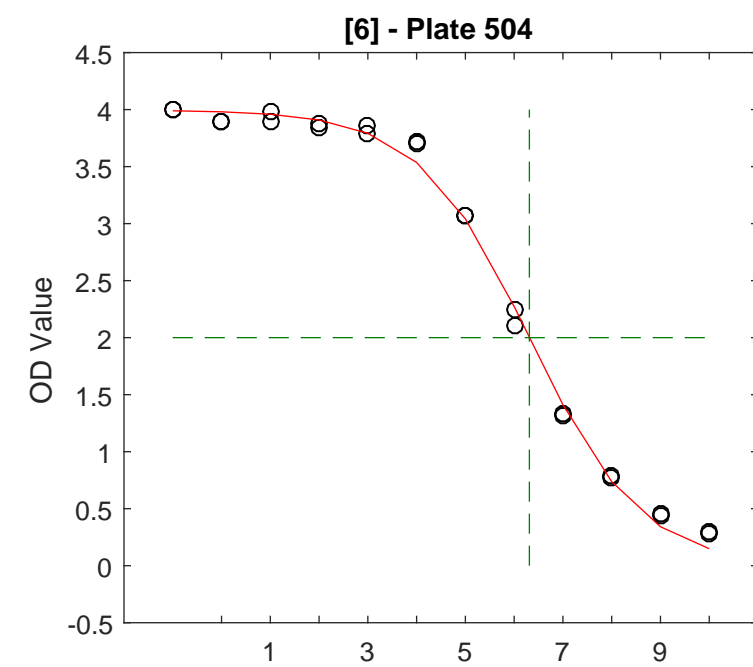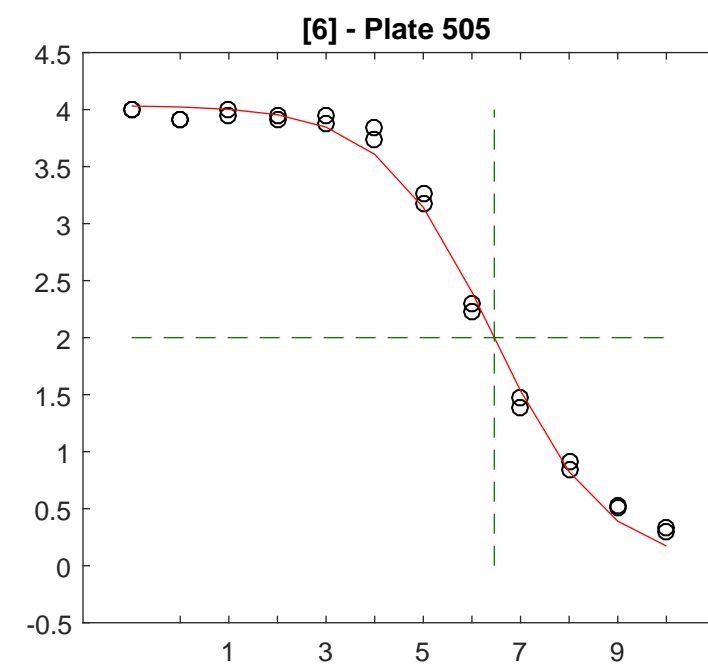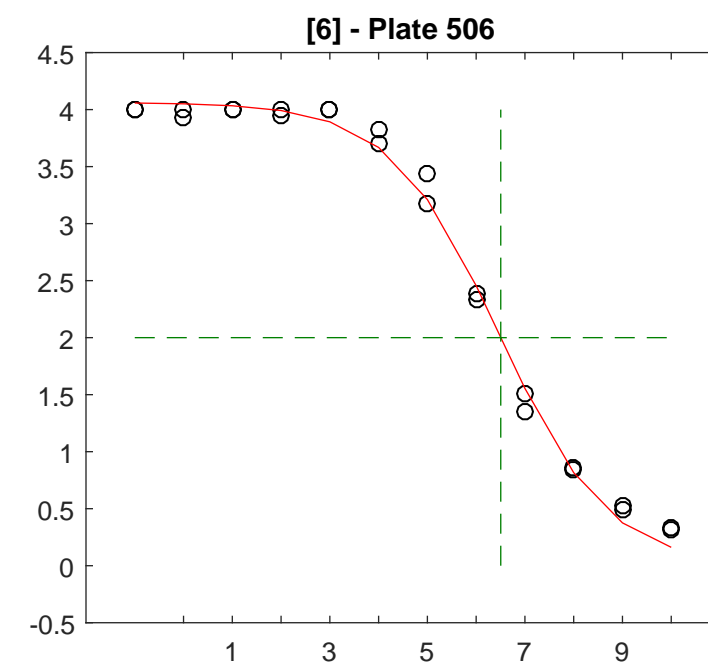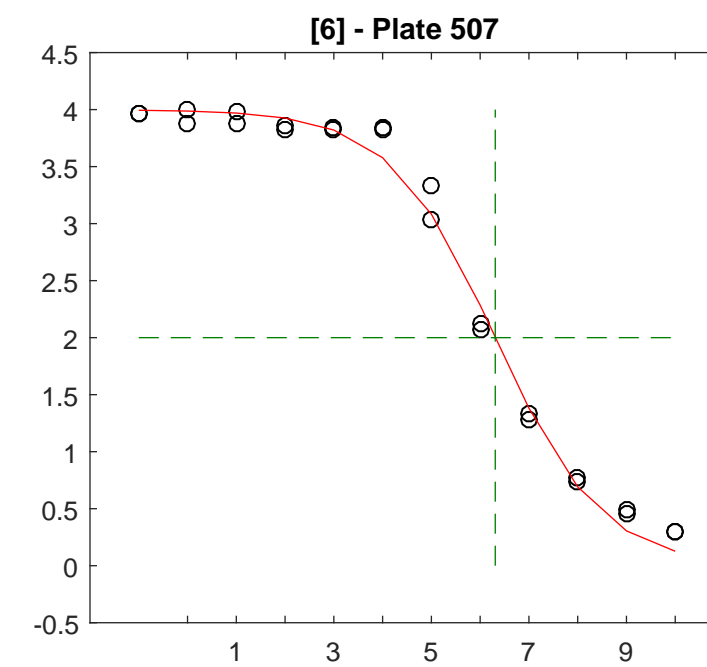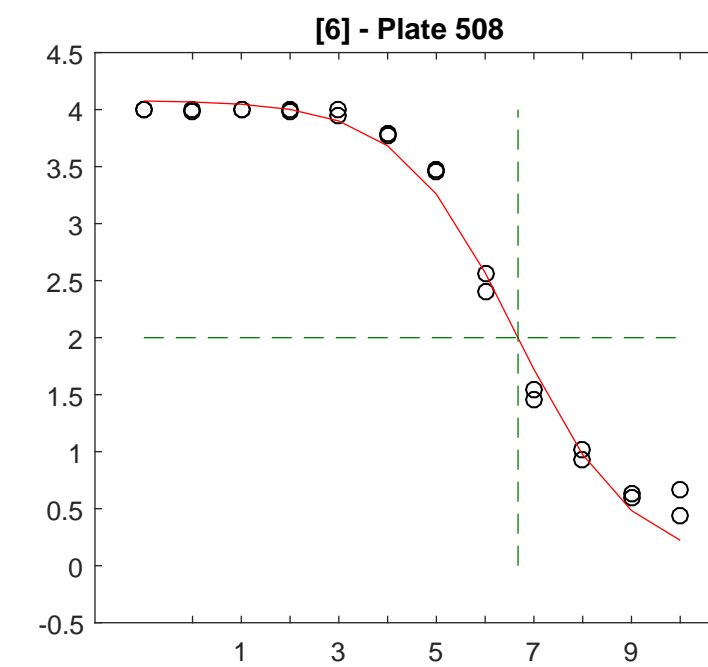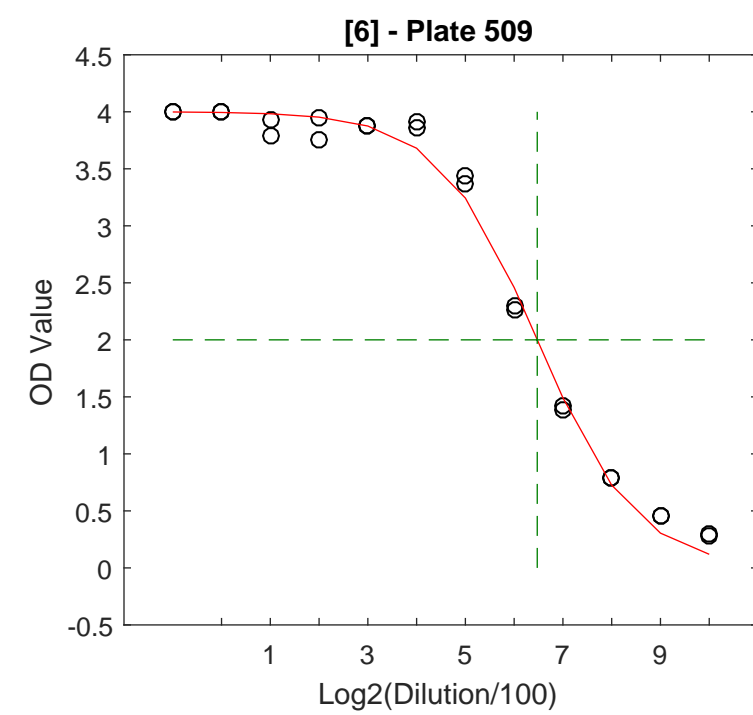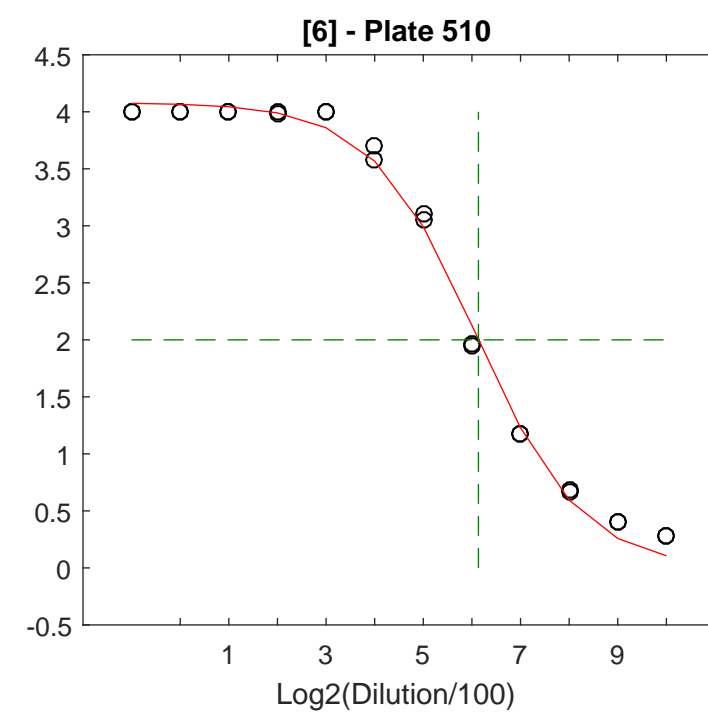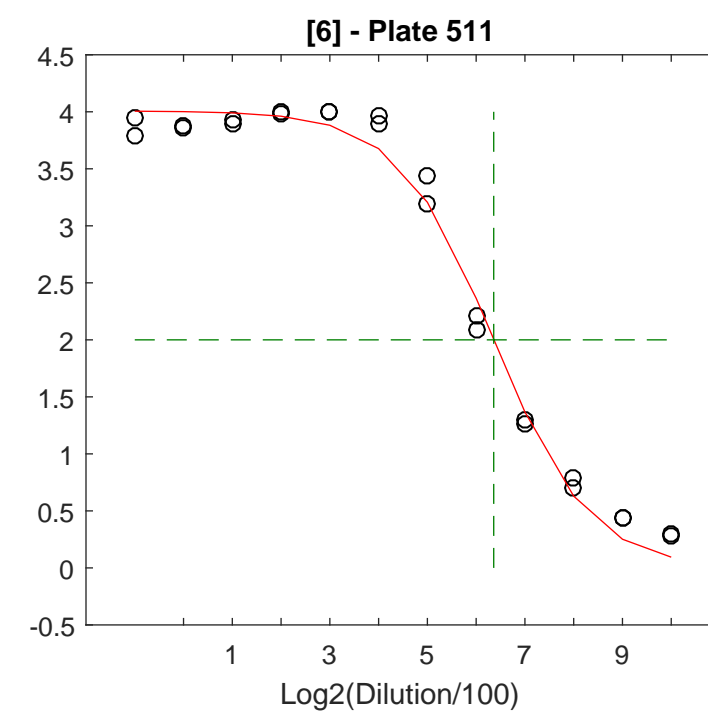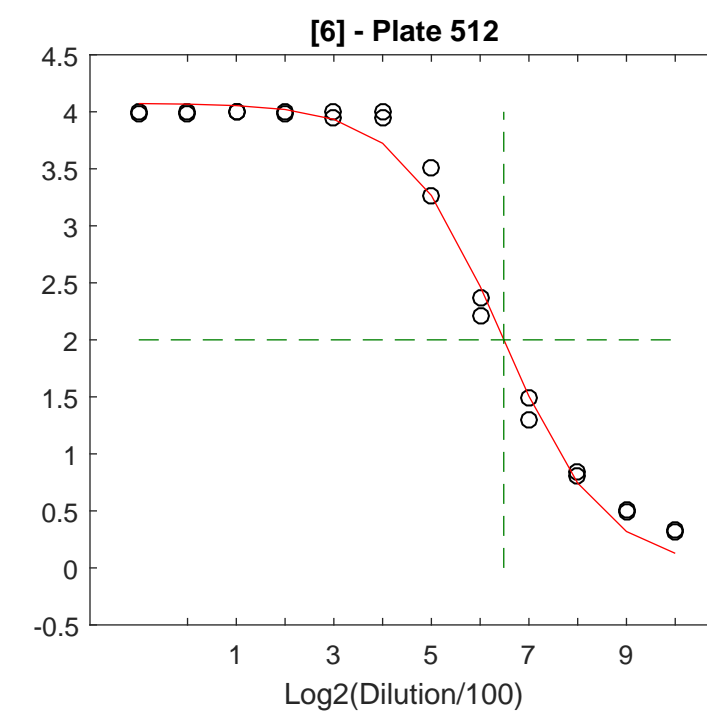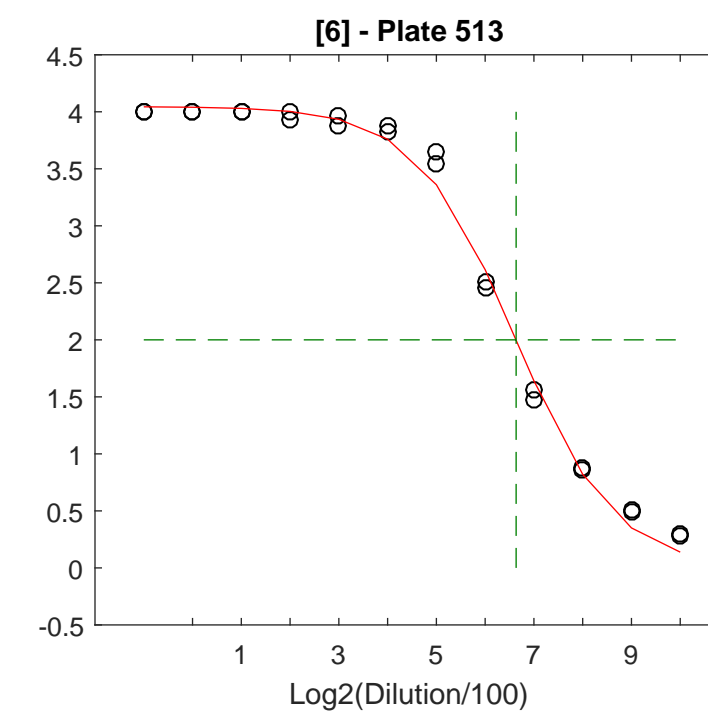

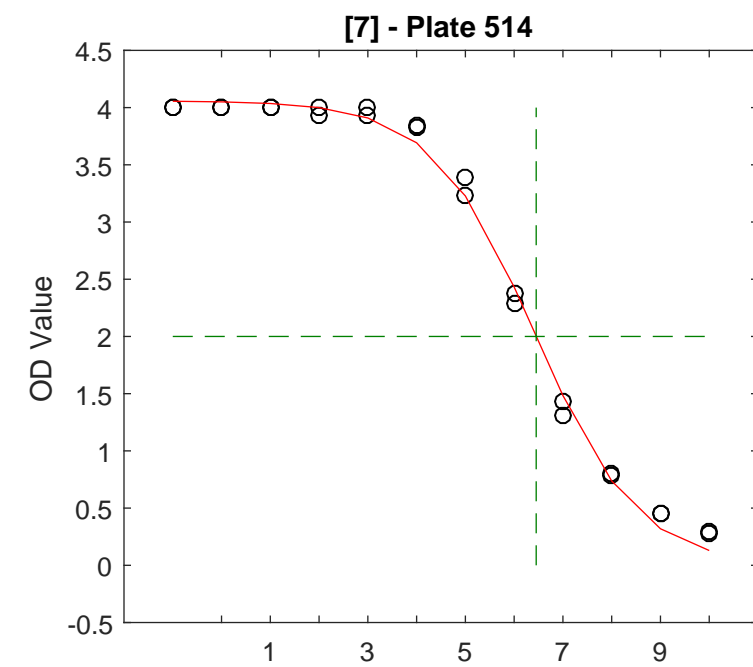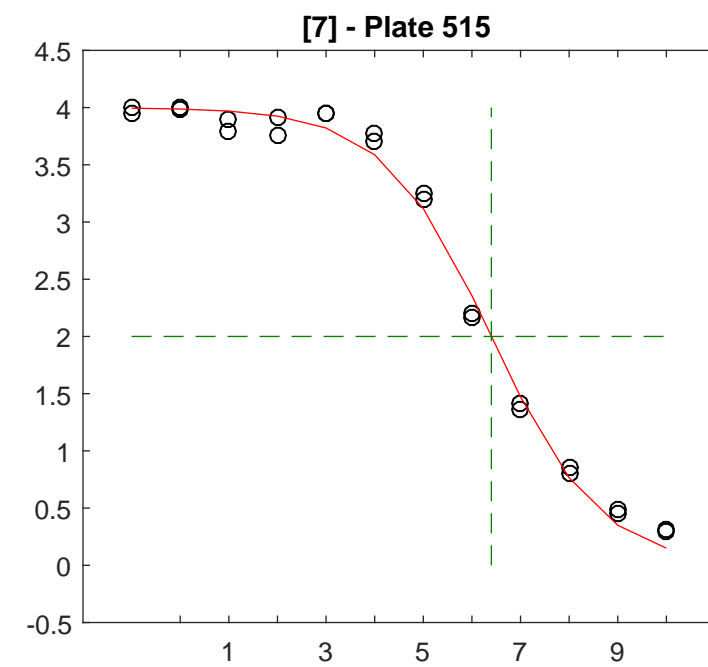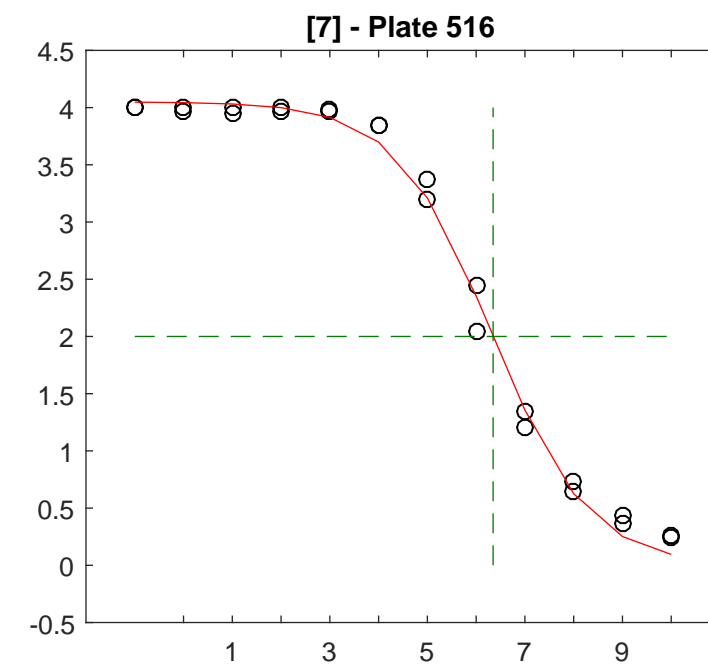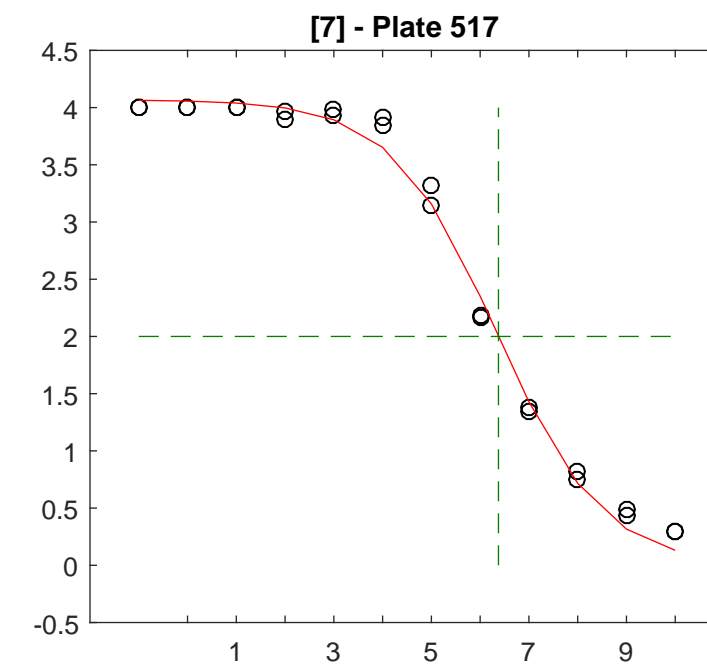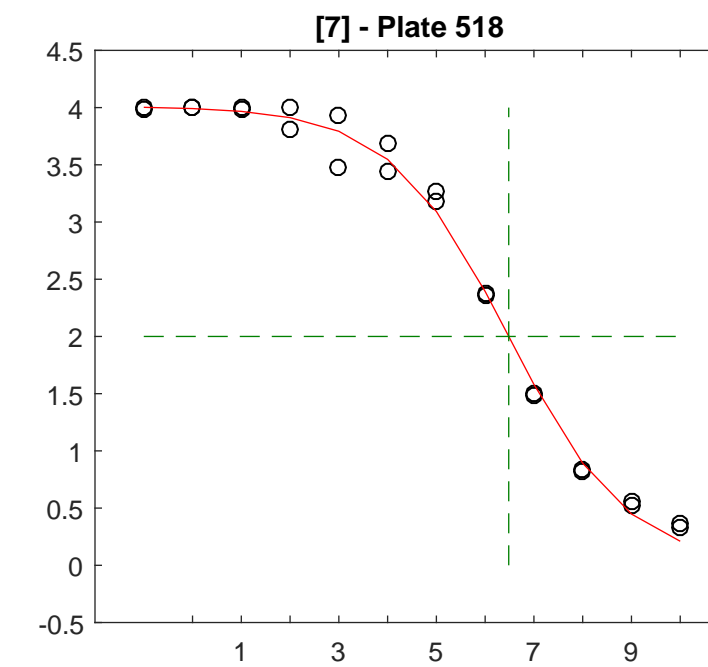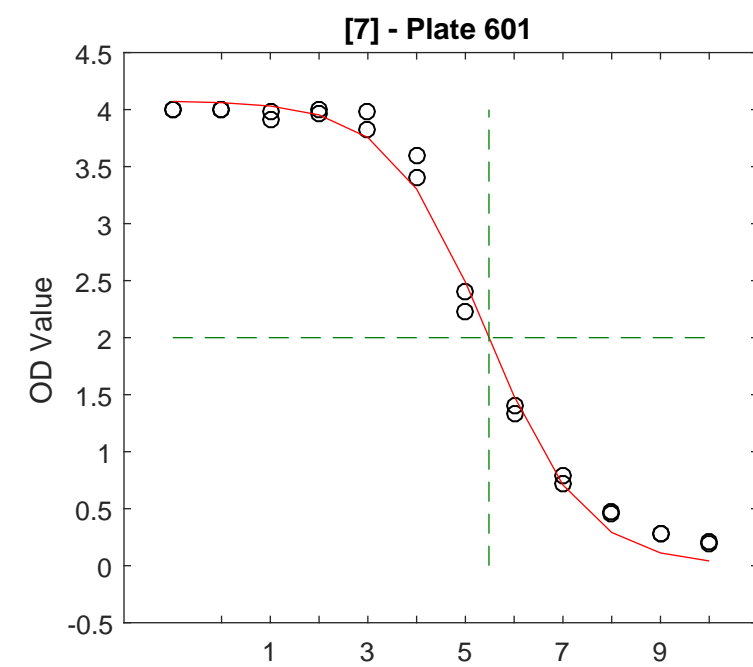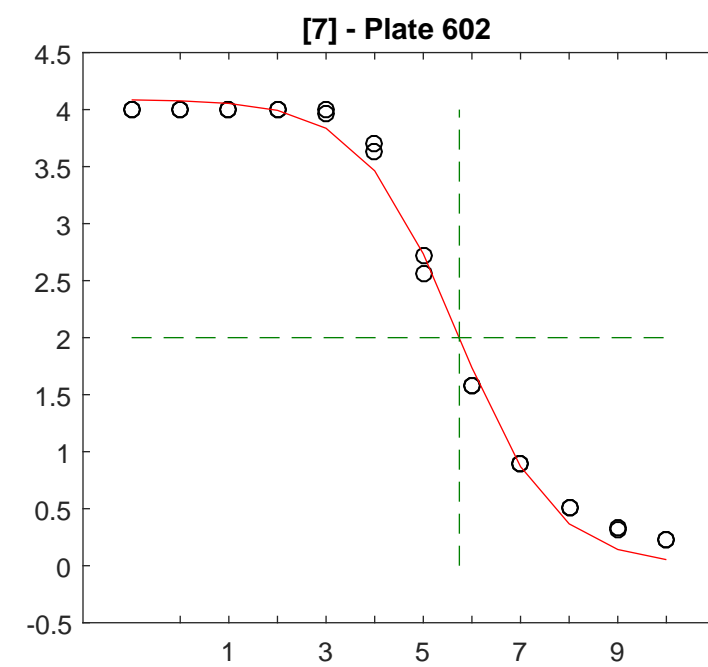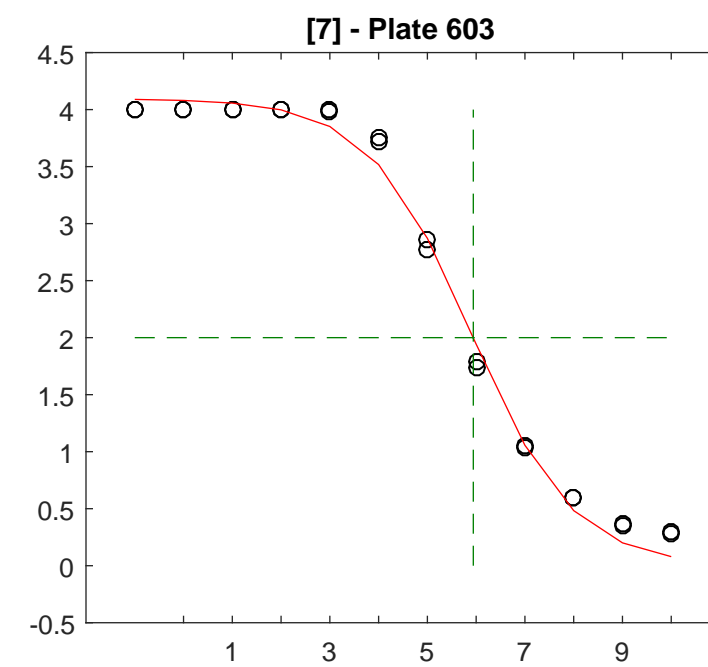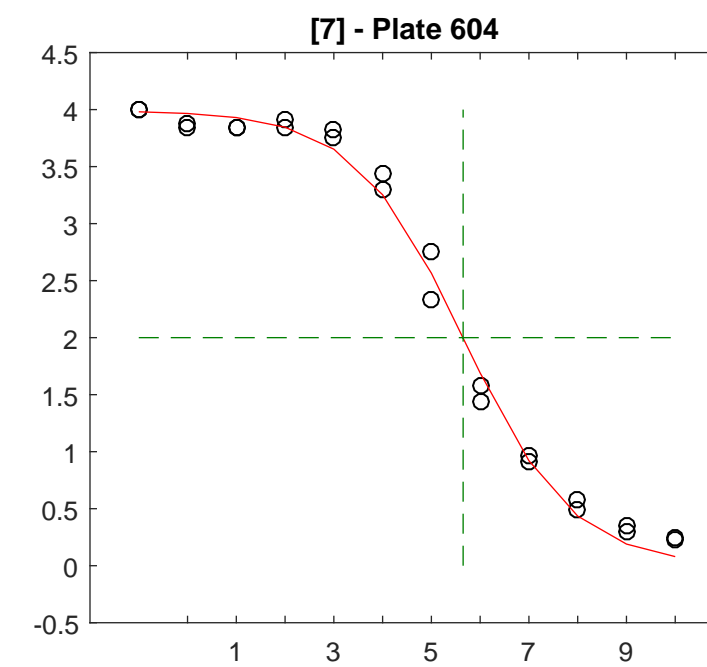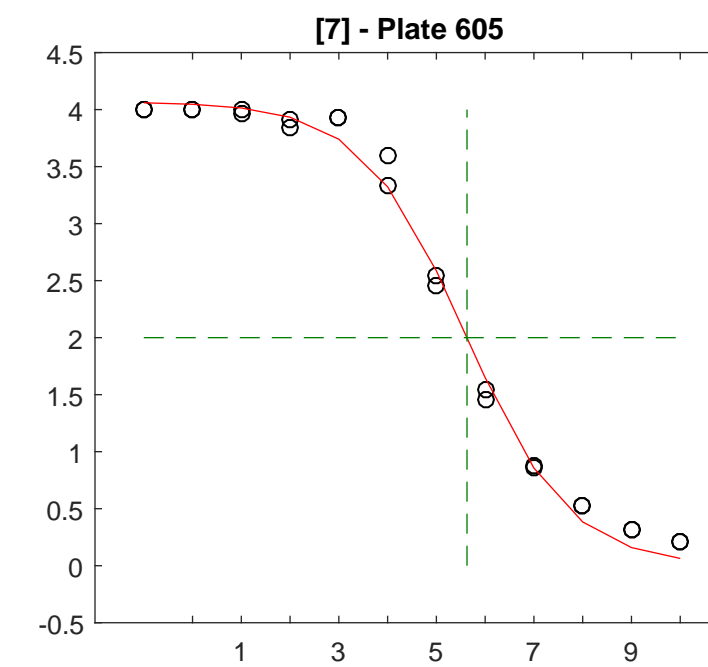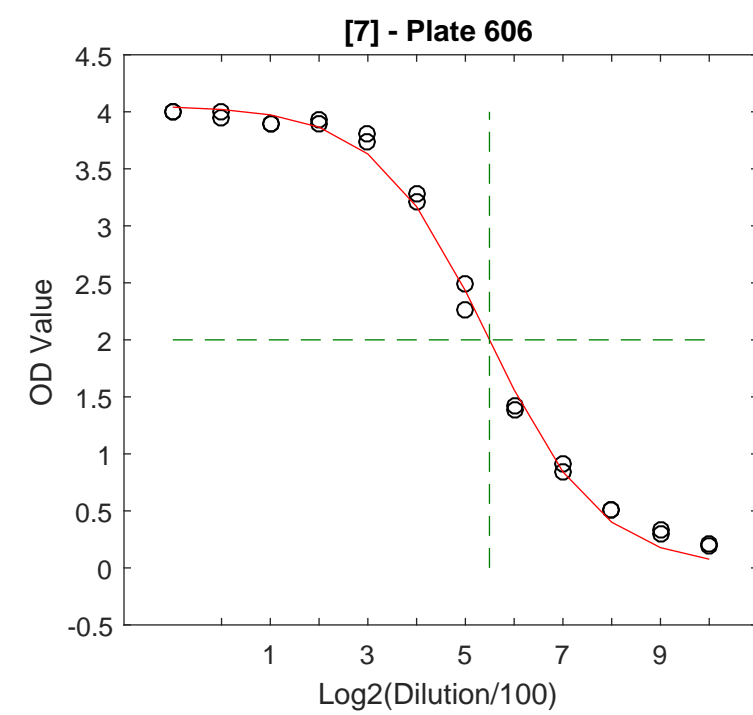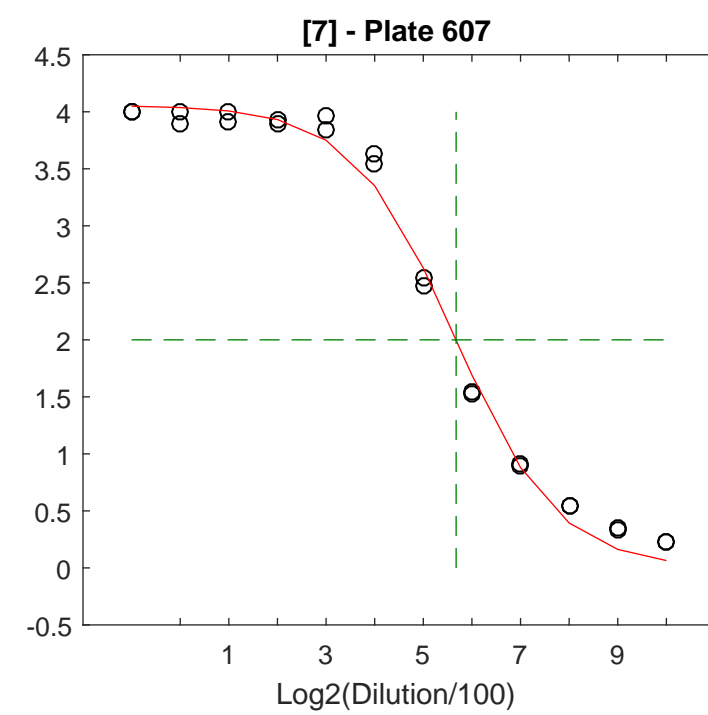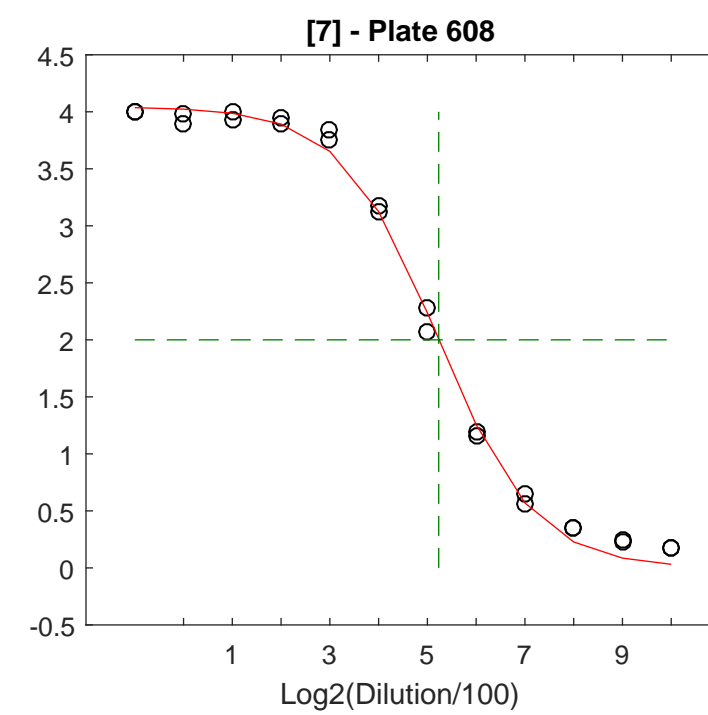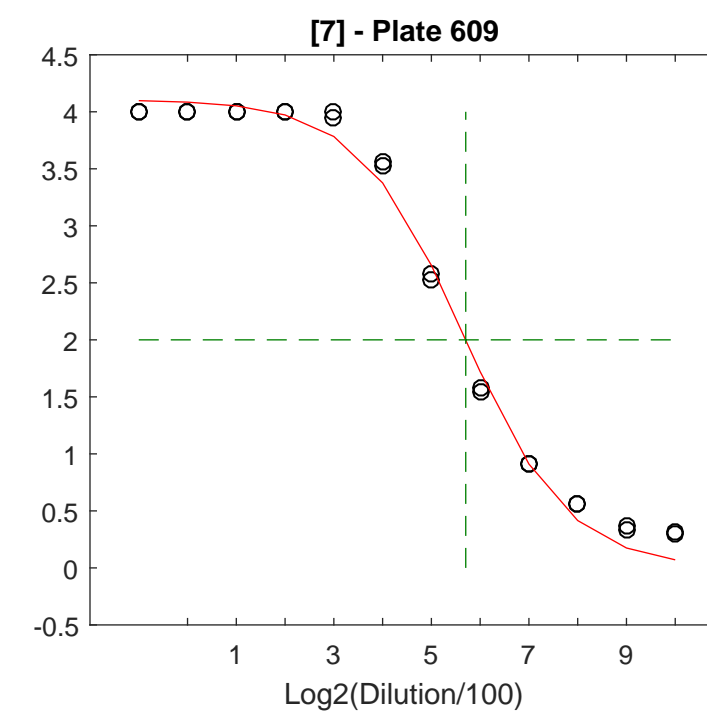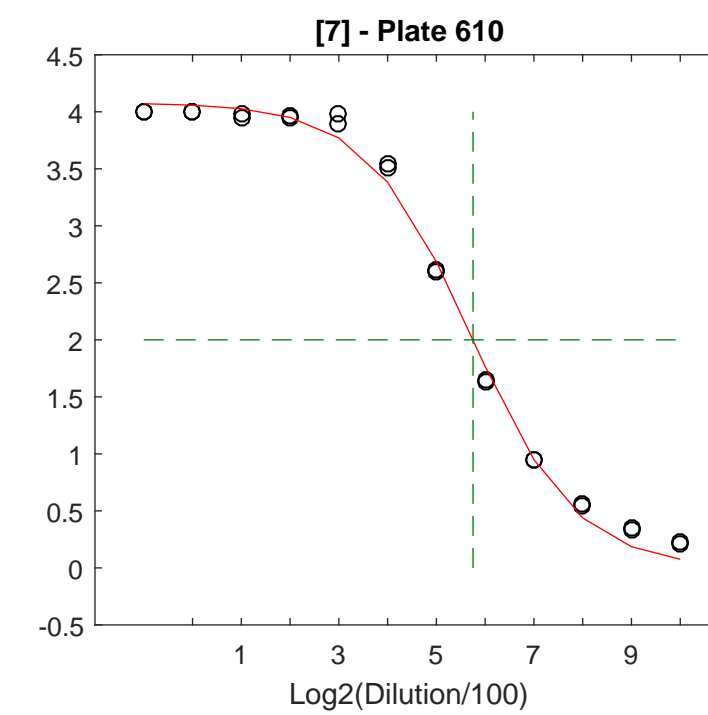

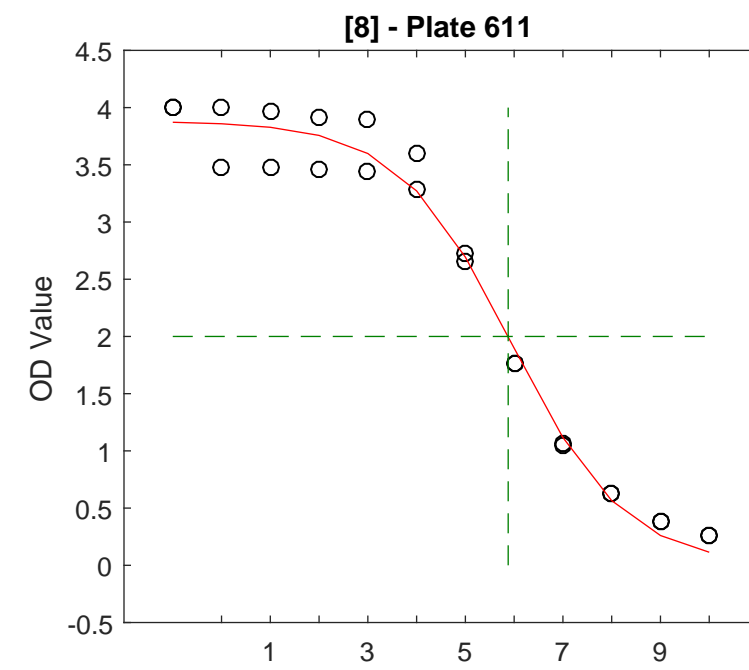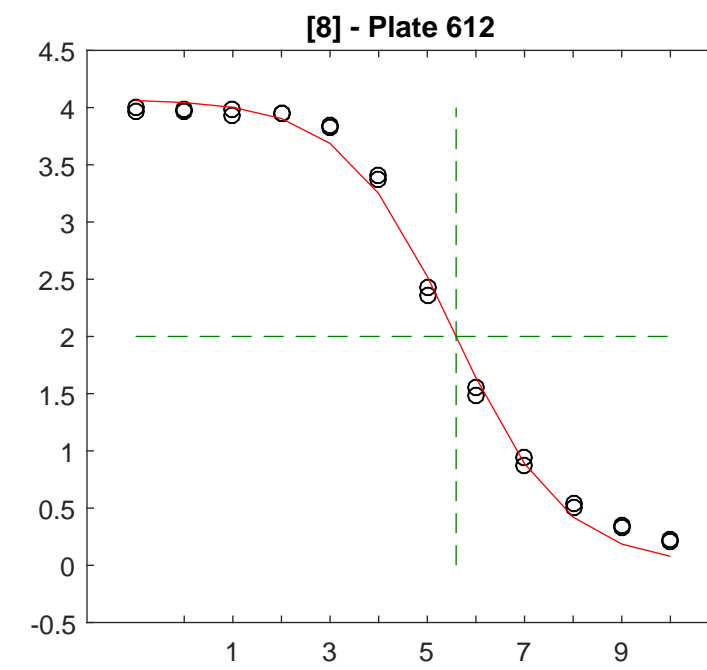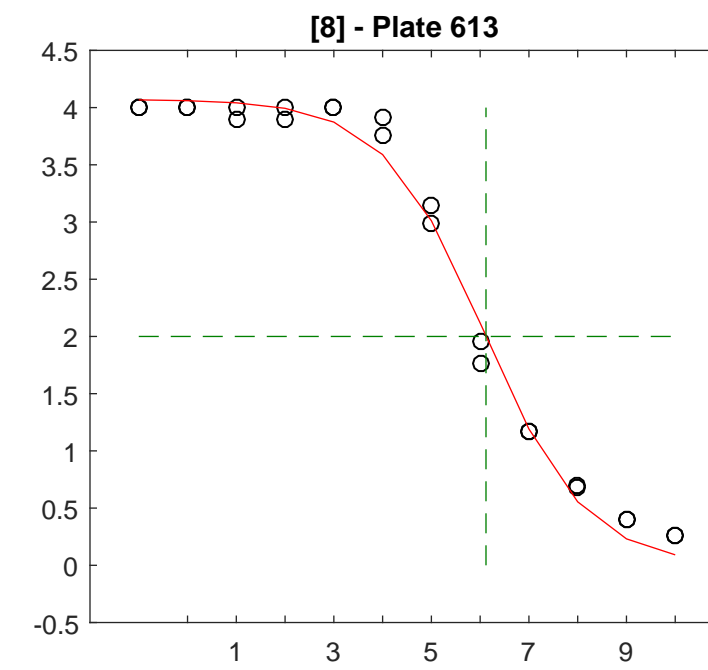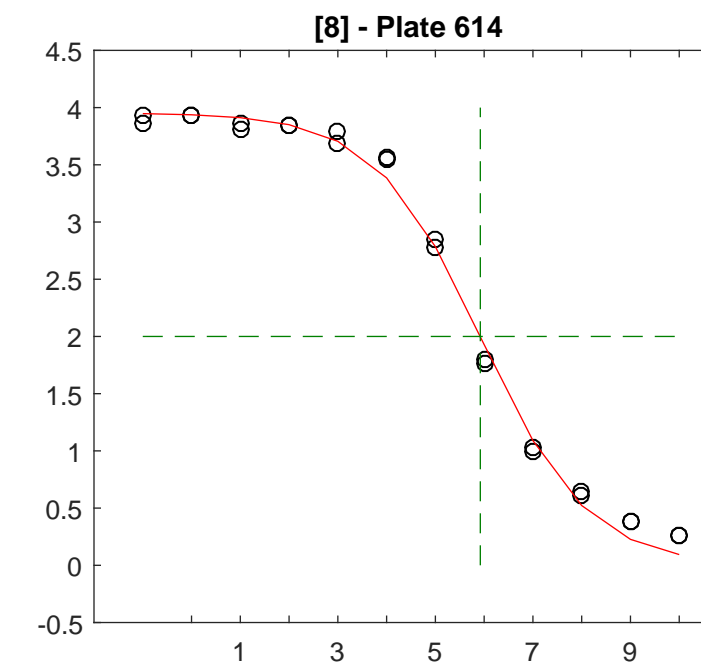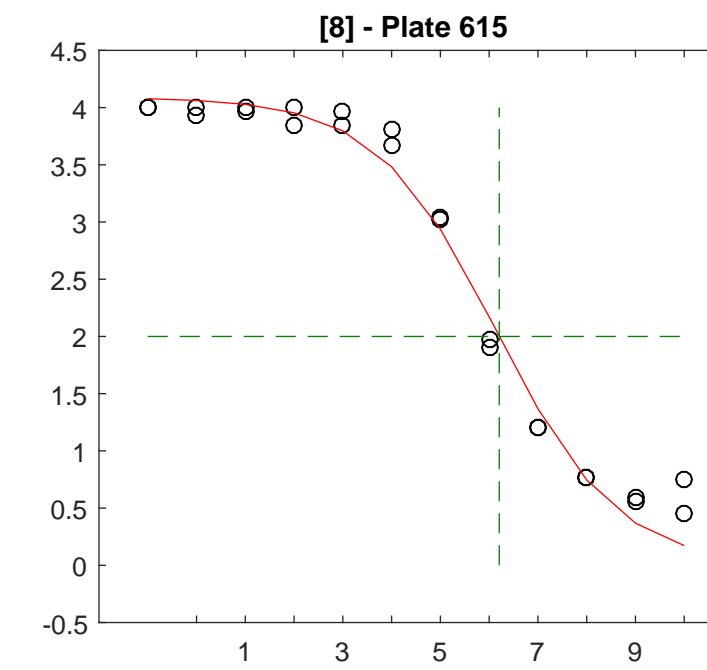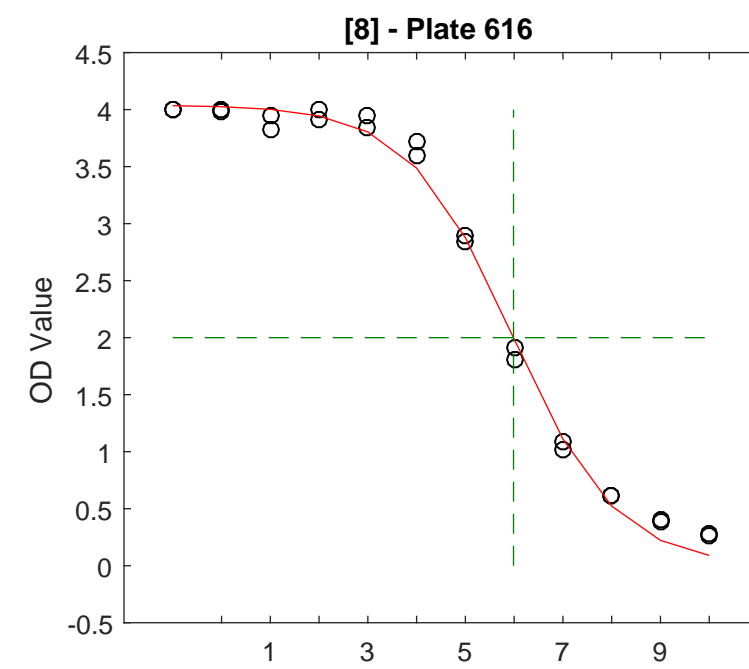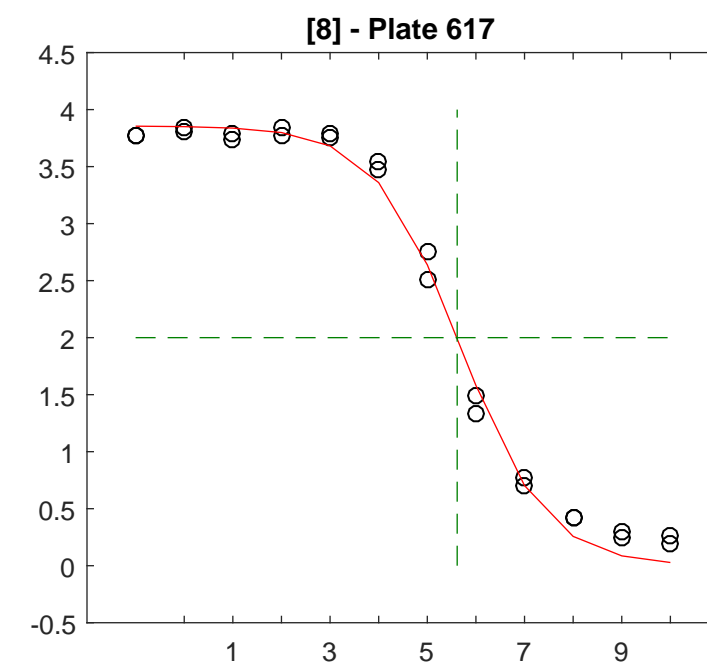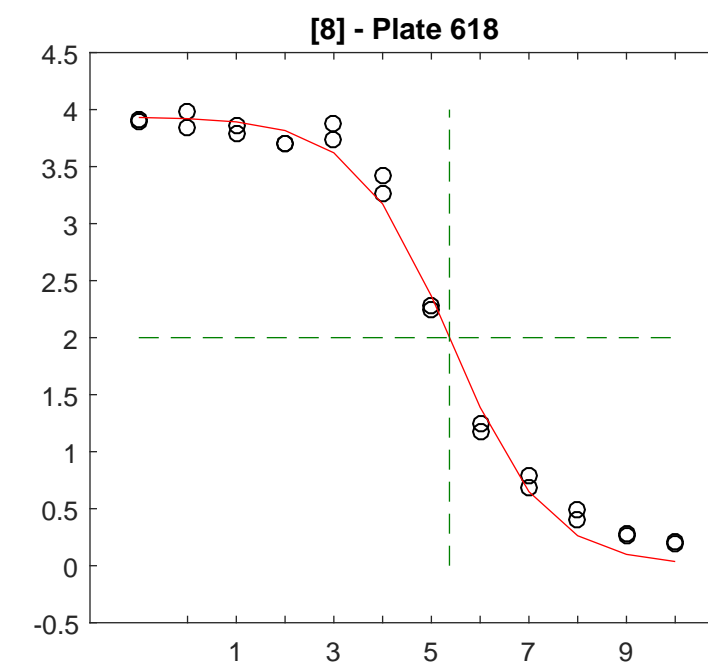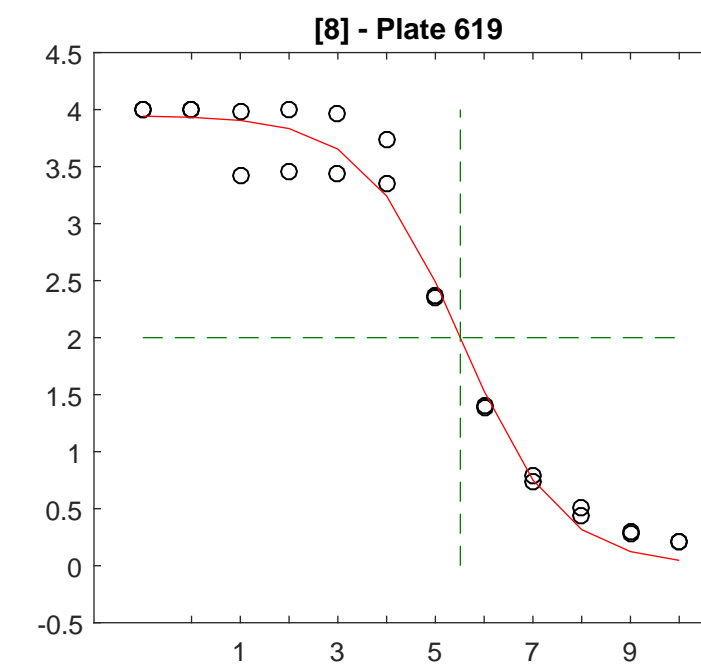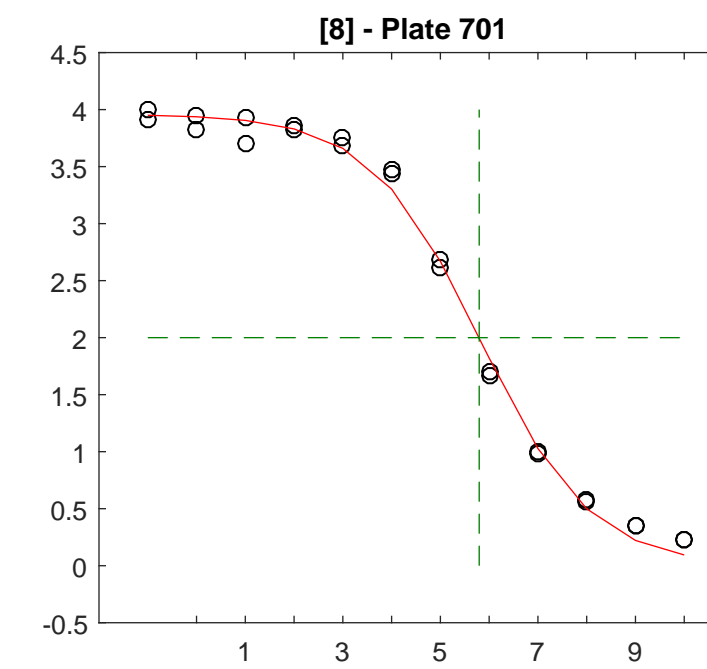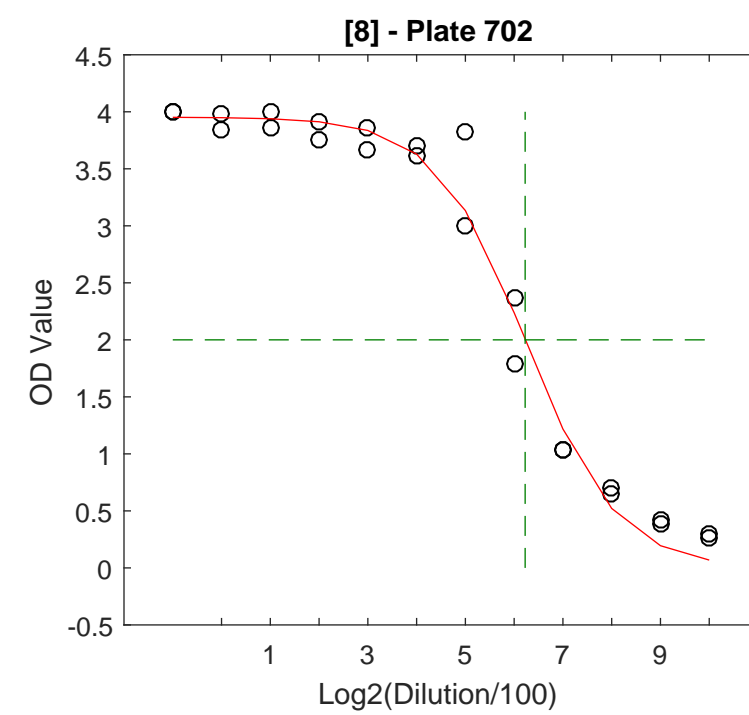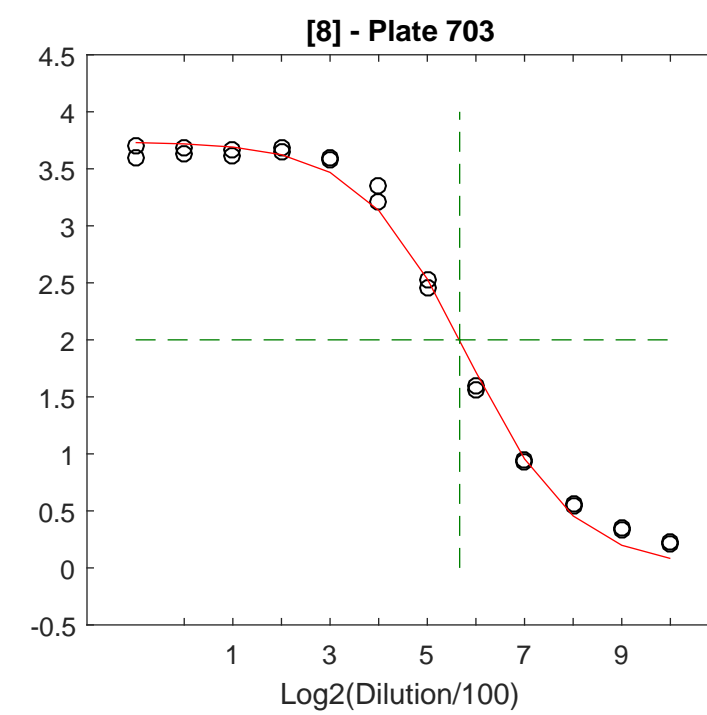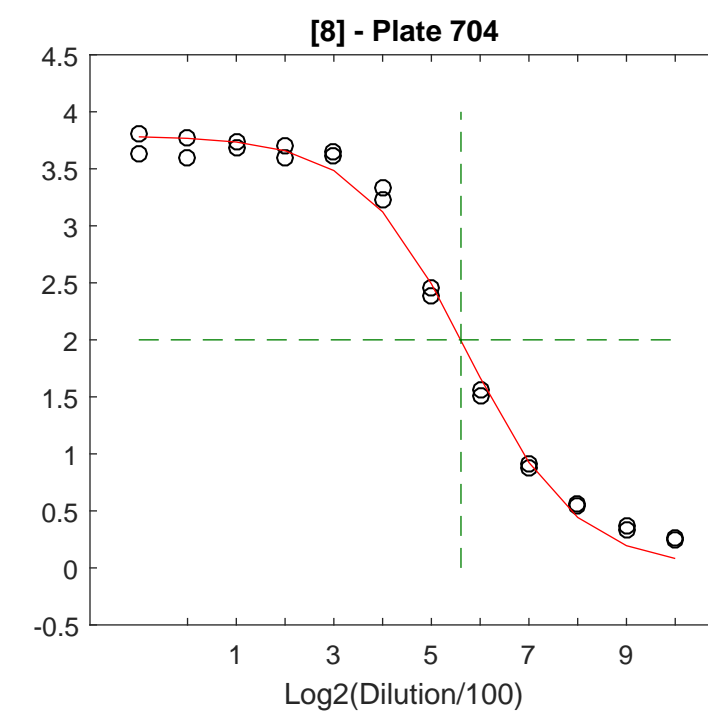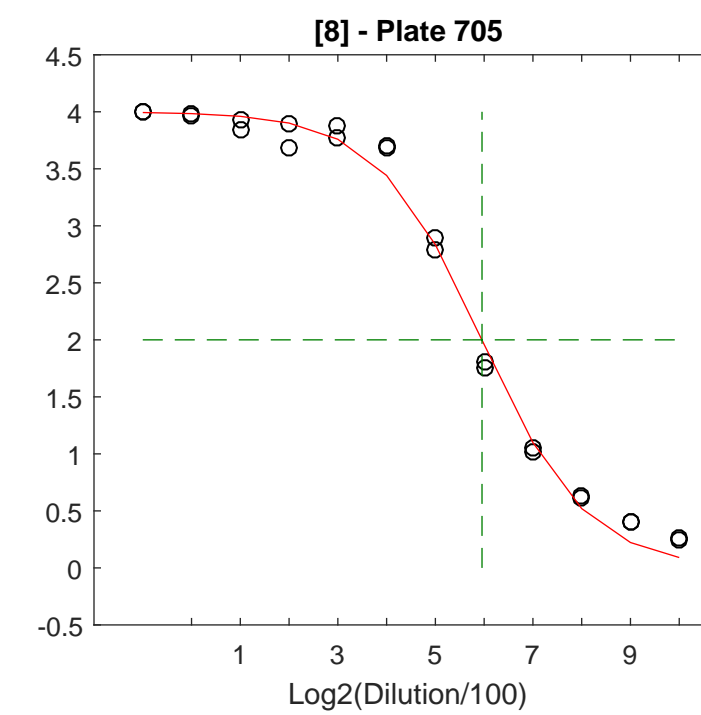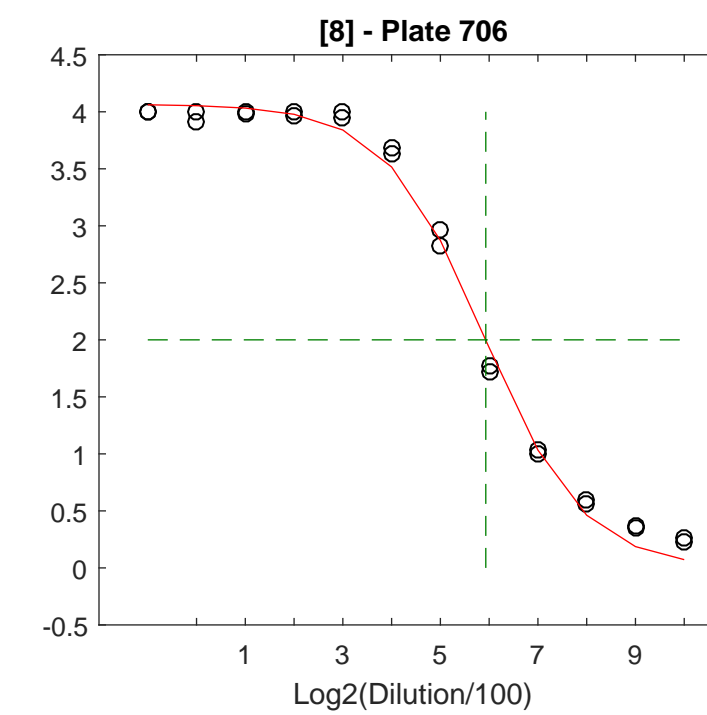

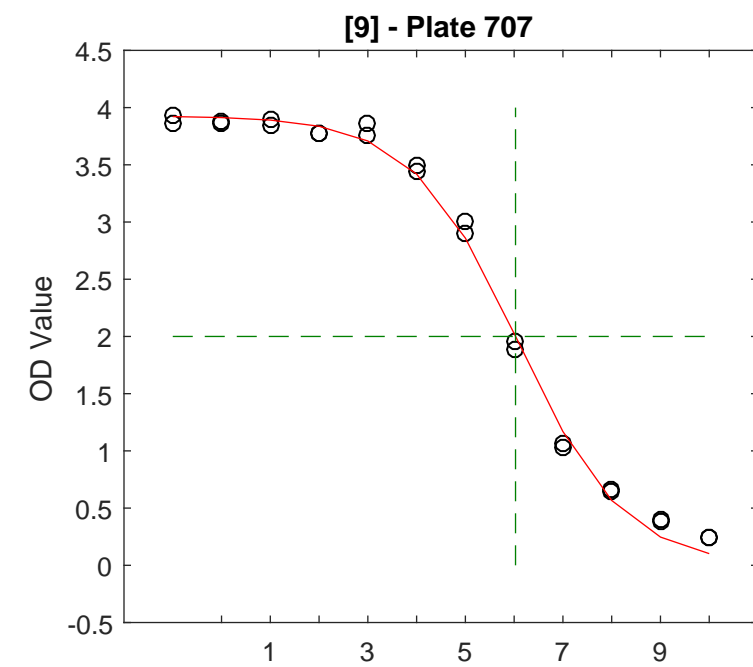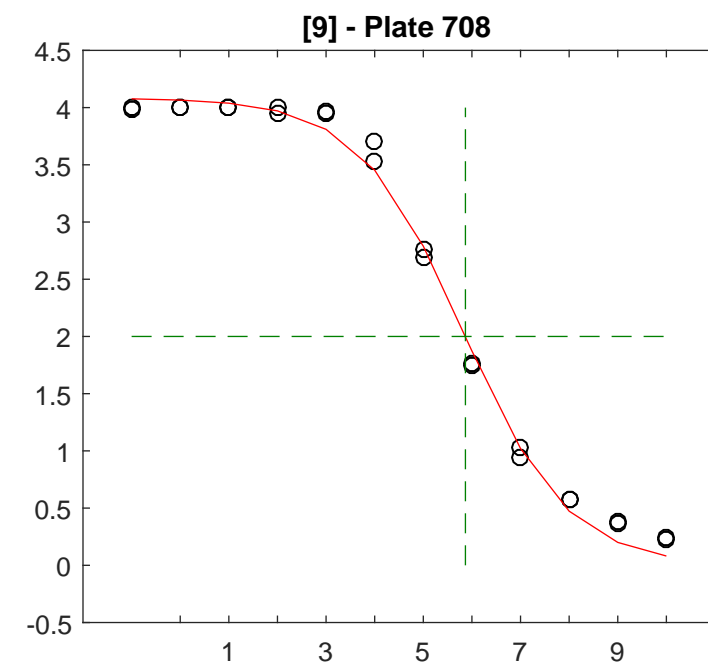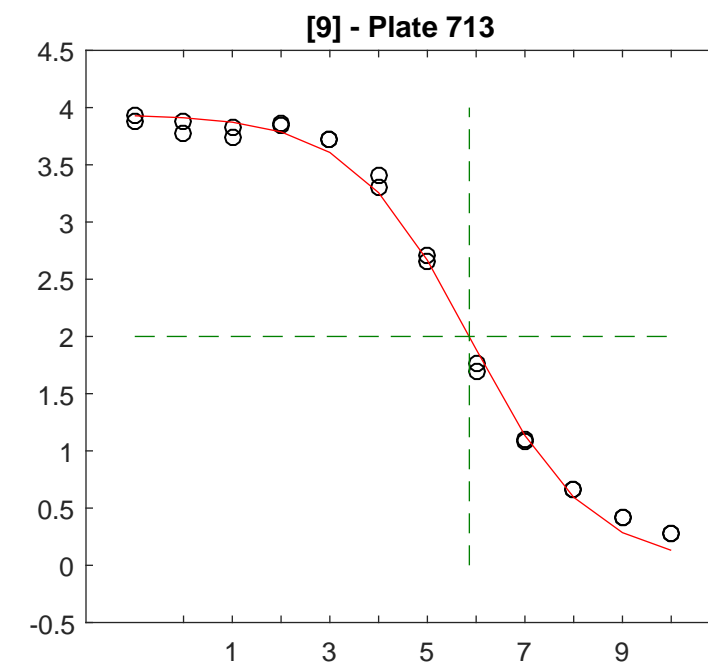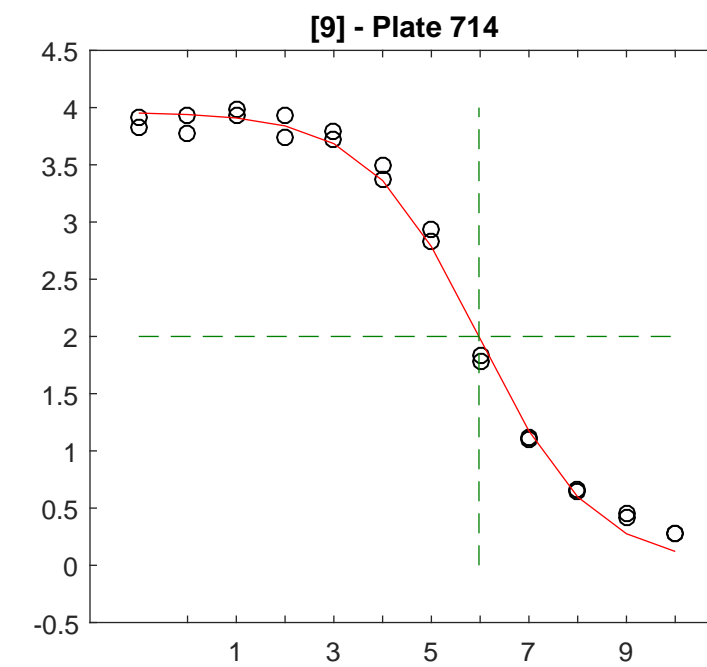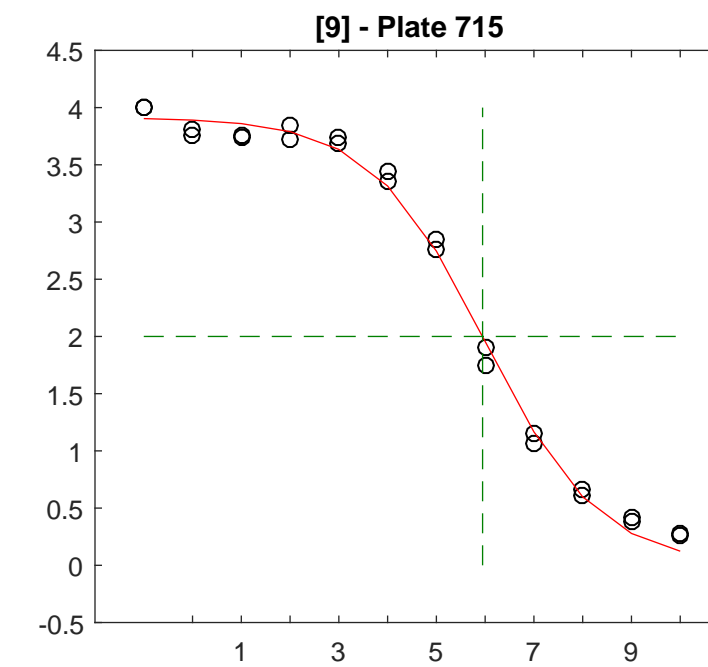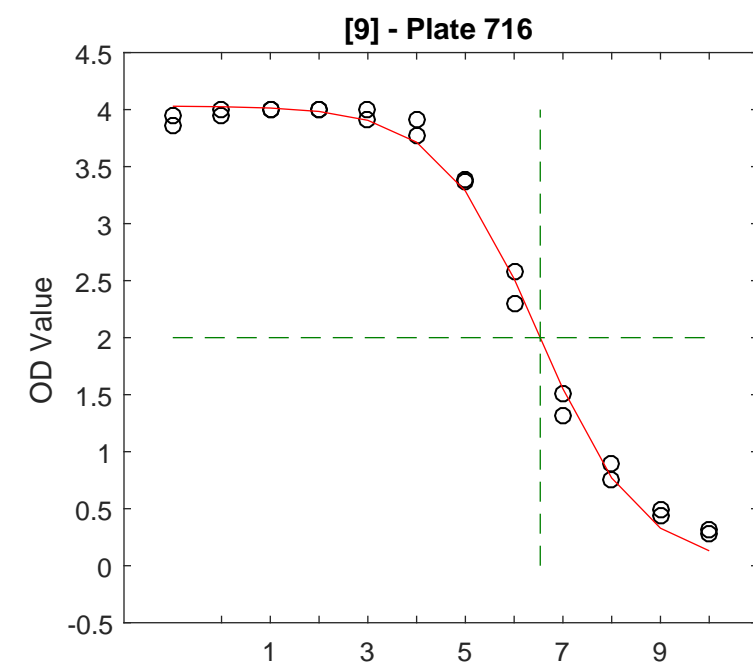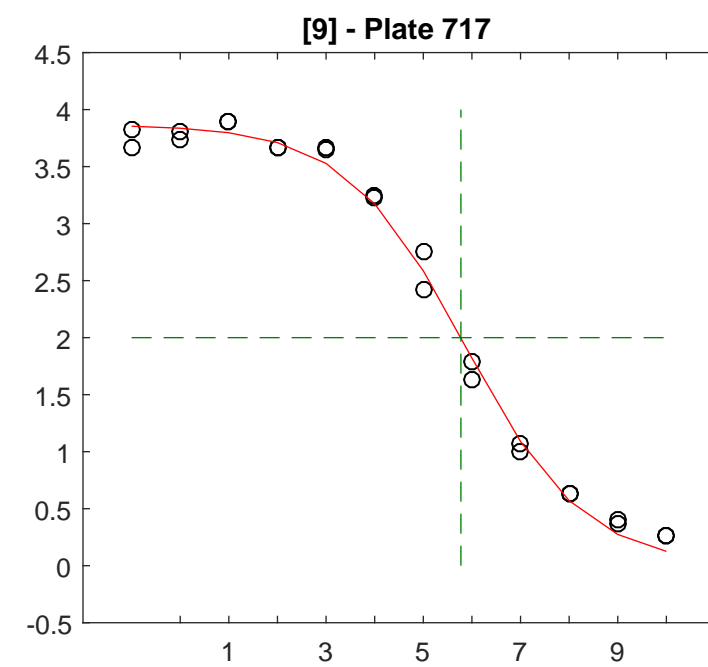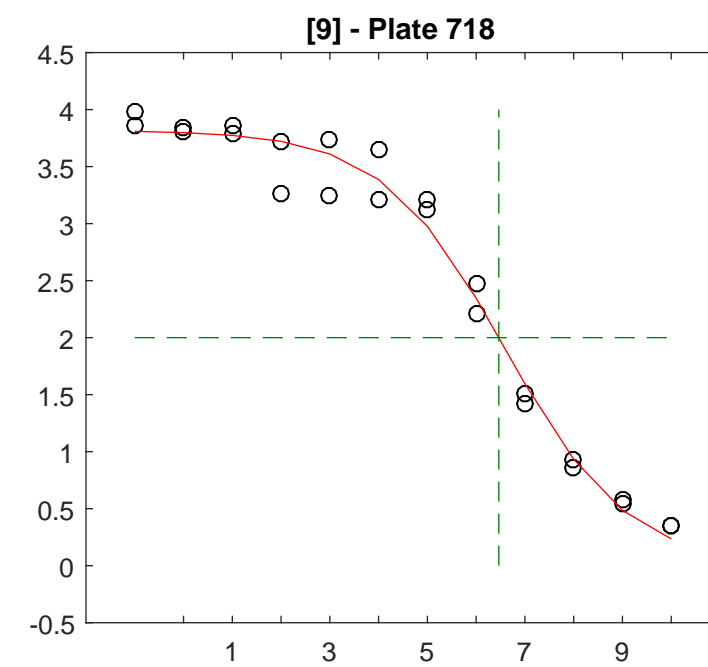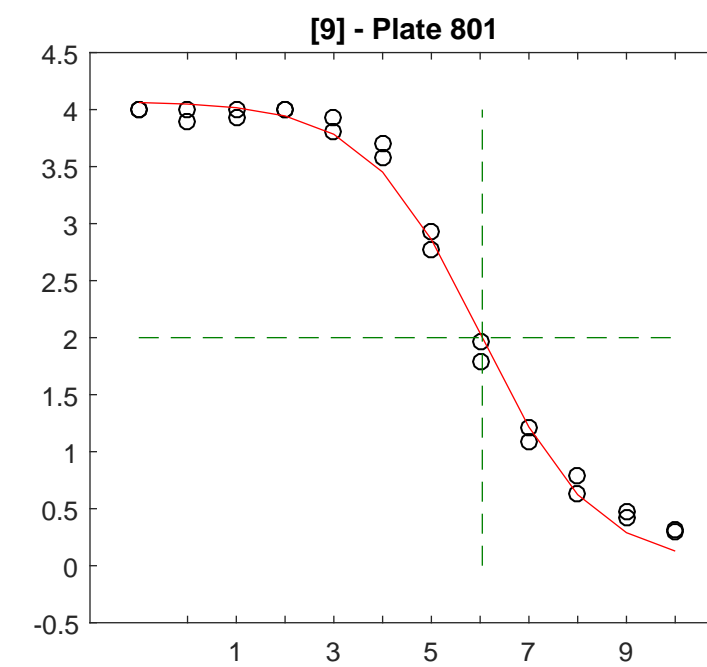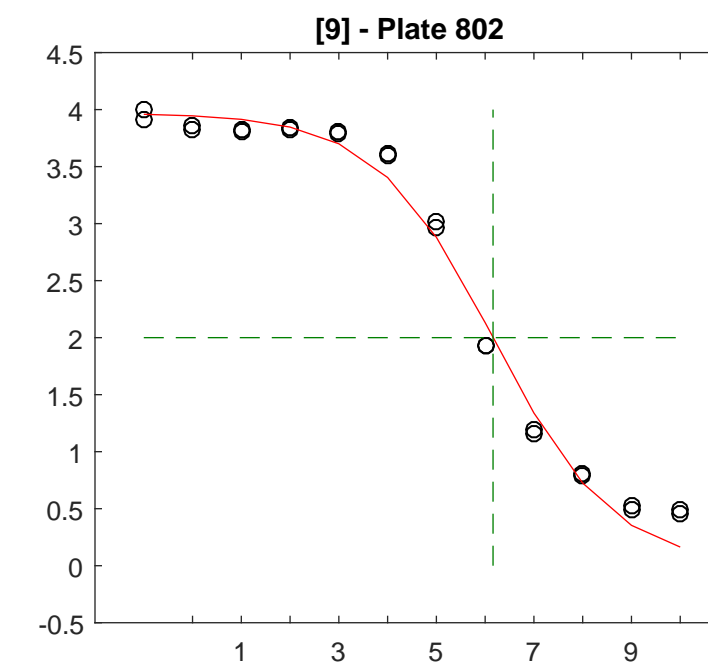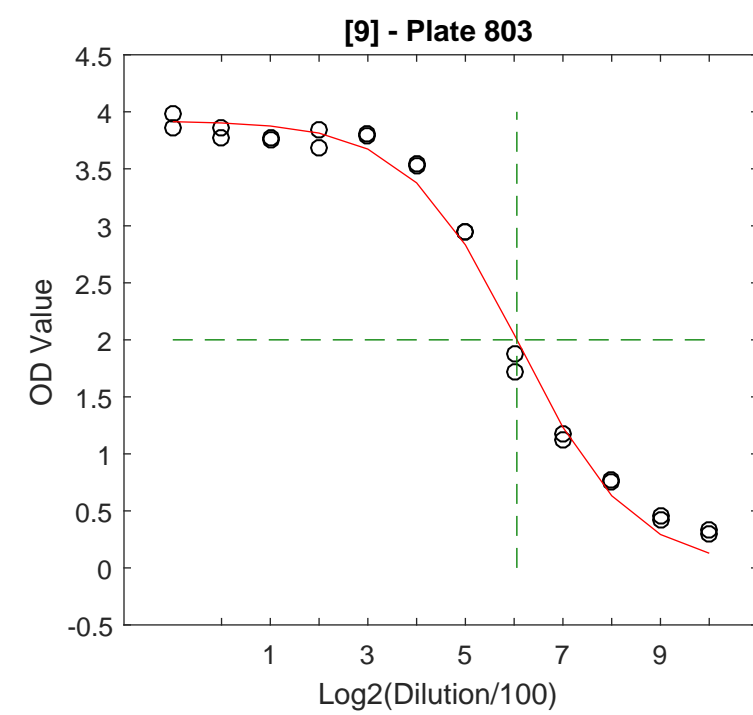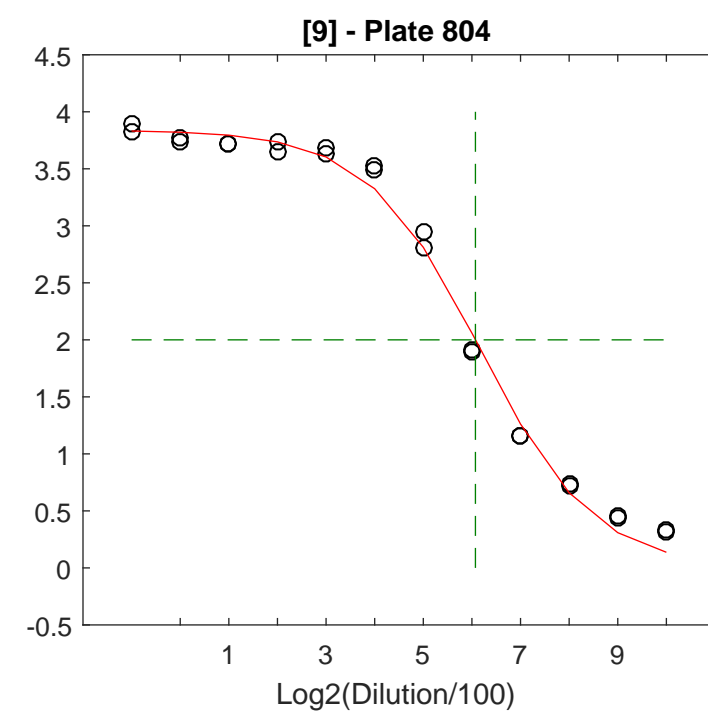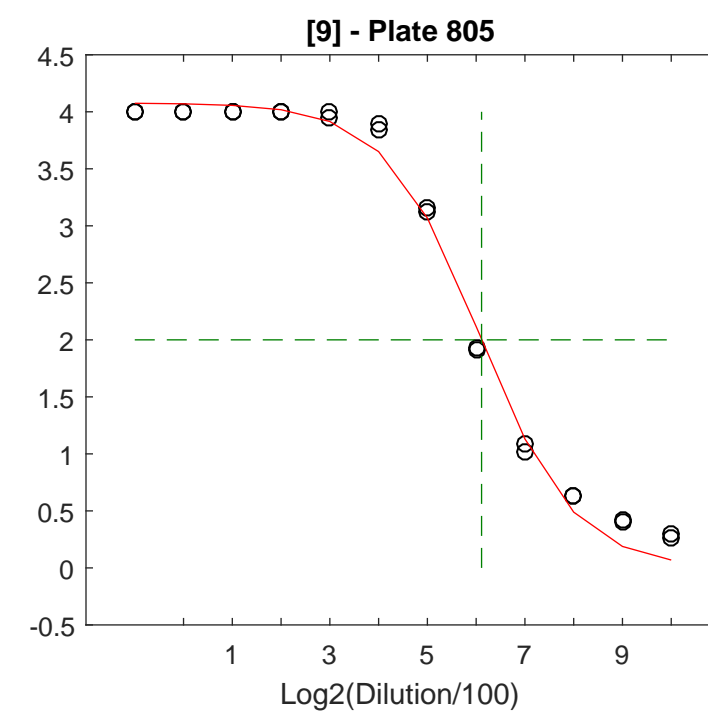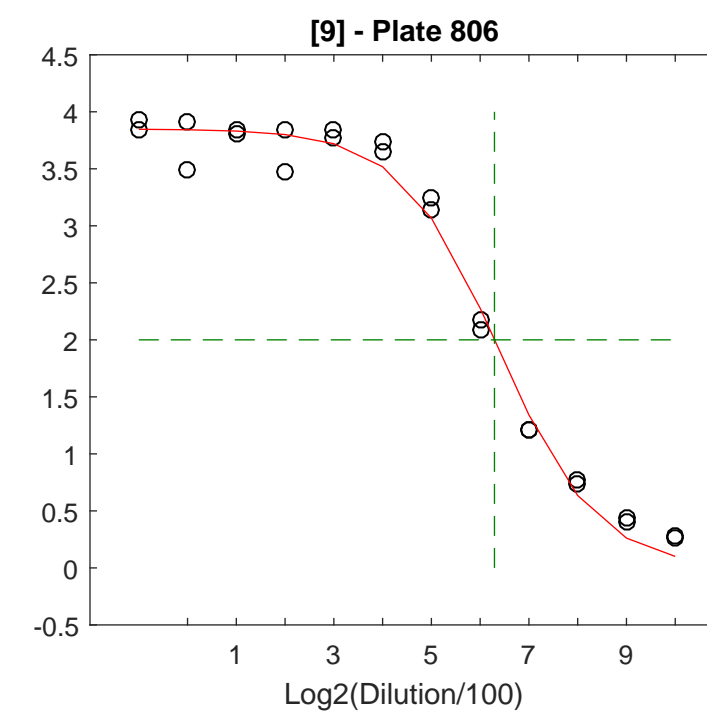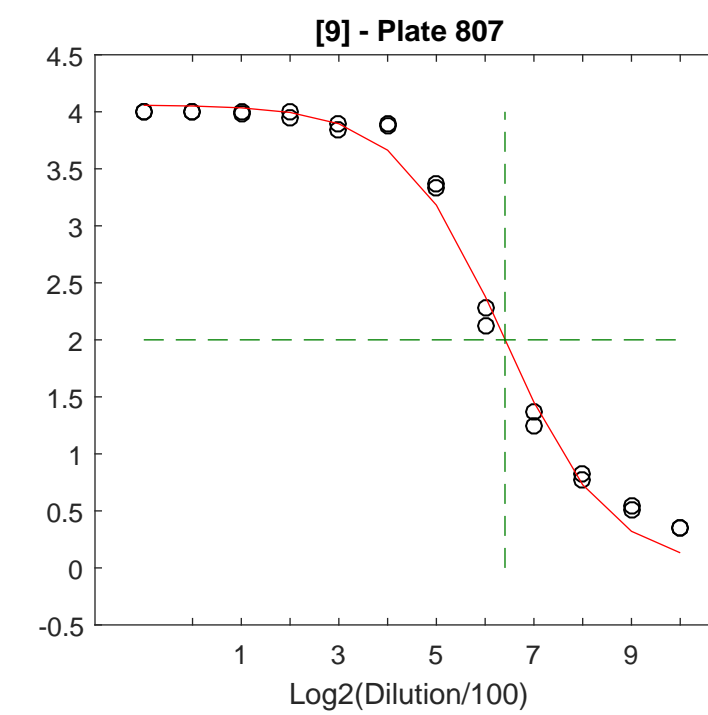

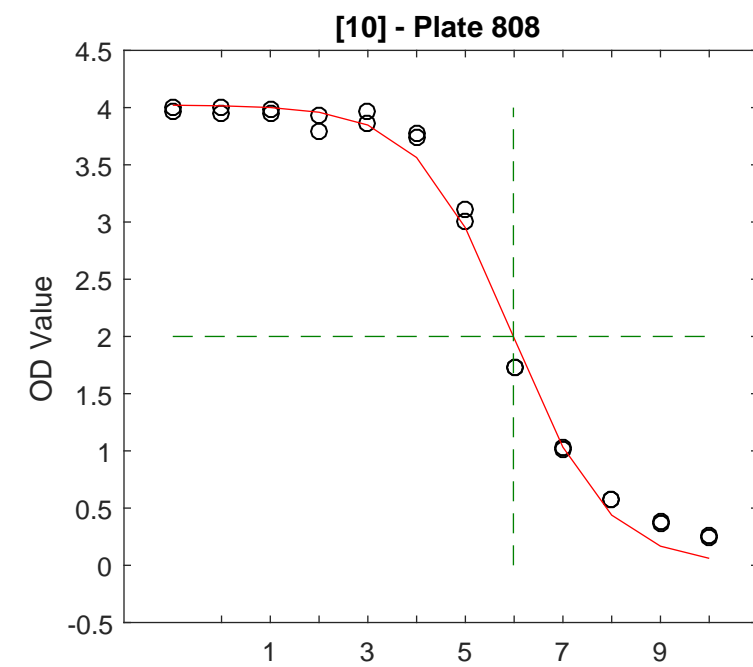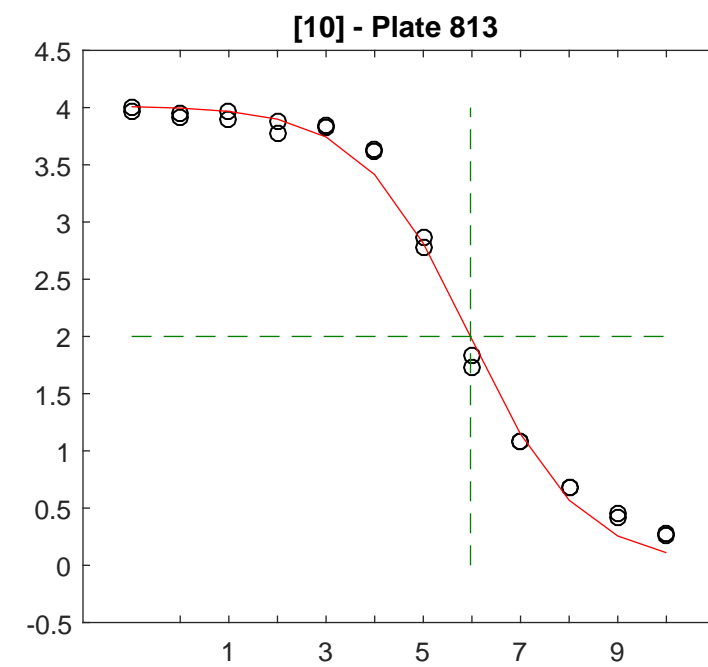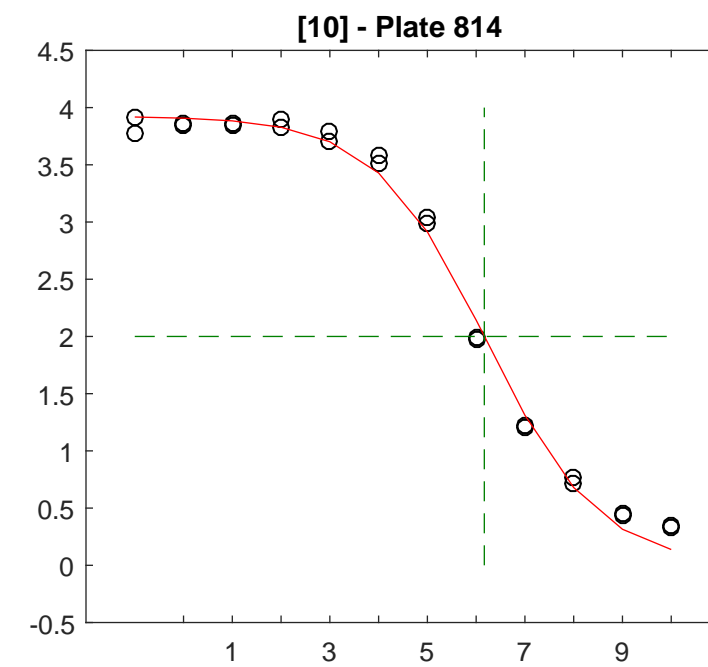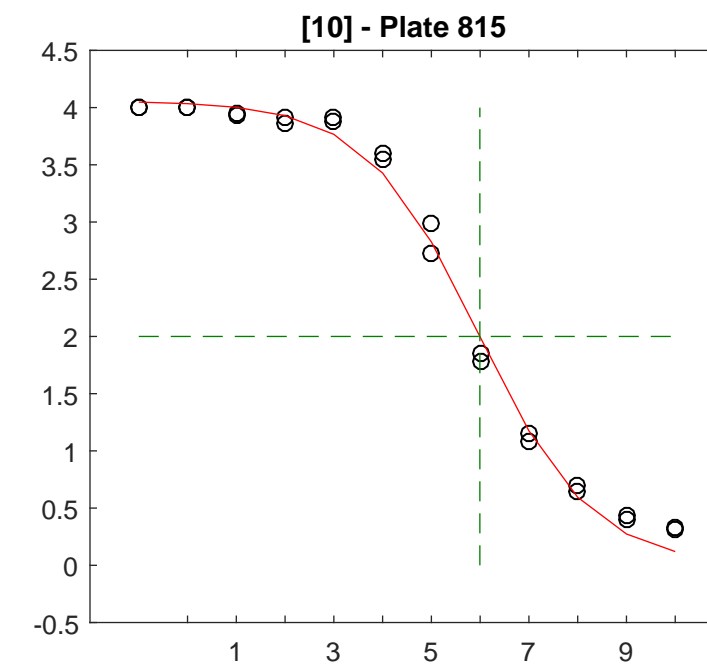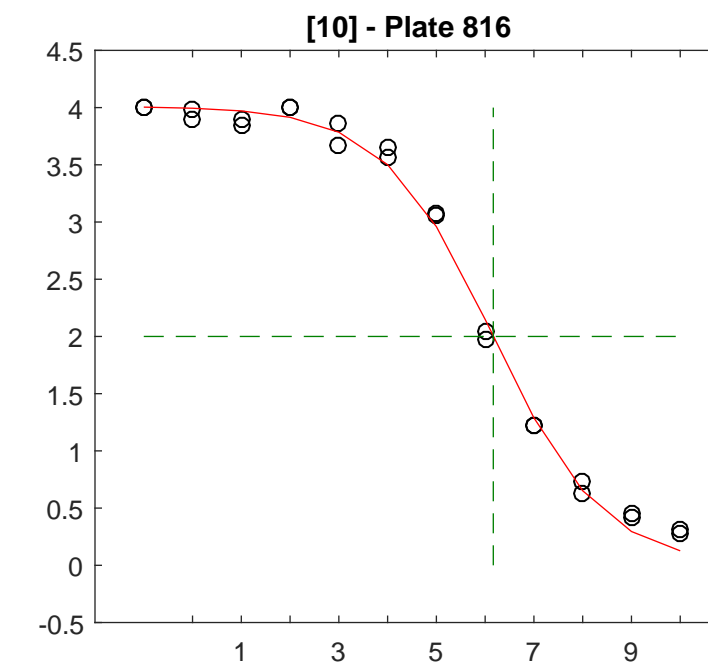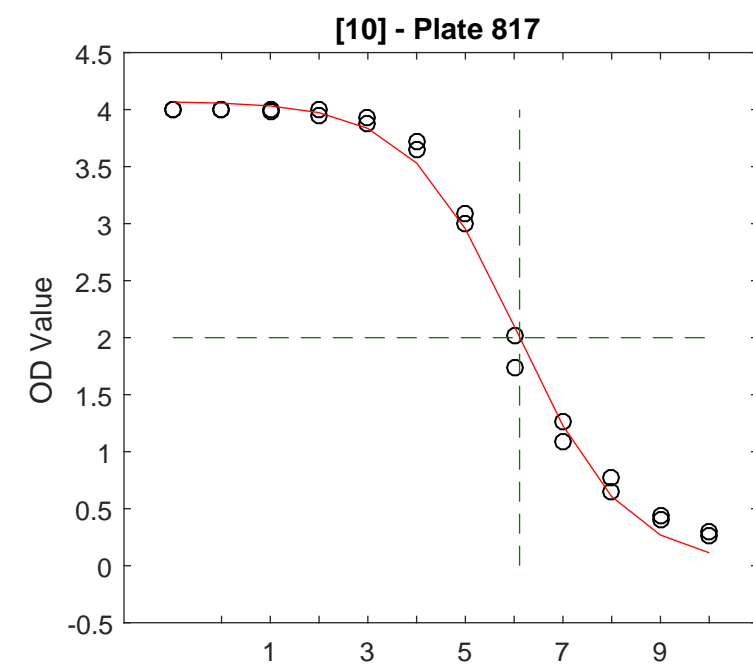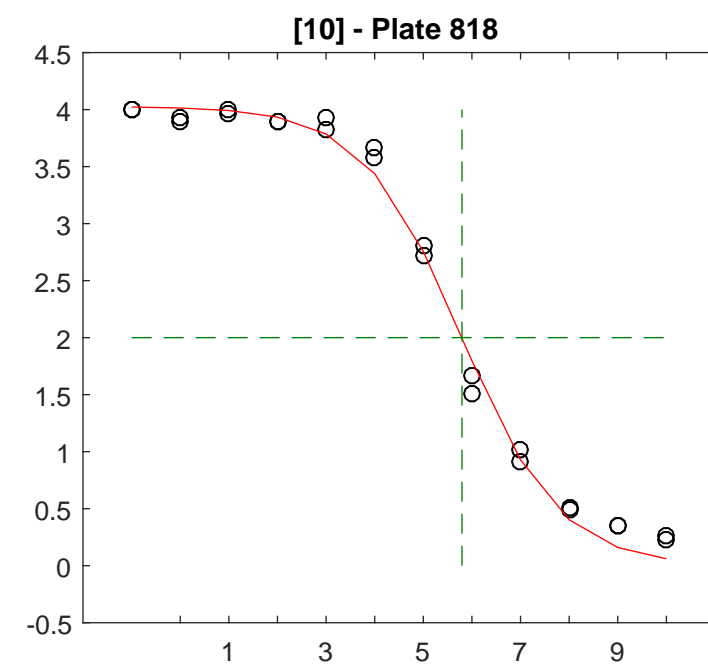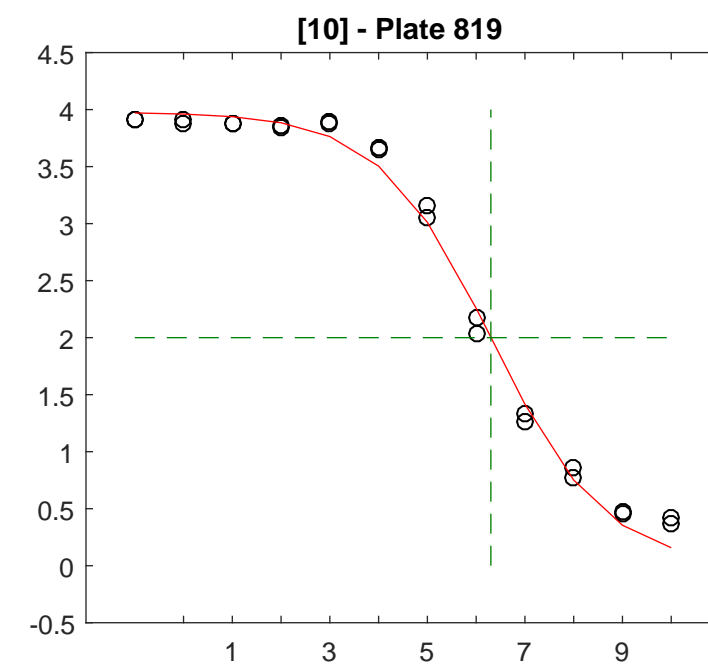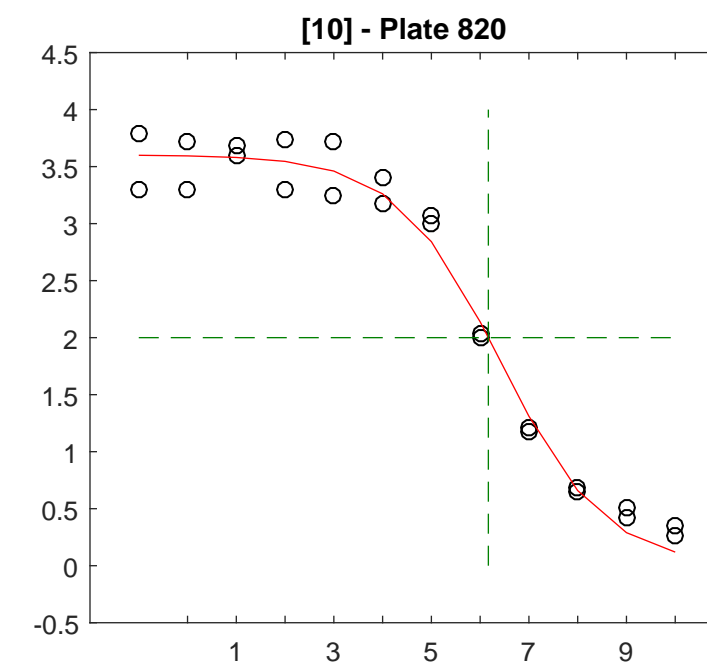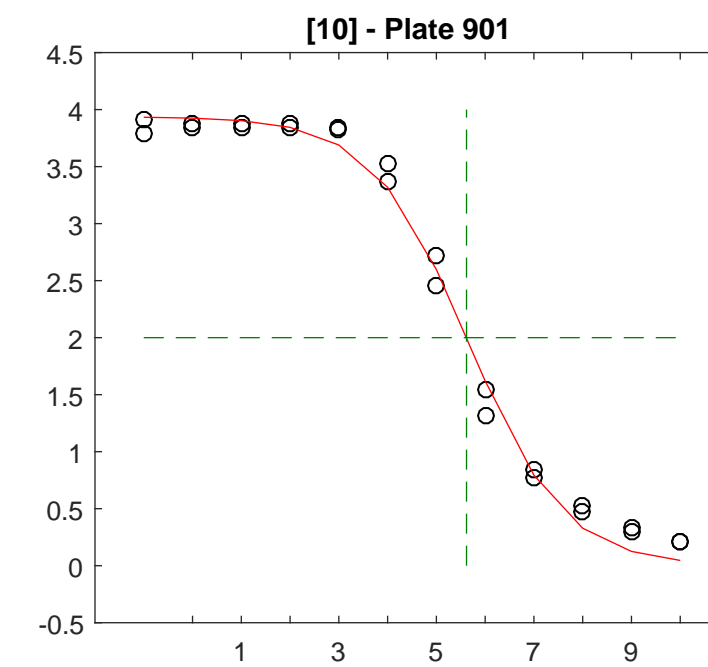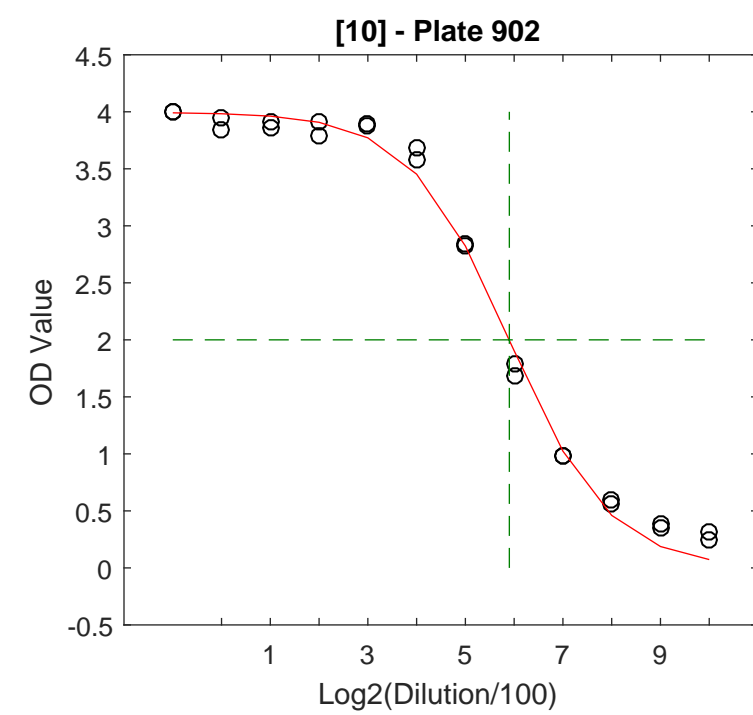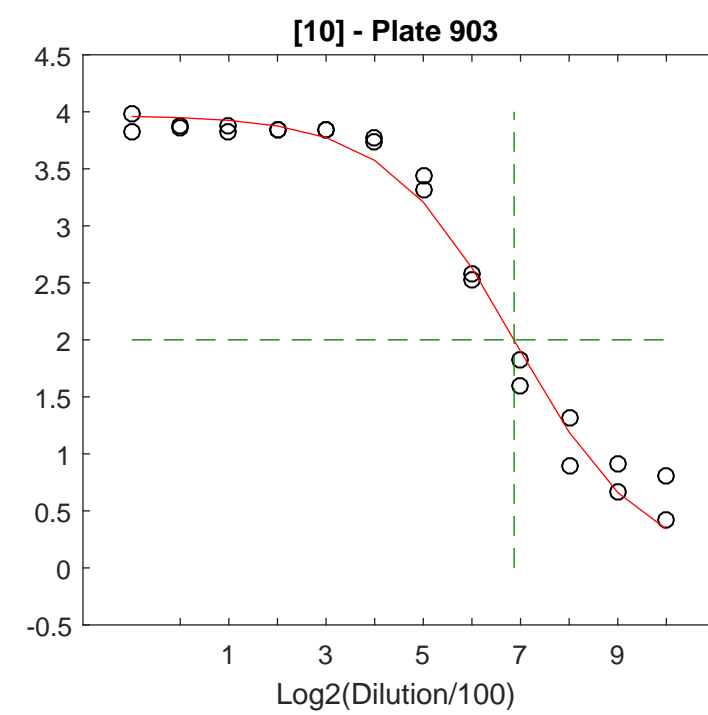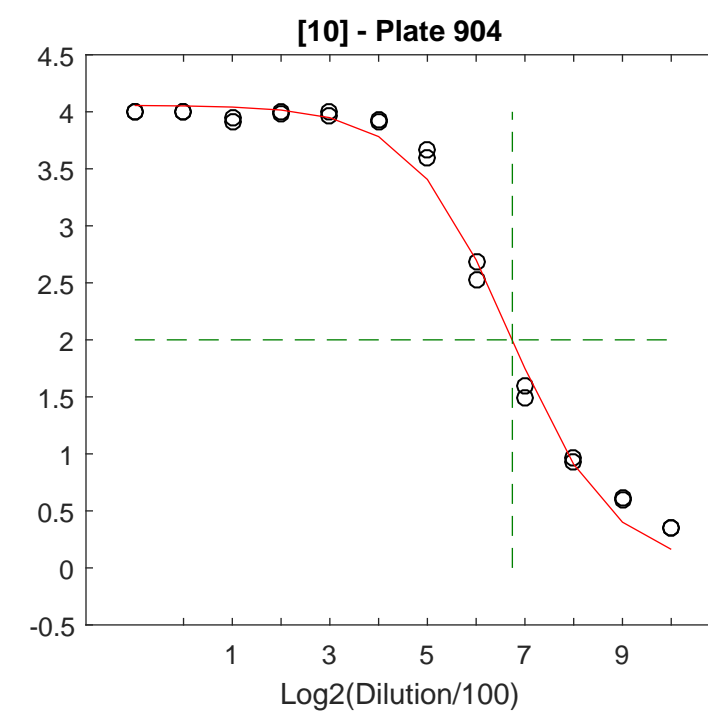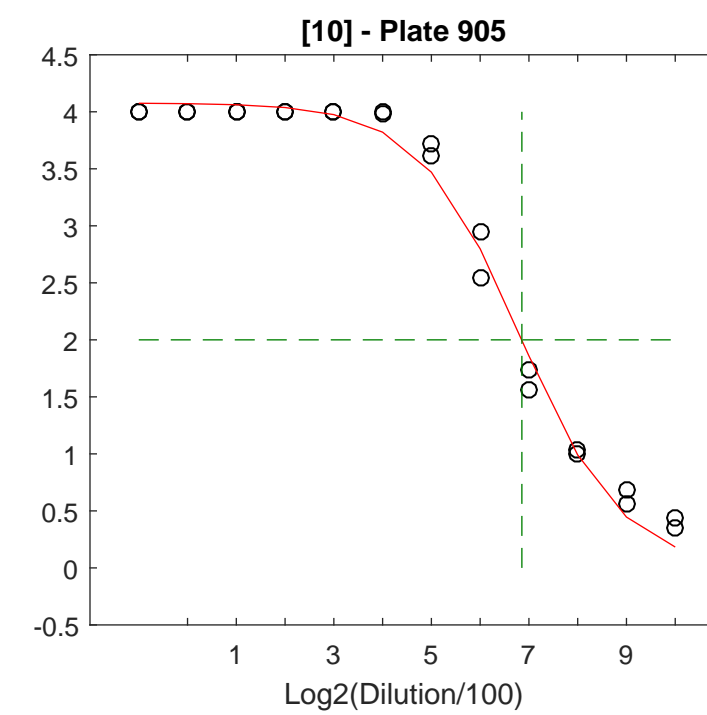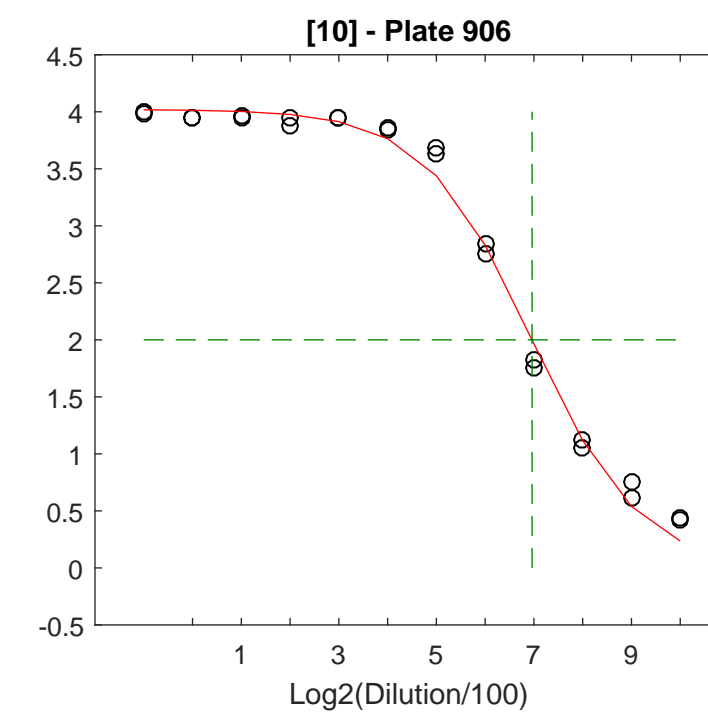

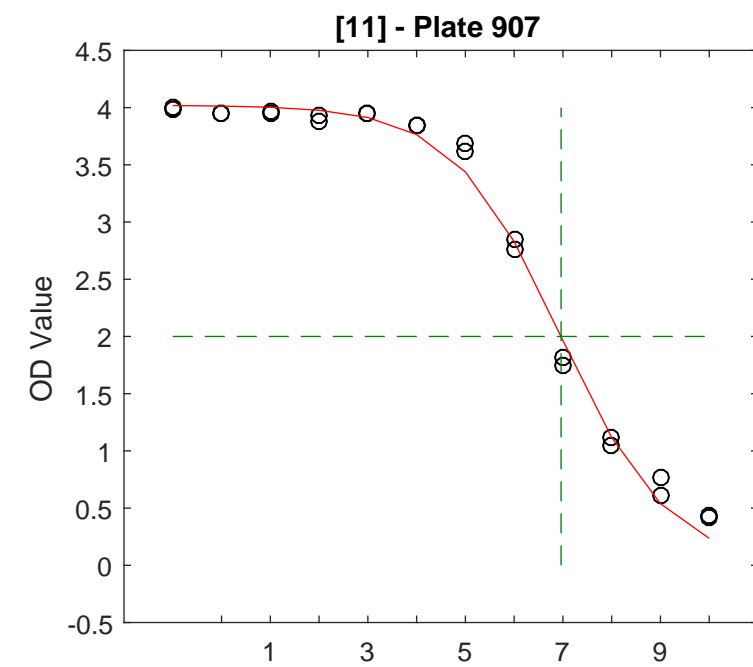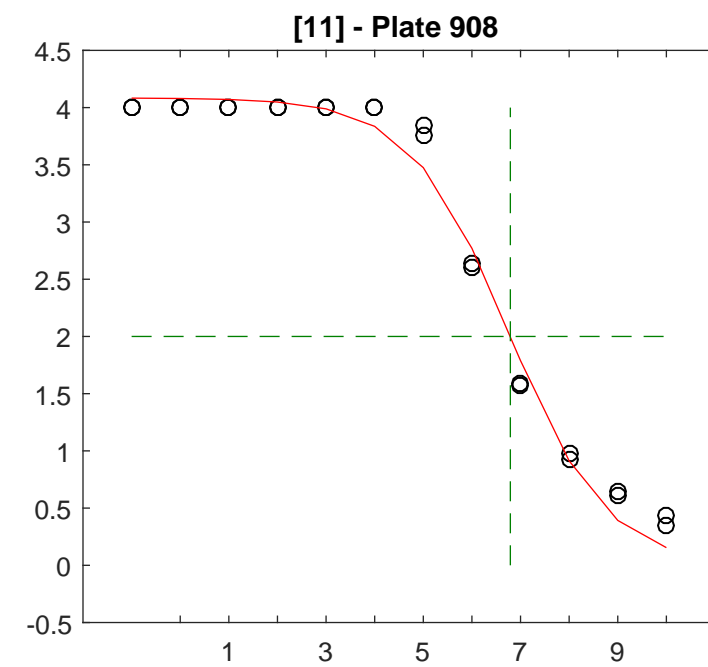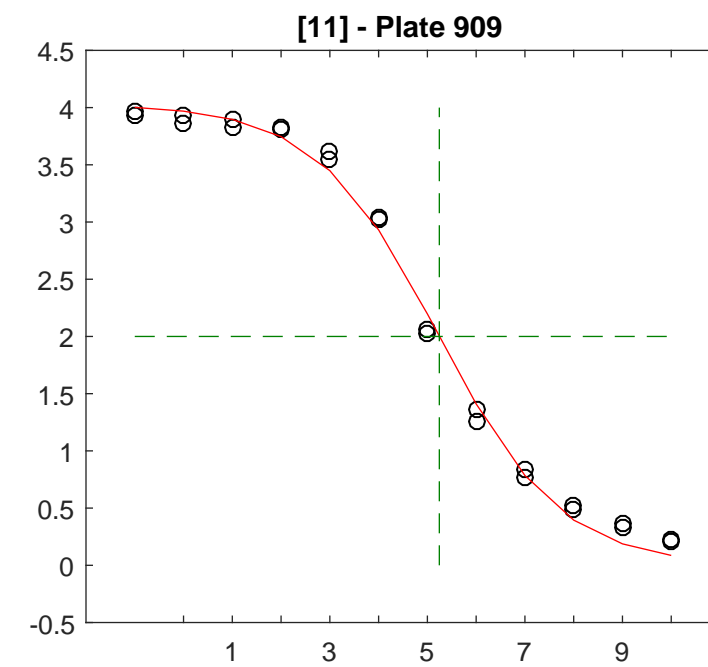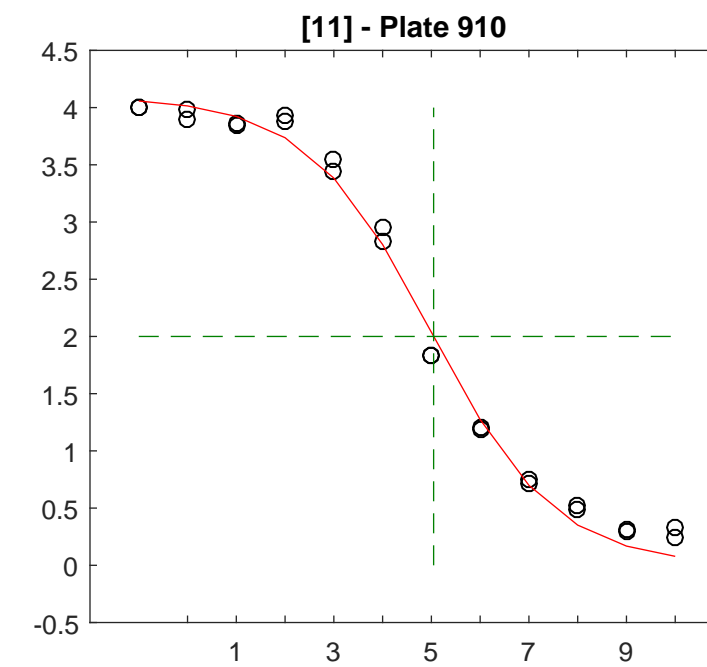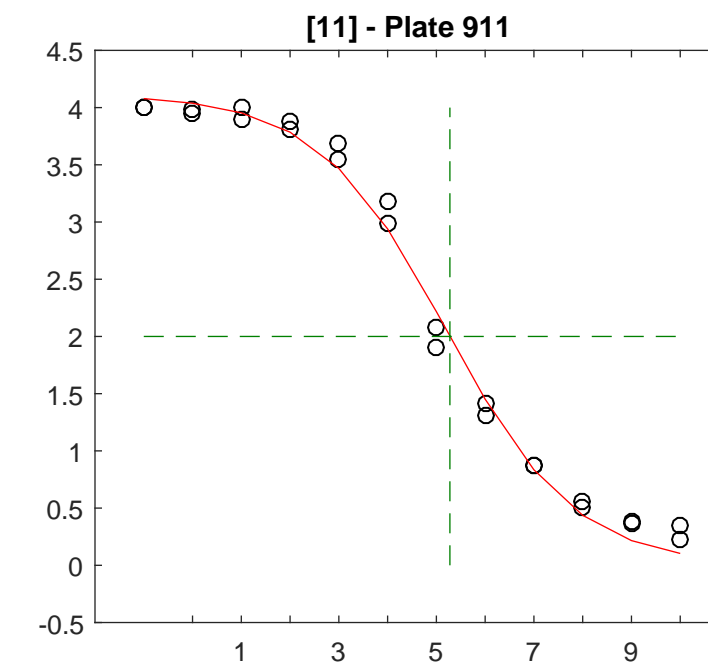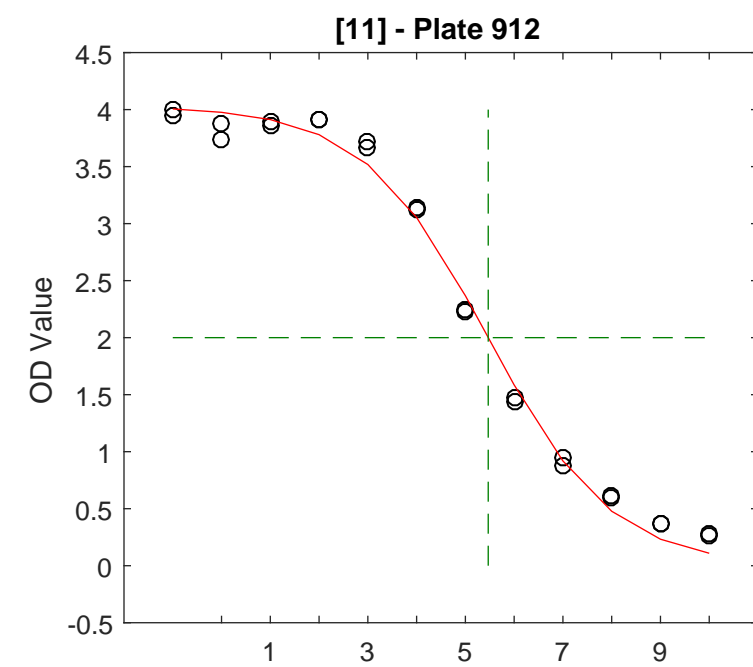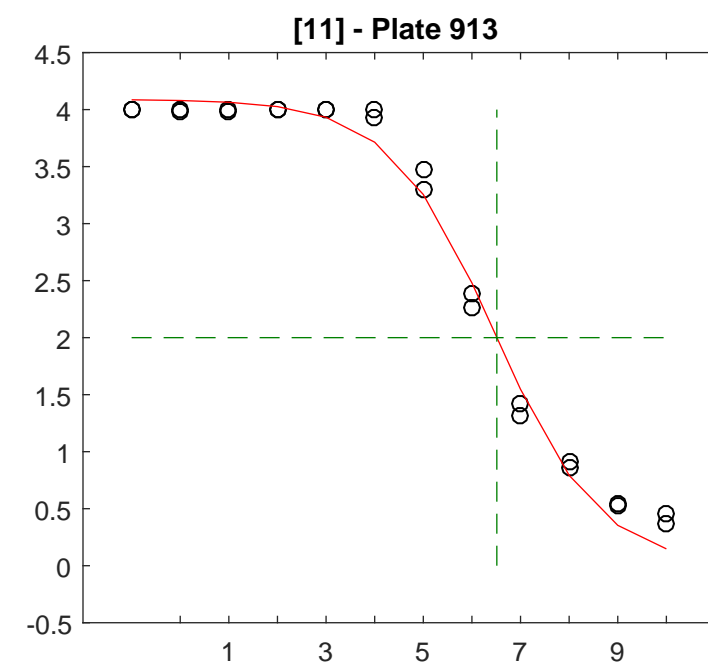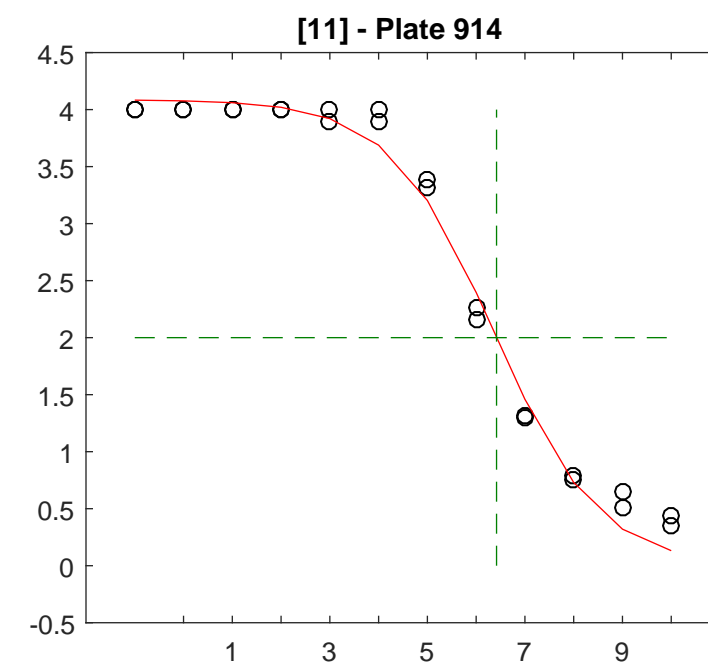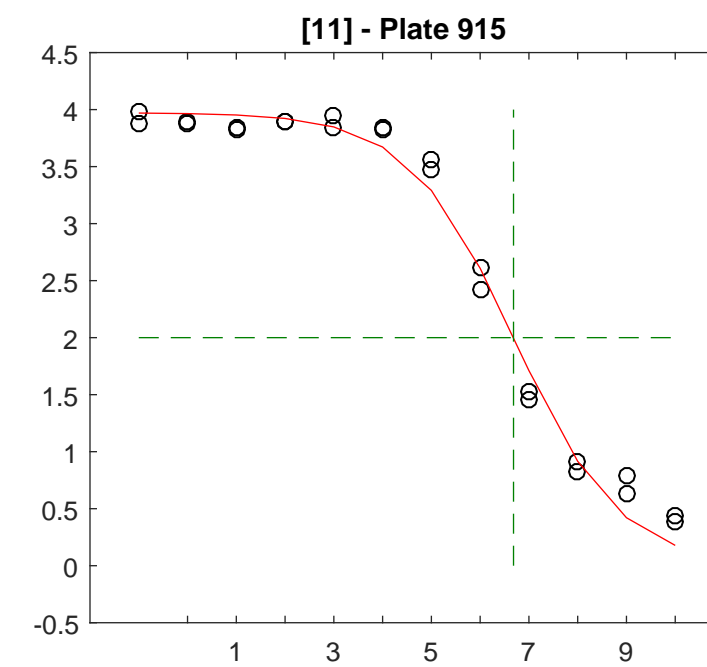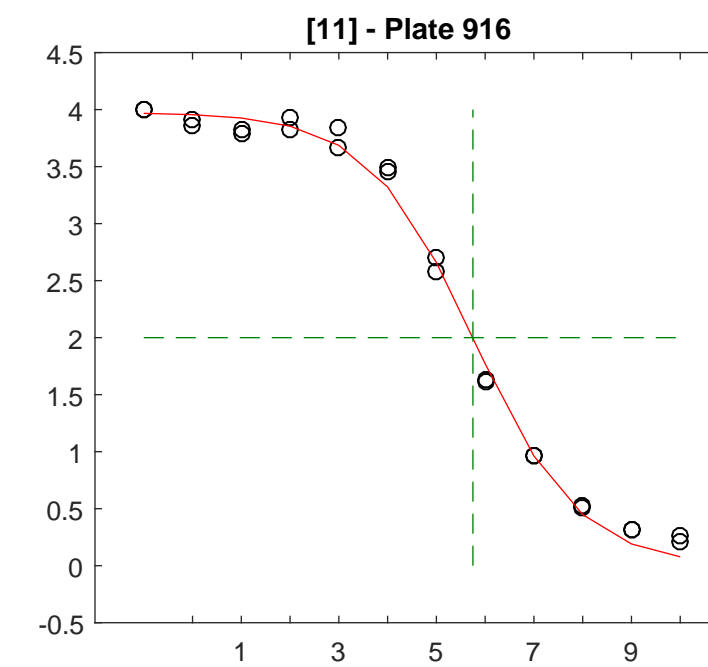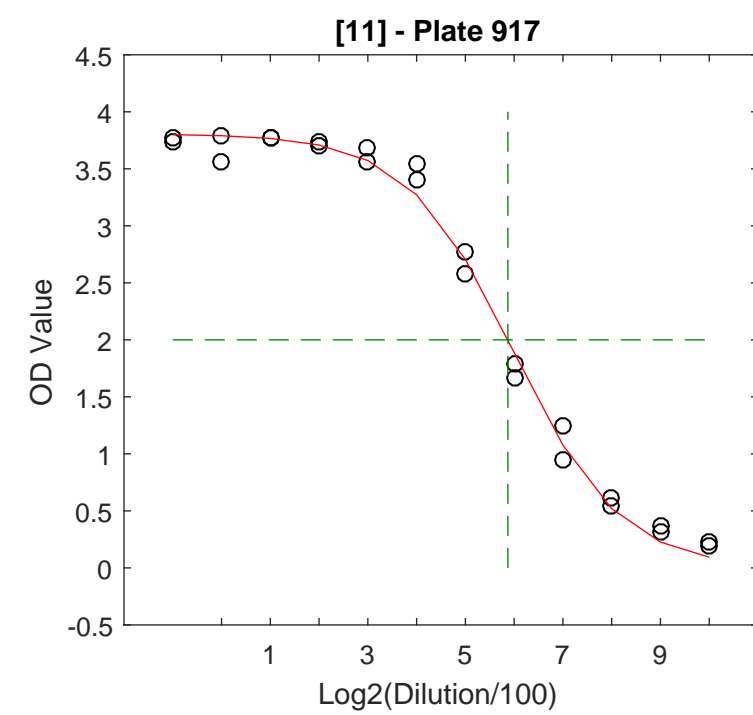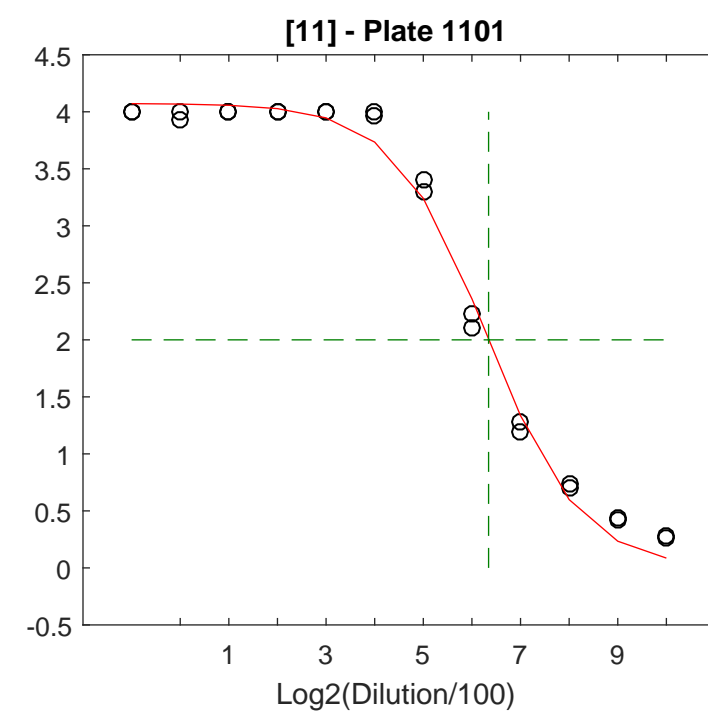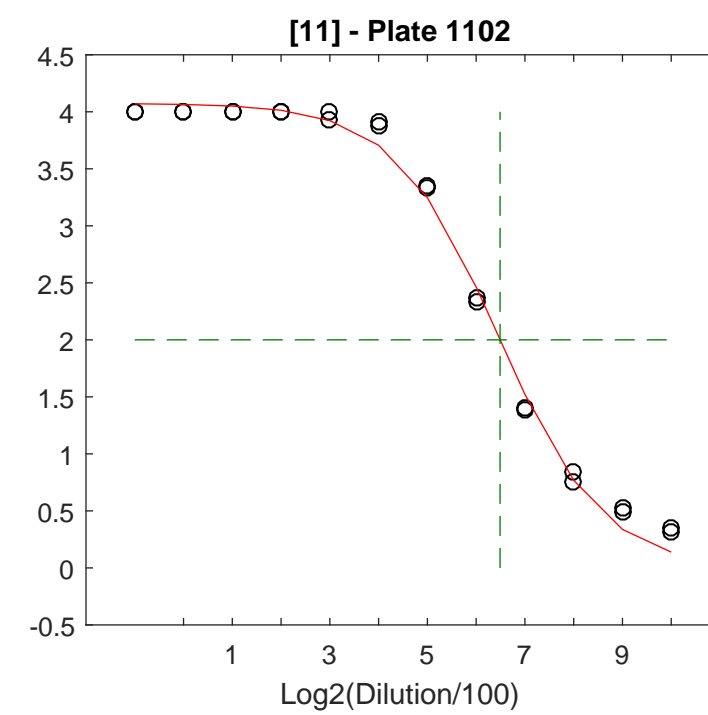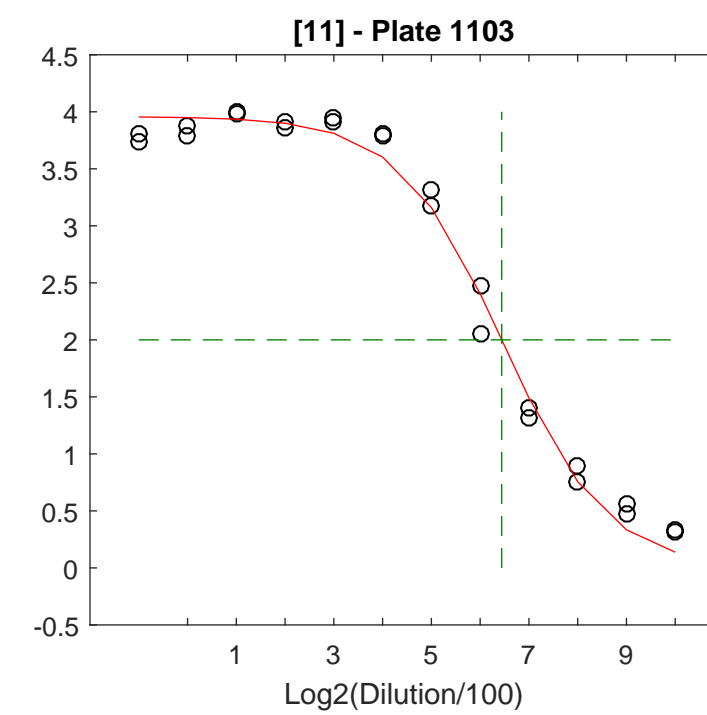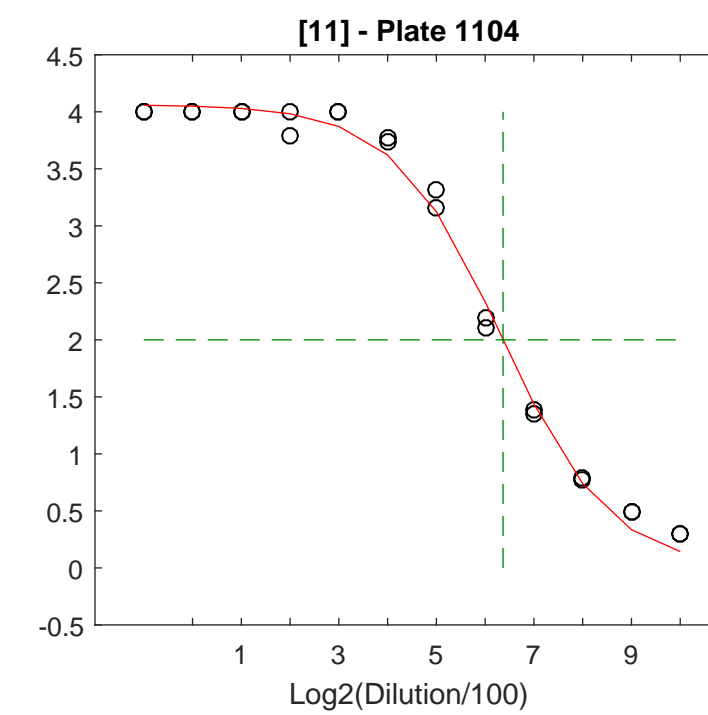

Supplement: Supplementary data 1 — Positive control OD curves. [file mmc1.pdf]
