# Supplementary material for: Seroprotection against tetanus in southern Vietnam
Source: Vaccine. 2023 Mar 24;41(13):2208–13. doi: 10.1016/j.vaccine.2023.02.036 (PMC10580288; doi:10.1016/j.vaccine.2023.02.036)
Supplement: Supplementary data 2 — Supplementary Tables, Figures and description of ELISA methodology. [file mmc2.docx]

**Supplementary materials**

**Descrition of ELISA methodology**

Wells of microtitre plates were coated with 0.5Lf/ml of tetanus toxoid (NIBSC) in carbonate-bicarbonate buffer (pH9.6). (Sigma C3041-100CAP). Plates were washed 3 times with PBST (Sigma P3563-5X10PAK). Vacant wells were blocked with 5% BSA in PBST and incubated at 37°C for 1 hour and rewashed. Serum samples were diluted in buffer (PBST containing 1% BSA Sigma A3803-100G) and tested at dilutions of 1/50, 1/100, 1/200. Positive controls were reference antitoxin 120 IU/ampoule with twelve 2-fold dilutions starting from 0.2 IU/ml to 10^-4^ IU/ml. Covered plates were incubated at 37°C for 2 hours and washed. Diluted rabbit anti-human IgG horseradish peroxidase (Sigma A-8792) was added to all the wells and plates were incubated for an hour at 37°C. After adding substrate (Sigma P9187-50SET) for 30 minutes, the reaction was stopped by 3M H_2_SO_4_. Plates were read at 490nm using an optical plate reader.

**Supplementary Table: Number of samples in selected age group by sex and province**

|  | 0.5- 4 years | | 6.9 - 12 years | | | 19.9- 34.9 years | | | 35- 90 years | |  | |
| --- | --- | --- | --- | --- | --- | --- | --- | --- | --- | --- | --- | --- |
|  | **Male** | **Female** | **Male** | **Female** | **Male** | | **Female** | **Male** | | **Female** | | ***Total*** |
| An Giang | 25 | 24 | 70 | 70 | 37 | | 38 | 69 | | 75 | | *408* |
| Binh Dinh | 10 | 5 | 69 | 69 | 10 | | 11 | 60 | | 62 | | *296* |
| Dak Lak | 32 | 18 | 70 | 70 | 35 | | 40 | 66 | | 55 | | *386* |
| Dong Thap | 7 | 4 | 35 | 97 | 33 | | 34 | 71 | | 79 | | *360* |
| HCMC | 25 | 25 | 70 | 70 | 37 | | 38 | 84 | | 73 | | *422* |
| Hue | 25 | 25 | 70 | 70 | 39 | | 35 | 76 | | 79 | | *419* |
| Khanh Hoa | 25 | 25 | 70 | 70 | 34 | | 41 | 78 | | 80 | | *423* |
| Kien Giang | 15 | 7 | 70 | 70 | 41 | | 34 | 76 | | 76 | | *389* |
| Quang Ngai | 26 | 24 | 95 | 53 | 38 | | 35 | 71 | | 86 | | *428* |
| Soc Trang | 20 | 23 | 60 | 53 | 29 | | 34 | 63 | | 51 | | *333* |
| *Total* | *210* | *180* | *679* | *692* | *333* | | *340* | *714* | | *716* | | *3864* |

**Supplementary Figure 1: Antibody concentrations in all adults (top panel), females (middle panel) and males (bottom panel)**


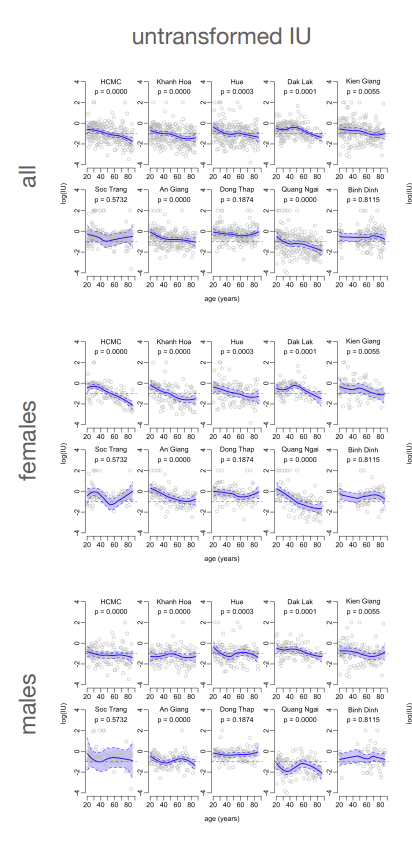


**Supplementary Figure 2: Effect of department on antibody concentrations** (Provinces where a significant difference in antibodies according to admitting ward are presented)


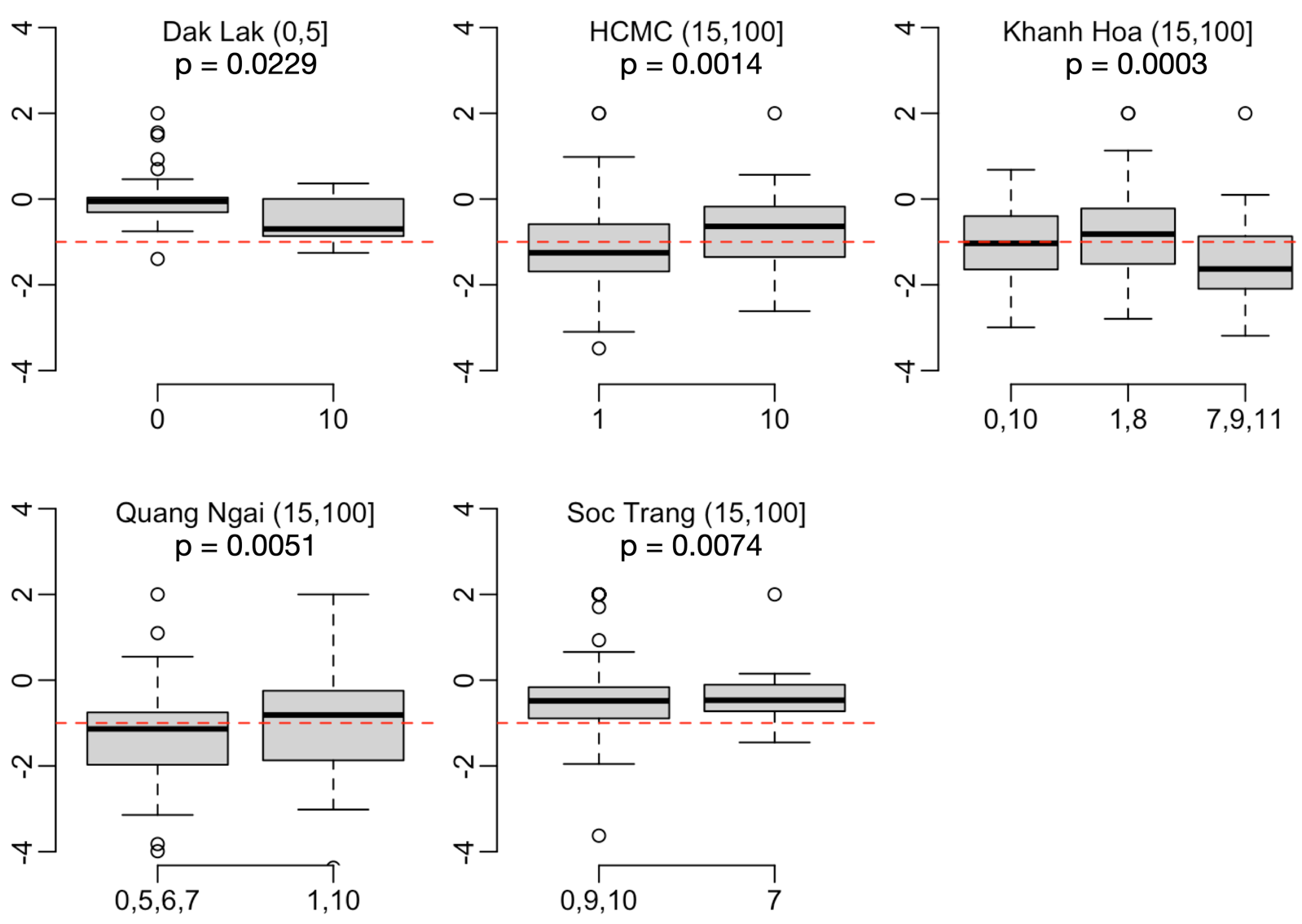


Figure showing anti-tetanus antibody levels (y axis in log_10_ IU/ml) according to age group and department: 0 -Outpatient, 1 General Infectious Disease Ward, 2 HIV Ward, 3 Malaria Ward, 4 Hepatitis Ward, 5 Repiratory/Chest Ward, 6 Gastroenterology Ward, 7 General Surgical Ward, 8 Trauma/Orthopedics, 9 Intensive Care Unit, 10 Other Ward (not listed in 0 through 9), 11 Unknown. For each site, we selected the combinations of age category and ward that had more than 10 data points. Then, for each age category of each site, we performed a recursive partitioning (1) of the data based on ANOVAs with IU as response and ward as explanatory variable.

(1) Breiman L., Friedman J. H., Olshen R. A., and Stone, C. J. (1984) Classification and Regression Trees. Wadsworth.
